# Glycyrrhizic Acid Derivatives Bearing Amino Acid Residues in the Carbohydrate Part as Dengue Virus E Protein Inhibitors: Synthesis and Antiviral Activity

**DOI:** 10.3390/ijms231810309

**Published:** 2022-09-07

**Authors:** Mann-Jen Hour, Yeh Chen, Chen-Sheng Lin, Lidia A. Baltina, Ju-Ying Kan, Yan-Ting Tsai, Yan-Tung Kiu, Hsueh-Chou Lai, Lia A. Baltina, Svetlana F. Petrova, Cheng-Wen Lin

**Affiliations:** 1 School of Pharmacy, China Medical University, Taichung 40402, Taiwan; 2 Institute of New Drug Development, China Medical University, Taichung 40402, Taiwan; 3 Division of Gastroenterology, Kuang Tien General Hospital, No. 117 Shatian Road, Shalu District, Taichung 43303, Taiwan; 4 Ufa Institute of Chemistry, Ufa Federal Research Centre of RAS, 71 Prospect Oktyabrya, 450054 Ufa, Russia; 5 Graduate Institute of Biomedical Sciences, China Medical University, 91, Hsueh-Shin Road, Taichung 40402, Taiwan; 6 Department of Medical Laboratory Science and Biotechnology, China Medical University, 91 Hsueh-Shih Road, Taichung 40402, Taiwan; 7 Department of Medical Laboratory Science and Biotechnology, Asia University, 500 Lioufeng Road, Wufeng, Taichung 41354, Taiwan; 8 School of Chinese Medicine, China Medical University, 91 Hsueh-Shih Road, Taichung 40402, Taiwan

**Keywords:** Glycyrrhizic acid, derivatives, amino acids, methyl/ethyl ester, synthesis, antiviral activity, Dengue virus, molecular model

## Abstract

Dengue virus (DENV) is one of the most geographically distributed mosquito-borne flaviviruses, like Japanese encephalitis virus (JEV), and Zika virus (ZIKV). In this study, a library of the known and novel Glycyrrhizic acid (GL) derivatives bearing amino acid residues or their methyl/ethyl esters in the carbohydrate part were synthesized and studied as DENV inhibitors in vitro using the cytopathic effect (CPE), viral infectivity and virus yield assays with DENV1 and DENV-2 in Vero E6 and A549 cells. Among the GL conjugates tested, compound hits GL-D-ValOMe **3**, GL-TyrOMe **6**, GL-PheOEt **11**, and GL-LysOMe **21** were discovered to have better antiviral activity than GL, with IC50 values ranging from <0.1 to 5.98 μM on the in vitro infectivity of DENV1 and DENV2 in Vero E6 and A549 cells. Compound hits **3, 6, 11,** and **21** had a concentration-dependent inhibition on the virus yield in Vero E6, in which GL-D-ValOMe **3** and GL-PheOEt **11** were the most active inhibitors of DENV2 yield. Meanwhile, the time-of-addition assay indicated that conjugates GL-D-ValOMe **3** and GL-PheOEt **11** exhibited a substantial decrease in the DENV2 attachment stage. Subsequently, chimeric single-round infectious particles (SRIPs) of DENV2 C-prM-E protein/JEV replicon and DENV2 prM-E/ZIKV replicon were utilized for the DENV envelope I protein-mediated attachment assay. GL conjugates **3** and **11** significantly reduced the attachment of chimeric DENV2 C-prM-E/JEV and DENV2 prM-E/ZIKV SRIPs onto Vero E6 cells in a concentration-dependent manner but did not impede the attachment of wild-type JEV CprME/JEV and ZIKV prM-E/ZIKV SRIPs, indicating the inhibition of Compounds **3** and **11** on DENV2 E-mediated attachment**.** Molecular docking data revealed that Compounds **3** and **11** have hydrophobic interactions within a hydrophobic pocket among the interfaces of Domains I, II, and the stem region of the DENV2 envelope (E) protein. These results displayed that Compounds **3** and **11** were the lead compounds targeting the DENV E protein. Altogether, our findings provide new insights into the structure–activity relationship of GL derivatives conjugated with amino acid residues and can be the new fundamental basis for the search and development of novel flavivirus inhibitors based on natural compounds.

## 1. Introduction

The emergence and worldwide spread of socially dangerous arboviral infections transmitted by mosquito-vector have led to a great problem in the health care of many countries. Currently, more than 300 million cases of arbovirus infections (Yellow fever virus, Dengue virus (DENV), Zika virus (ZIKV), Chikungunya virus, Japanese encephalitis virus (JEV), West Nile virus (WNV), etc.) are registered worldwide [1,2,3]. Dengue virus (DENV) spreads by the bite of mosquitoes *Aedes sps. (Aedes aegypti and A. albopictus)* and is one of the most geographically distributed flaviviruses belonging to the *Flaviviridae* family and to the ecological group of arboviruses [4,5,6]. DENV is posing a threat to the health of the population of more than 100 countries around the world, is common in countries and territories with a tropical and subtropical climate, and 50–100 million dengue viral infection cases are registered annually including 24,000 deaths, and 2.5–3 billion people are at risk of infection [6,7,8,9]. In the last 50 years, the incidence of dengue disease has increased by 30 times due to the geographical expansion in new countries and DENV was declared by the World Health Organization as the main viral threat to humanity [7,8,9]. The number of imported dengue cases in Europe has increased by 21–25%, especially in the UK, Germany and France [10].

The DENV genome consists of a single-stranded long open chain encoding three structural proteins (capsid C, membrane protein prM/M, envelope glycoprotein E) and seven non-structural proteins (NS1, NS2A, NS2B, NS3, NS4A, NS4B, NS5) [5,11]. DENV has been divided into four serotypes (DENV-1—DENV-4) that cause dengue fever, dengue hemorrhagic fever and dengue shock syndrome, which is fatal [12,13]. DENV2 infection is manifested as a hemorrhagic fever, which is characterized by high blood plasma levels of TNF, TNFR1, TNFR2, IFN, CXCL8, CXCL9, CXCL10, CXCL11, CCL5, VEGFA and IL-10 [6,13]. Infection caused by one DENV serotype provides long-term resistance to this serotype but does not lead to the development of immunity from other serotypes [12,14]. Currently, there are no licensed tetravaccines against all the DENV serotypes, and only one licensed Dengvaxia vaccine is in the pharmaceutical market that protects against DENV1 and DENV2 [15]. Antiviral drugs for dengue virus-specific chemotherapy are not developed either [16,17]. The following targets are investigated in the search for new antiviral agents against DENV: envelope (E) protein, protease NS2B/NS3, helicase, methyltransferases, RNA-dependent RNA polymerase, R-glucosidase and non-structured proteins [18,19,20,21,22,23]. NS3 serine-protease plays a key role in the life cycle of DENV and is an ideal target for the design of new antiviral agents [21]. RNA-dependent RNA polymerase (RdRp) of flaviviruses is used as a target for the development of new antiviral agents and is responsible for initiating and catalyzing the synthesis of viral RNA in the cytoplasm of infected cells [12,16].

**Figure 1:**
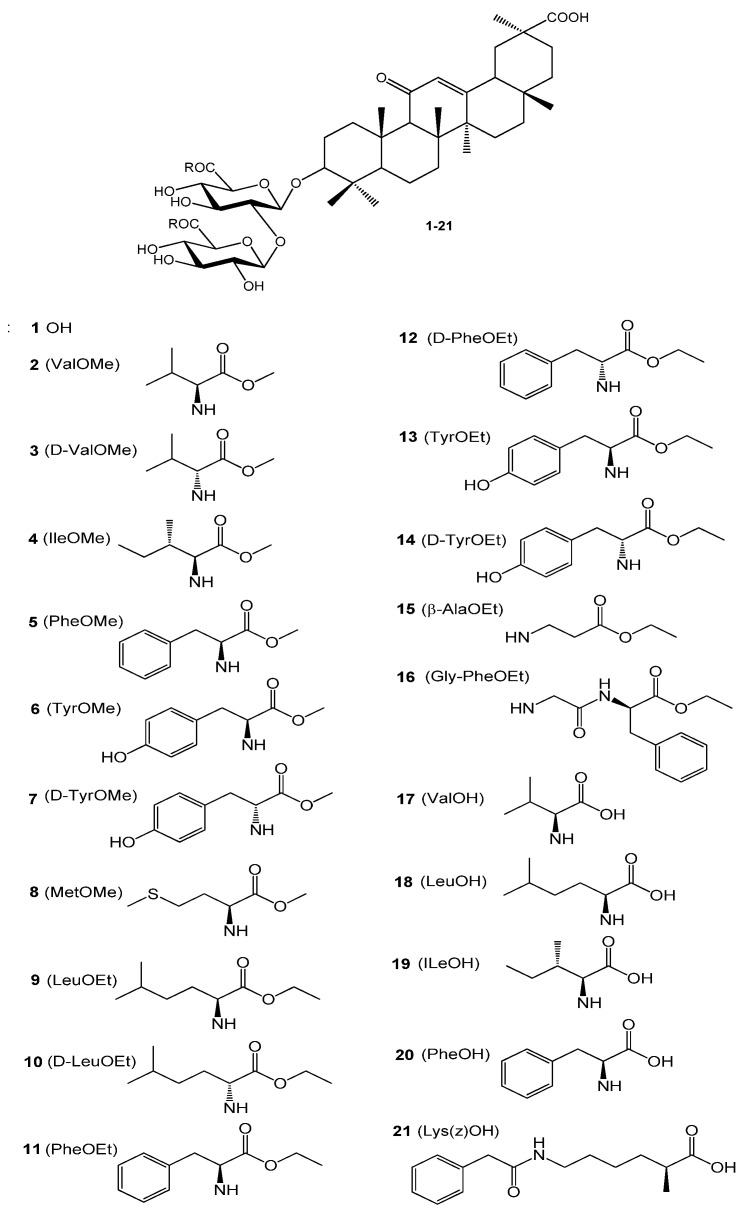
Structure of Glycyrrhizic acid and its conjugates **2**–**21**.

The modern pharmaceutical market has a limited group of antiviral drugs, most of which are synthetic molecules or nucleoside analogs that are used to treat clinically significant viral infections but have side effects and cause drug resistance [24]. Therefore, the search and development of new antiviral agents for the control and therapy of dengue infection is one of the urgent problems of modern medicinal chemistry and virology, which is provided for research and development programs in many countries. One of the modern approaches in the development of new antiviral agents is the search for antivirals among the plant-derived natural products (plant secondary metabolites), which are an inexhaustible source for the new lead compound and drug candidates to control and treat viral and microbial infections [25,26]. A natural source for obtaining the new potential antiviral agents is represented by the well-known licorice roots (*Glycyrrhiza glabra*, *Gl. uralensis Fisher*) (*Leguminosae*), an ancient medicinal plant of the East and West traditional medicines [27,28,29]. A major bioactive component (7–24%) of licorice roots is Glycyrrhizic acid (GL) **1** (Figure 1), one of the leading natural triterpene glycosides promising as a scaffold for the development of new antiviral agents [28,29,30,31,32,33]. GL inhibits many types of DNA and RNA viruses (Varicella-zoster, Herpes simplex type 1, influenza viruses, cytomegaloviruses, hepatitis B and C, etc.) [30,31,34,35], and is used clinically for the treatment of chronic viral hepatitis B and C in Japan and China (SNMC preparation, Compound Glycyrrhizin Ingecpion Injection, Compound Glycyrrhizin Ingecpion Tablets) [31,32,36]. GL inhibits the reproduction of HIV-1 in vitro and in vivo and is suitable for the long-term therapy of HIV infection in a combination with other anti-HIV agents [37,38]. GL affects cellular factors such as protein kinase II, casein kinase II and transcription factors (activating protein I and nuclear factor kB) [31,32,33]. Chemical modification of GL is a promising way to obtain new antiviral agents [32,33,39]. We synthesized a series of GL derivatives, which exceed the natural glycoside by anti-HIV activity [32,39]. The inhibitory effect was found for GL derivatives in vitro against the SARS-associated coronaviruses [40], Epstein-Barr virus [41], and influenza A/H1N1 virus [42,43]. GL inhibits several pathogenic flaviviruses such as yellow fever virus and DENV at high non-toxic concentrations [44]. Recently, we reported that GL and some of its derivatives had high antiviral activity against DENV2 [45] and ZIKV [46]. The antiviral activity of GL and its derivatives is associated mainly with the ability to potentiate ϒ-interferon production in vitro and in vivo [31,32,33]. The stimulating effect of GL was noted on the secretion of IL-2, inducing the production of interferon by peripheral lymphocytes [31,32]. Thus, GL is suitable for the prevention of many viral infections [29,30,31,32,33,36], low-toxic to mammals (LD50 5000 mg/kg) [28], and metabolizes in the gastrointestinal tract and liver as corticosteroid hormones [28,32].

In the present article, we performed the synthesis and antiviral activity studies of a library of previously synthesized and novel GL conjugates with amino acids and their methyl/ethyl eaters as DENV inhibitors. We showed that the introduction of amino acids or their methyl/ethyl esters in the carbohydrate part of GL strongly exhibited a better anti-viral activity of GL derivatives on the infectivity and virus yield of DENV1 and DENV2 in a comparison with a native scaffold, GL. The compound leads of GL derivatives identified in this study were demonstrated to block the DENV E protein-mediated attachment, which was supported by the molecular docking of active GL derivatives into the hydrophobic pocket formed among Domains I and II and the stem region of the DENV E protein dimer structure. The presented results may become a scientific basis for the development of new antiviral agents based on natural compounds for the treatment of socially dangerous flaviviral infections.

## 2. Results

### 2.1. Synthesis of GL Conjugates with Amino Acids and Their Methyl/Ethyl Esters

In search of new DENV inhibitors, we synthesized a library of 20 known and novel GL derivatives bearing methyl/ethyl esters of L- and D-amino acids, and free amino acids in the carbohydrate part of the glycoside. The structures of the GL conjugates with amino acids and their methyl/ethyl esters are given in Figure 1. The GL derivatives used in this study may be divided into three groups: Compounds **2**–**8** are GL conjugates containing two L- or D-amino acids methyl esters; group II (Compounds **9**–**16**) is presented by GL conjugates containing two residues of ethyl esters L- or D-amino acids, and dipeptide Gly-PheOEt; group III includes GL conjugates with free L-amino acids **17**–**21.** Compounds **2 [42]**, **3 [43]**, **5 [42]**, **6 [42,46]**, **8 [47]**, **9 [46], 11 [46], 16 [46]**, **17**–**20 [46,48,49]**, and **21 [46,50]** were previously described and their analytical and spectral data were identical to the published data. Conjugates **2**–**5** with methyl esters of amino acids were produced using 1-ethyl-3-(3-dimethylaminopropyl)carbodiimide hydrochloride (EDC) [48] in dimethylformamide (DMF) in the presence of an excess of triethyl amine at room temperature (20–22 °C), showing yields of 52–55% after purification by column chromatography (CC). Compounds **6**–**16** were prepared by the selective activation of COOH groups of the GL glucuronide part with HOSu/DCC [46,49,51] in DMF with yields of 55–60%. GL conjugates with free amino acids **17**–**21** were synthesized by a new simplified procedure using amino acids as Na salts in a mixture of 1N aqueous NaOH and DMF with yields of 54–56% after the purification by CC. Earlier, we prepared GL conjugates with free amino acids by using tert-butyl esters of amino acids with the subsequent release with CF_3_COOH [48,49] with similar yields. All compounds produced had a purity of ≥95% according to HPLC analysis (Supplementary Figures S1–S20). Analytical and spectral data for GL derivatives **2 [42]**, **3 [43]**, **5 [42]**, **6 [42,46]**, **8 [47]**, **9 [46], 11 [46], 16 [46]**, **17**–**20 [46,48,49]**, and **21 [46,50]** were identical to the previously published data but their ^1^H NMR spectra (500 MHz) are given firstly in this paper. The structures of the novel Compounds **4, 7, 10, 12**–**14, and 15** were confirmed by IR and NMR (^1^H-500 MHz and ^13^C-125 MHz) spectra and elemental analysis. The NMR spectra for the novel most active GL derivatives are given in Supplementary Materials (Supplemental Figures S1–S23).

### 2.2. Antiviral Screening of GL Derivatives in CPE Assay

**Figure 2:**
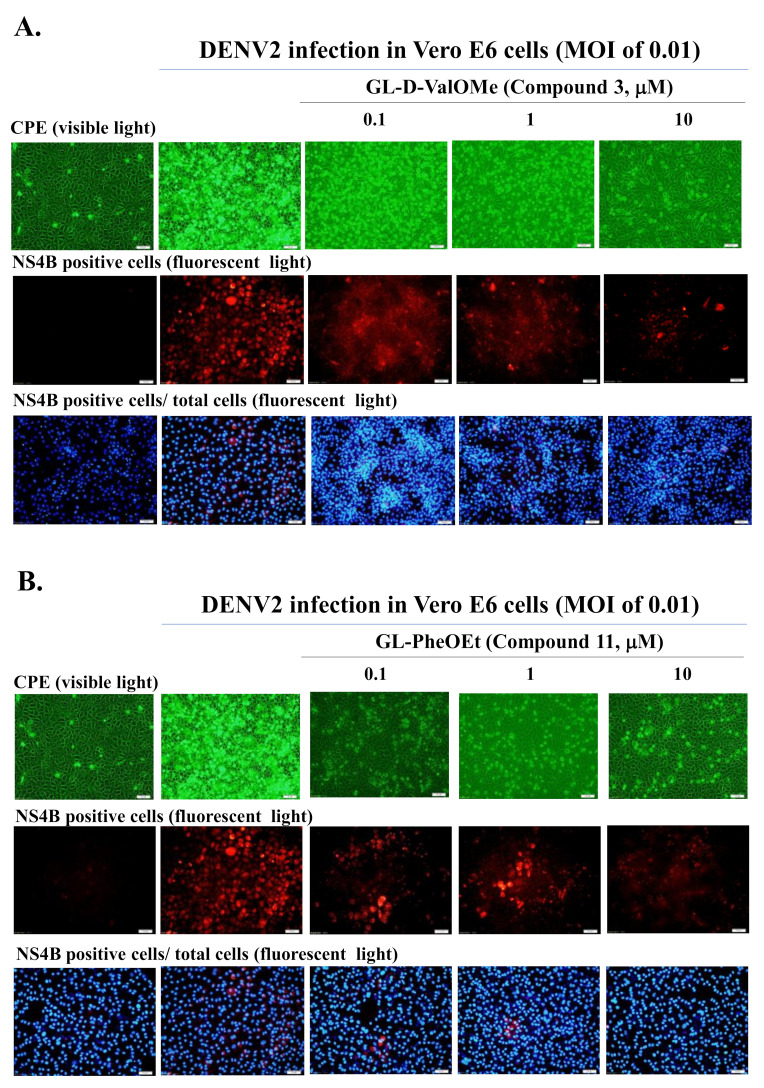
Inhibition of DENV2-induced cytopathic effects and DENV2 protein expression by GL-D-ValOMe (Compound **3**, (**A**) and GL-PheOEt (Compound **11**, (**B**) in Vero E6 cells. Cells were infected with DENV2 at an MOI of 0.01 and immediately treated with the indicated concentrations of Compounds **3** and **11**. Images of DENV2-induced cytopathic effect were photographed 96 h post-infection by light microscopy (**top**). Treated/infected Vero E6 cells were also analyzed using immunofluorescence staining with anti-DENV2 NS4B antibodies. DENV2 infectivity was discovered by the ratio of DENV2 NS4B positive cells (**middle**) to total cells stained with DAPI (**bottom**). Scale bar = 100 µm.

**Figure 3:**
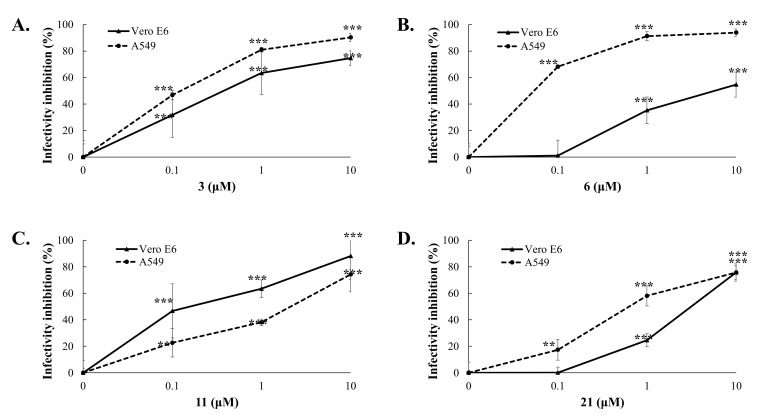
Infectivity inhibition of DENV2 in Vero E6 and A549 cells by active GL conjugates. Cells were infected with DENV2 in the presence and absence of Compounds **3** (**A**), **6** (**B**), **11** (**C**), and **21** (**D**) at concentrations of 0.1, 1, and 10 μM, respectively. Treated/infected cells were also analyzed using immunofluorescence staining with anti-DENV2 NS4B antibodies. Residual infectivity was discovered by the ratio of DENV2 NS4B positive cells to total cells stained with DAPI, and relative inhibition activity was determined based on full infectivity with the subtraction of its residual infectivity. ** *p* value < 0.01; *** *p* value < 0.001 compared with untreated infected cells.

**Figure 4:**
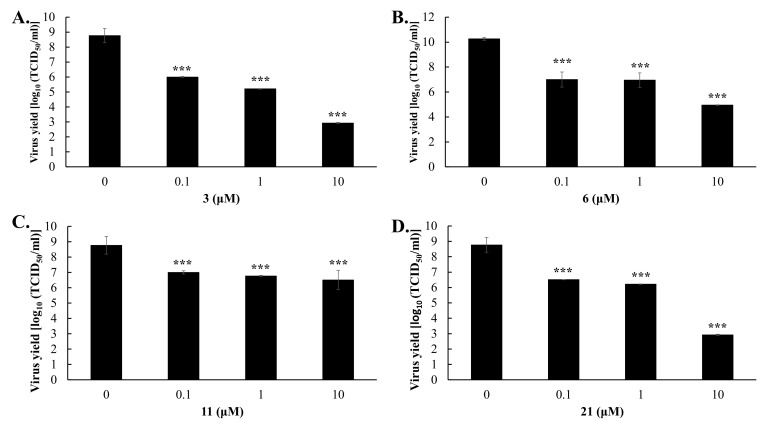
Reduction of DENV2 yield in Vero E6 cells by Compounds **3, 6, 11,** and **21.** Vero E6 cells were infected with DENV2 in the presence and absence of Compounds **3** (**A**), **6** (**B**), **11** (**C**), and **21** (**D**) at concentrations of 0.1, 1, and 10 μM, respectively. Virus yield in the cultured media of treated/infected Vero E6 cells was analyzed 96 h post-treatment by TCID50 assay. *** *p* value < 0.001 compared with untreated infected cells.

**Figure 5:**
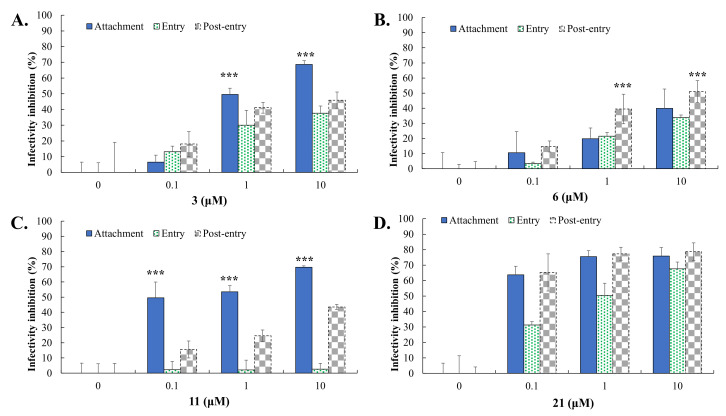
Time-of-addition and removal assay for analyzing antiviral action of compounds **3** (**A**)**, 6** (**B**)**, 11** (**C**)**,** and **21** (**D**) in Vero E6 cells. In the attachment mode, cells were pre-cooled and incubated with fresh-prepared mixtures of active compounds with DENV2 at 4 °C for 1 h. After the removal of the mixtures, the cells were kept at 37 °C for 96 h (a cycle of viral replication) and then were performed by immunofluorescent assay plus DAPI staining. In entry and post-entry modes, cells were infected with DENV2 and treated with the compounds simultaneously (the entry stage) or 1-h post-infection (post-entry stage). After a 1 h incubation period, the virus/compound mixture was removed; the cell monolayer was washed with PBS and cultured for 96 h and then subjected to immunofluorescence staining and DAPI. Finally, the inhibition percentage of DENV2 infectivity was calculated as follows: 1-(percentage of NS4B positivity in treated cells/percentage of NS4B positivity in mock-treated cells). *** *p* < 0.001 compared with the other two antiviral action modes.

In the antiviral screening assay, GL derivatives (Compounds **2**, **3, 4, 6, 7, 8, 9, 11, 15, 16, 18, 21**) (10 μM) were tested firstly as inhibitors to reduce the relative level of DENV2-induced cytopathic effect (CPE) in Vero E6 cells (Table 1). A number of tested compounds showed high (++++) or pronounced (+++) CPE inhibitory activity. The highest inhibitory effect of 75%-100% CPE (++++) in infected cells was exerted by GL conjugates with a D-valine methyl ester (GL-D-ValOMe) **3**, L-tyrosine methyl ester (GL-TyrOMe) **6** (Group I), L-phenylalanine ethyl ester (GL-PheOEt) **11** (Group II), and *ε*-N-carbobenzoxy L-lysine (GL-Lys(Z)OH) **21** (Group III). Data on active compounds are presented in Table 1 and in Figure 2, Figure 3, Figure 4 and Figure 5. Compounds **9**, **16** and **18** had a moderate activity (+++) in inhibiting the CPE of DENV2. Others were not active.

### 2.3. Antiviral Activity of GL Derivatives in DENV Infectivity Assay

A quantitative analysis of GL derivatives’ inhibitory effect against DENV2 was carried out in vitro in infectivity test by immunofluorescence method using anti-Dengue antibodies NS4B (NS4B is a non-structural protein of the 4B virus) according to [45]. It was found that GL conjugates **3, 6, 11,** and **21**, which showed a high CPE inhibition, also significantly reduced the percentage of NS4B-positive cells (91–98%) at a concentration of 10 μM and surpassed GL in this activity (70.5%), which indicates the ability of these compounds to inhibit DENV2 infectivity in Vero E6 cells (Table 1). The active GL derivatives were further selected for studies of cytotoxicity and antiviral potency against DENV1 and DENV2.

### 2.4. Cytotoxicity and Antiviral Potency of Active GL Conjugates

To quantify the antiviral effect and determine the values of 50% inhibitory concentrations (IC50), a quantitative analysis of cytotoxicity and inhibitory effects was carried out. The cytotoxicity and anti-DENV potency of Compounds **3**, **6**, **11**, and **21** were determined by using MTT, cytopathic reduction, and infectivity inhibition assays (Table 1). The MTT cytotoxicity test revealed that the 50% cytotoxic concentration (CC50) of active compounds exceeded 100 μM in Vero E6 cells and A549 cells (Table 1). Meanwhile, the antiviral potency of active GL conjugates **3, 6, 9, 11,** and **21** against DENV2 was estimated in Vero E6 cells. The tested assay indicated that these GL derivatives had an obviously concentration-dependent inhibition of DENV2 infectivity via reducing the viral protein expression in Vero E6 and A549 cells (Table 1, Figure 2 and Figure 3, Supplemental Figure S24). The 50% inhibitory concentration (IC50) values of Compounds **3** and **11** against the DENV2 infectivity were in the range of 0.17–0.50 µM, which were lower than for Compounds **6** (IC50 of 6.0 µM), **21** (IC50 of 2.7 µM), and GL (IC50 of 8.1 µM) in Vero E6 cells (Table 1, Figure 2 and Figure 3). Similarly, the IC50 values of Compounds **3**, **6**, **11**, and **21** are ranging from <0.1–1.56 μM against DENV2 in A549 cells (Table 1, Figure 3). Moreover, the selectivity index (SI=CC50/IC50=552.5) of Compounds **3** and **11** exceeded 60 in both types of cell lines, and SI values of Compounds **6** and 2**1** were >16.7, and >33.7 in Vero E6 cells and >1000, and >103 in A549 cells, respectively. Active GL compounds (hits) **3, 6, 11,** and **21** were also tested as DENV1 inhibitors in Vero E6 cells. It was found that the IC50 values of Compounds **3**, **6**, **11**, and **21** were 2.81 ± 0.54, <0.1, 1.25 ± 0.167, and <0.1 μM, respectively, in the DENV1 infectivity inhibition assay (Supplemental Figure S25). These results indicated that active GL derivatives have significant antiviral effectiveness against DENV1 and DENV2 in a cell-type independent manner, as potential lead compounds to develop antiviral agents against DENV.

### 2.5. Virus Yield Reduction and Attachment Inhibition by Active GL Derivatives

To examine the inhibitory effect of these four active GL derivatives on virus yield, virus titers in the cultured media of treated/infected cells were determined using the TCID50 assay (Figure 4). Compound hits **3**, **6**, **11**, and **21** exhibited a concentration-dependent inhibition on the viral yield of DENV2 in Vero E6 cells, with IC50 values of 0.50 μM for GL-D-ValOMe **3**, 6.0 μM for GL-TyrOMe **6**, 0.2 μM for GL-PheOEt **11**, and 2.7 μM for GL-LysOMe **21**, respectively. The most active inhibitors of DENV2 yield were GL-D-ValOMe **3** and GL-PheOEt **11**.

Time-of-addition/removal assays were further performed to analyze the antiviral action of GL conjugates **3**, **6**, **11**, and **21** by three modes including attachment, entry, and post-entry (Figure 5). GL conjugates **3** and **11** mostly affected the virus binding stage to the cell surface in the attachment mode, with IC50 values of 1.86 μM for the GL conjugate with D-ValOMe **3** and 0.18 μM for the GL conjugate with PheOEt **11**, compared to entry and post-entry modes. Meanwhile, GL derivatives **6** and **21** predominantly impeded the post-entry stage of DENV replication 1 h post-infection (Figure 5). These results indicated that GL conjugates with D-ValOMe **3,** and PheOEt **11** had a more pronounced inhibitory effect on the virus replication at the virus attachment step.

### 2.6. Target Identification of Active GL Derivative Hits 3 and 11 against DENV

**Figure 6:**
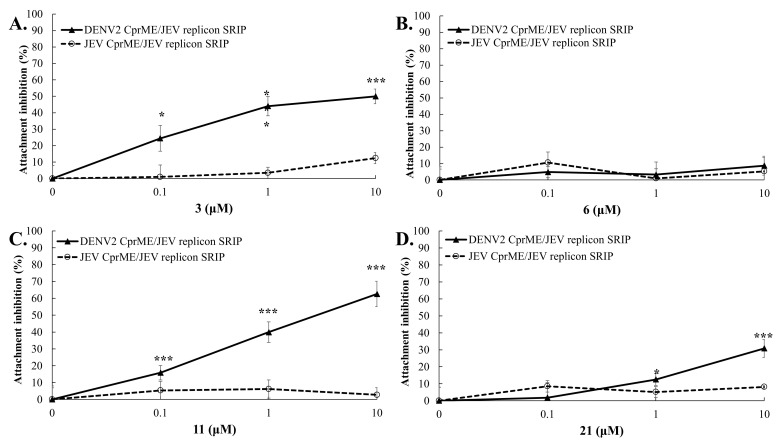
Attachment inhibition of chimeric DENV2 CprM-E/JEV and wild-type JEV SRIPs by active GL conjugates. Vero E6 cells were pre-cooled and incubated with freshly prepared mixtures of DENV2 CprM-E/JEV or wild-type JEV SRIPs, with active GL derivatives 3 (**A**), 6 (**B**), 11 (**C**), and 21 (**D**) at 4 °C for 1 h, respectively. After the removal of the mixtures, the cells were kept at 37 °C for 18 h and then an immunofluorescent assay with anti-JEV NS5 antibodies plus DAPI staining was performed. Finally, the inhibition percentage of SRIP attachment infectivity was measured as follows: 1-(percentage of JEV NS5 positivity in treated cells/percentage of JEV NS5 positivity in mock-treated cells). * *p* < 0.05; *** *p* < 0.001 compared with untreated, infected cells.

**Figure 7:**
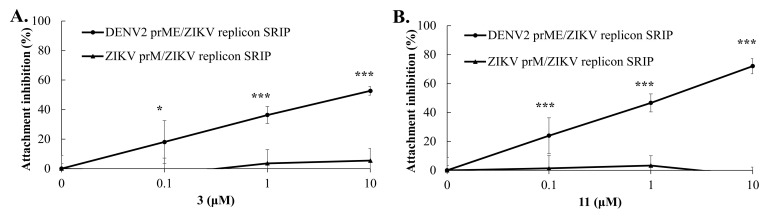
Attachment inhibition of chimeric DENV2 prM-E/ZIKV and wild-type ZIKV SRIPs by active GL derivatives **3** and **11**. Vero E6 cells were pre-cooled and incubated with fresh-prepared mixtures of DENV2 prM-E/ZIKV or wild-type ZIKV SRIPs, with active GL derivatives 3 (**A**), and 11 (**B**) at 4 °C for 1 h, respectively. After the removal of the mixtures, the cells were kept at 37 °C for 18 h and then an immunofluorescent assay with anti-ZIKV NS1 antibodies plus DAPI staining was performed. Finally, the inhibition percentage of SRIP attachment infectivity was measured as follows: 1-(percentage of ZIKV NS1 positivity in treated cells/ percentage of ZIKV NS1 positivity in mock-treated cells). * *p* < 0.05; *** *p* < 0.001 compared with untreated, infected cells.

Since the DENV E protein is primarily responsible for virus attachment, entry to the cells and fusion [13,52], chimeric DENV2 CprME/JEV replicon single-round infectious particles (SRIPs) produced in DENV2 C-prM-E protein-expressing cells transfected with JEV replicon, as well as chimeric DENV2 prM-E/ZIKV replicon SRIPs produced in DENV2 prM-E protein-expressing cells transfected with ZIKV replicon were exploited for identifying the target of active GL conjugates 3 and 11 (Figure 6 and Figure 7). The attachment inhibition assay revealed that GL conjugates **3** and **11** significantly reduced the attachment of chimeric DENV2 CprME/JEV SRIPs and DENV2 prME/ZIKV SRIPs in a concentration-dependent manner but did not impede wild-type JEV and ZIKV SRIPs to bind onto the monolayer of Vero E6 cells (Figure 6 and Figure 7). The GL conjugate 11 had a stronger inhibition effect on E-mediated attachment with IC50 values of 2.82 μM against DENV2 CprME/JEV SRIPs and 1.26 μM against DENV2 prME/ZIKV SRIPs, compared to GL conjugate **3** (IC50 values of 6.69 μM against DENV2 CprME/JEV SRIPs, and 2.82 μM against DENV2 prME/ZIKV SRIPs). The result was the consistency of presumed inhibitory action on DENV2 E protein-mediated attachment by GL conjugates **3** and **11**.

**Figure 8:**
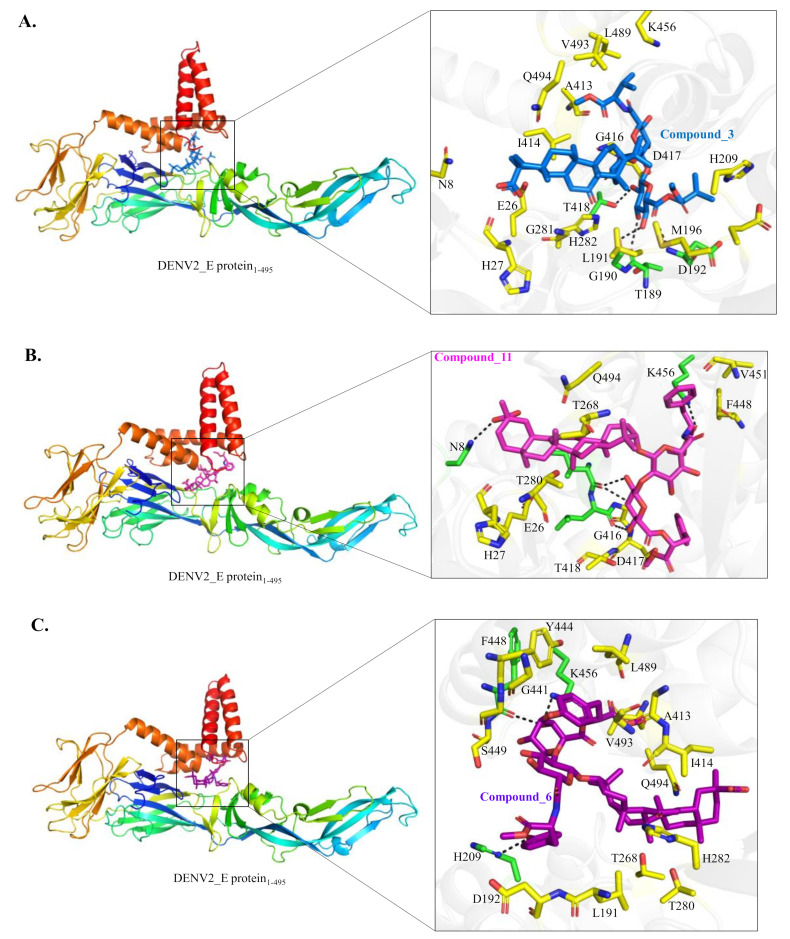
Molecular docking of Compounds **3, 6** and **11** into the β-OG binding pocket of DENV-2 E protein. The best docking pose of Compound **3** (**A**), Compound **11** (**B**), Compound **6** (**C**) to DENV-2 E protein (residues 1-495, PDB: 7KV8). Left, the docked protein-compound model; right, the enlarged view of the β-OG binding pocket. DENV-2 E protein is presented in cartoon and the compounds and interacting residues involved are shown in sticks (green for H-bond interaction; yellow for hydrophobic interaction) and labeled.

The process of molecular docking of the binding site of the DENV2 E protein identified by target-specific ligands was subsequently performed to display possible molecular mechanisms of the antiviral action by GL conjugates **3** and **11** compared to Compound **6**. All of them were found to be docked into the n-octyl-β-d-glucoside (β-OG) binding pocket of the DENV-2 E protein [53]. The detailed interaction between these three compounds and the DENV-2 E protein were shown in Figure 8A–C. Compound **3** forms four hydrogen bonds with a sidechain of T418 (2.9 Å) and mainchain oxygen of T189 (3.2 Å), G190 (2.7 Å) and mainchain amide of D192 (3.3 Å) of the DENV-2 E protein. The hydrophobic interaction between Compound **3** and the DENV-2 E protein involves the following residues N8, E26, H27 (Domain I), L191, E195, M196, H209, G281, H282 (Domain II), A413, I414, G416, D417, K456, L489, V493 and Q494 (stem region) (Figure 8A). Compound **11** forms five hydrogen bonds with a sidechain of N8 (3.0 Å), K456 (2.7 Å) and mainchain oxygen of I414 (2.7 Å/2.9 Å) and L415 (3.2 Å) of the DENV-2 E protein. Compound **11** forms hydrophobic interactions with E26, H27 (Domian I), G266, T268, T280 (Domain II), G416, D417, T418, G445, F448 and V451 (stem region) of the DENV-2 E protein (Figure 8B). Compound **6** forms three hydrogen bonds with a sidechain of H209 (2.9 Å), K456 (2.8 Å) and the mainchain oxygen of F448 (2.7 Å) of the DENV-2 E protein. Compound **6** makes the extensive hydrophobic interaction with L191, D192, T268, H282 (Domain II), A413, I414, G416, G441, Y444, G445, S449, L489, V493 and Q494 (stem region) (Figure 8C). Molecular docking results hinted that Compounds **3** and **11** might pose a similar interaction pattern with the DENV2 E protein, in which Compounds **3** and **11**, but not **6**, could form hydrogen bonds and hydrophobic interactions with N8, E26, H27, D417, T418 of the hydrophobic packet in the hinge region between domains I and II of the E protein, which was occupied by the E protein inhibitor n-octyl-β-D-glucoside (BOG).

## 3. Discussion

In our previous papers [40,42,45,46] we showed that the introduction of amino acid residues in the GL molecule greatly influenced its antiviral activity including anti-DENV2 [45], and the presence of a free C30-carboxy group in the triterpene part is important for the enhancing antiviral effects of GL derivatives. Therefore, in this study, only GL conjugates with amino acid residues with an unchanged triterpene part of the molecule were synthesized and studied in vitro as DENV1 and DENV2 inhibitors. It is important that we received some GL conjugates containing amino acid residues included in the active binding sites of the flavivirus’s target proteins such as E-protein, NS2-NS3-proteases, NS3-helicase, and RdRp (Val, Leu, Met, Phe, Tyr, Lys, etc.) [16,18,19,20,21,54].

Among the group I GL derivatives, conjugates with methyl esters of L- or D-amino acids **2**–**8**, which were tested as DENV2 inhibitors in Vero E6 cells, only two compounds were found (**3** and **6)** as high active inhibitors of DENV2 CPE and infectivity (Table 1). Conjugate **6** containing two TyrOMe fragments showed significant inhibition of in vitro replication of DENV2 and its IC50 (5.98 μM) was less than for GL. The analog **7**, conjugated with D-TyrOMe, was less active as 25~50% CPE inhibition, and 51.7% inhibition of NS4B-positive cells at 10 μM concentration (Table 1), but was close to that of Compound **6**. The most active compound among this group of GL derivatives was GL-D-ValOMe **3** (IC50 of 0.12–2.81 μM in virus infectivity and 0.50 μM in virus yield, SI > 201) (Table 1, Figure 2, Figure 3 and Figure 4, Supplemental Figures S24 and S25), thus it was chosen as the first hit compound for further studies. Despite less activity in the virus infectivity assay, conjugate **6** was also an active compound in the virus yield reduction assay (IC50 of 2.68 μM), and thus it was chosen as the second hit compound for the advanced studies of antiviral activity in vitro.

Conjugates **9**–**16** of group II containing ethyl esters of amino acids are also promising for the development of new DENV2 inhibitors. Among the tested compounds, two hit compounds were found, conjugate GL-PheOEt **11** was the most active compound to inhibit DENV infectivity (IC50 of 0.18–1.56 μM, SI > 64) and virus yield reduction (IC50 of 0.17 μM) (Table 1, Figure 2, Figure 3 and Figure 4, Supplemental Figures S24 and S25). Compound **11** was of interest as a third hit molecule for advanced studies in vitro.

GL derivatives of the group III **17**–**21** containing free amino acids residues lost the antiviral activity against DENV2. Only conjugate **21** containing the long-chaired diamino acid ϵ-Z-LysOH residues with the protected ε-NH_2_ showed a pronounced antiviral activity (IC50 of <0.1–2.68 μM in DENV infectivity) and exceeded GL as a DENV2 inhibitor in Vero E6 cells, therefore it was used for further antiviral studies too. However, as is shown in Table 1, the elongation of the amino acid residues by introducing dipeptide Gly-PheOEt (Compound **16**) does not potentiate the antiviral activity of the GL conjugate. The introduction of amino acid with β-position of NH_2_ group (Compound **15**) resulted in a similar effect. Therefore, the presence of the CONH bond formed with the α-NH_2_ group of amino acids is important for the exhibition of antiviral effects and improvement of anti-DENV potency of GL derivatives. It should also be noted that the conjugation of GL with methyl/ethyl esters of amino acids plays a decisive role in enhancing the antiviral activity of GL derivatives.

As a result of the antiviral activity study, three hit compounds were discovered to be GL conjugates with methyl and ethyl esters of the aromatic amino acids (Compounds **6** and **11**), and D-valine methyl ester (Compound **3**) with free COOH group in the triterpene part as compounds with the significantly improved antiviral activity against DENV2. Compounds **3**, **6**, and **11** exhibit high antiviral potency regardless of the cell type against DENV1 and DENV 2 in Vero E6 and A549 cells. Earlier, we discovered Compound **6** as a highly active Zika virus inhibitor [46]. GL conjugates **3** and **11** mostly affected the virus binding stage to the cell surface in the attachment mode of the time-of-addition assay (Figure 5). GL conjugate **6** predominantly impeded the post-entry stage of DENV2 replication. Thus, GL conjugates with D-ValOMe **3** and PheOEt **11** had a more pronounced inhibitory effect on the virus replication at the virus attachment stage but might show a distinct interaction with the DENV E protein.

The E protein-mediated attachment assay with chimeric DENV2/JEV and DENV2/ZIKV SRIPs revealed that GL conjugates **3** and **11** significantly reduced the attachment of DENV2 CprME/JEV and DENV2 prME/ZIKV SRIPs but not wild-type JEV and ZIKV SRIPs in Vero E6 cells in a concentration-dependent manner (Figure 6 and Figure 7), confirming the putative inhibitory effect on DENV E-mediated attachment of Compounds **3** and **11.** Among the discovered GL hit derivatives, Compounds **3** and **11** might be chosen as the lead molecules perspective for the development of DENV inhibitors due to the simple synthetic procedure for their preparation and high inhibitory activity against DENV2. The DENV E protein is a key protein of the flavivirus life cycle, playing an important role in virus attachment to the host cell, entry and fusion [16,18,52]. It contains three ectodomains and a stem anchor to provide a link to the viral membrane [54]. The crystal structure of the DENV E protein was opened in 2003 [53] and it was reported that a hydrophobic site between domains I and II of the E protein binds to the n-octyl-β-D-glucose (β-OG) molecule. The cavity in the E protein fitting β-OG was designated as the β-OG pocket, which is used for in silico screening of DENV inhibitors [55,56] and searching drug-like compounds including natural substances with a high antiviral potency against DENV [20,25,54,57]. Interestingly, Compounds **3** and **11** had hydrogen bonds and hydrophobic interactions with the hydrophobic pocket formed by domains I and II, and the stem region of the DENV E protein, whose ligand binding pocket was similar to the E protein inhibitor BOG binding site in the hinge region between domains I and II of the E protein.

## 4. Materials and Methods

### 4.1. General Experimental Procedures for Chemical Synthesis

IR spectra were recorded on the Prestige-21 spectrophotometer (Shimadzu, Kyoto, Japan). NMR spectra were measured on a Brüker AVANCE-III pulse spectrometer (Bruker, Ettlingen, Germany), operating at 500 MHz (^1^H) and 125 MHz (^13^C) in CD_3_OD or DMSO-d_6_ using tetramethylsilane as the internal standard. Chemical shifts are given in δ (ppm) and the coupling constants (*J*) are given in hertz (Hz). Optical rotations were measured with a Perkin-Elmer 341 MC digital polarimeter (PerkinElmer Inc., Wellesley, MA, USA) with a sodium lamp (D-line wavelength = 589 nm). Thin-layer chromatography was carried out on the Sorbfil plates (Sorbfil, Sorb Polymer, Krasnodar, Russia) using benzene-ethanol or chloroform-ethanol mixtures (5:1, v%). Spots were detected by 5% H_2_SO_4_ solution in ethanol with subsequent heating at 180–200 °C for 2–3 min. GL derivatives were isolated in an analytically pure state by column chromatography on Silica gel KSK SG (50–150 mesh, Sorbfil) with a TLC control. The purity of all GL derivatives for biological testing was >95%, as determined by HPLC analysis. HPLC data for GL derivatives are given in Supplemental Figures S26–S45. The HPLC analysis was carried out on a Shimadzu LC-20 (Shimadzu) liquid chromatograph with an absorbance detector at 254 nm using an Atlantis C18 (250 × 4.6 mm, 5 μM) (Waters, Dublin, Irland), Zorbax RX C18 (250 × 4.6 mm, 5 μM) (Agilent, Torrance, CA, USA), Discovery C18 (250 × 3.0 mm, 5 μM) (GRACE, San Jose, CA, USA) or Vydac 218TP C18 (250 × 4.6 μM) (PELCO, Bellefonte, PA, USA) columns. The mobile phase was CH_3_OH, with a flow rate of 1 mL/min. Detection was carried out at a wavelength of 254 nm. Electronic absorption spectra were recorded in real-time at the moment the eluent passed through the detector cell.

### 4.2. Chemicals

All chemicals were used as commercially available. *N*-hydroxysuccinimide, *N*,*N*’-diciclohexylcarbodiimide, 1-ethyl-3-(3-dimethylaminopropyl)carbodiimide hydrochloride, triethyl amine were purchased from ACROS organics and Sigma-Aldrich (St. Louis, MI, USA). L- and D-amino acids, glycyl-L-phenylalanine, and methyl or ethyl esters of amino acids hydrochlorides were purchased from Sigma-Aldrich or Alfa Eaesar Co. (Heysham, England). The solvents for chromatography were purified by standard procedures. GL was produced from a commercial mono ammonium salt according to [58] and had a purity of 96.5 ± 0.5% according to HPLC.

### 4.3. A General Procedure to Synthesize Compounds **2**–**5**

To a solution of GL (1 mmol) in dimethylformamide (20 mL), amino acid methyl ether hydrochloride (2.5–3.0 mmol), 1-ethyl-3(3-dimethylaminopropyl)carbodiimide hydrochloride (2.5–3.0 mmol), and triethylamine (8.0–10.0 mmol) were added. A reaction mixture was mixed at room temperature for 24 h, diluted with cold water (100 mL), and acidified with citric acid (pH 3–4). A precipitate was filtered off, washed with water, dried and purified by column chromatography on Silica gel, eluting with a gradient mixture of chloroform-ethanol (400:10→50:10, v%) or with chloroform-methanol-water (400:10:1→50:10:1 (*v*/*v*) mixtures with TLC control.

### 4.4. A General Procedure to Synthesize Compounds **6**–**16**

To a solution of GL (1 mmol) in dimethylformamide (20 mL), *N*-hydroxysuccinimide (5.0–5.2 mmol) and *N*,*N*′-dicyclohexylcarbodiimide (2.3–2.5 mmol) were added, and a mixture was stirred at room temperature for 5 h. The precipitate of formed *N*,*N*′-dicyclohexylurea was filtered off. To filtrate amino acids, methyl/ethyl esters hydrochlorides (2.5 mmol) and triethylamine (8.0–10.0 mmol) were added, and a reaction mixture was stirred for 20–22 h at room temperature. Then, it was diluted with cold water and acidified with citric acid (pH 3–4). A precipitate was filtered off, washed with water, dried and purified by column chromatography as above.

### 4.5. A General Procedure to Synthesize Compounds **17**–**21**

To a solution of GL (1 mmol) in dimethylformamide (20 mL), *N*-hydroxysuccinimide (5.0–5.2 mmol) and *N*,*N*′-dicyclohexylcarbodiimide (2.5 mmol) were added, and a mixture was stirred at 20–22 °C for 5 h. The precipitate of *N*,*N*′-dicyclohexylurea was filtered off, and a filtrate (solution A) was used further in the reaction with amino acids. An amino acid solution (2.5–3.0 mmol) (solution B) was prepared in a mixture of 1N aqueous NaOH (10 mL) and DMF (10 mL) at 0–+5 °C. Solution B was gradually added to solution A under stirring and cooling in the ice bath, and then a reaction mixture was kept for 24 h at room temperature with stirring. A mixture was diluted with cold water, acidified with 5% hydrochloric acid to pH 2–3, the precipitate was filtered off, washed with water, and dried. Analytically pure samples were produced by column chromatography as above.

### 4.6. 3-O-{2-O-[N-(β-D-Glucopyranosyluronoyl)-L-valine methyl ester]-N-(β-D-glucopyranosyluronoyl)-L-valine methyl ester}-(3β, 20β)-11-oxo-30-norolean-12-ene-30-oic Acid (**2**) [42]

An amorphous solid, yield 55%. [α]_D_^20^ + 55° (*c* 0.04; EtOH). Lit. [42]: [α]*_D_*^20^ +52° (*c* 0.04, EtOH). HPLC (97.8 ± 0.8%, τ 2.77 min) (Supplemental Figure S26). IR (ν, cm^−1^): 3600–3200 (OH, NH), 1738 (COOMe), 1661 (C^11^=O), 1531 (CONH). ^1^H NMR (CD_3_OD, *δ*): 7.88 (2H, s, 2NH), 5.57 (1H, s, H12), 4.74 (1H, d, *J* = 7.7, H1″), 4.58 (1H, d, *J* = 7.4, H1′), 4.44-4.42 (2H, m, H3″, H3′), 3.84-3.80 (3H, m, H4″, H4′, H3), 3.74 (6H, c, 2OCH_3_), 3.66-3.61 (3H, m); 3.54-3.45 (4H, m), 3.35-3.31 (3H, m), 2.86 (1H, c, H18), 2.68-2.52 (3H, m), 2.42 (1H, c, H9), 2.21-2.14 (5H, m), 1.94-1.58 (7H, m), 1.45 (1H, s), 1.40 (3H, s, CH_3_), 1.36 (2H, s), 1.27-1.24 (2H, m), 1.16 (2H, s), 1.13 (6H. s, 2CH_3_), 1.05 (3H, s, CH_3_), 1.00 (3H, s, CH_3_), 0.98-0.92 (15H, m, 5CH_3_), 0.84 (1H, m), 0.82 (3H, s, CH_3_), 0.78-0.74 (2H, m). ^13^C NMR (CD_3_OD, *δ*): 202.25 (C11), 180.16 (C30), 171.67 (C6″), 171.37 (C6′), 170.97 (C13), 128.85 (C12), 105.29 (C1″), 104.80 (C1′), 90.37 (C3), 82.11 (C2′), 77.50 (C5″), 77.23 (C5′), 76.44 (C3″), 75.97 (C3′), 75.35 (C2″), 73.17 (C4″), 73.05 (C4′), 62.96 (C9), 56.32 (C5), 49.74 (C18), 46.63 (C8), 44.77 (C20), 44.49 (C14), 42.30 (C19), 40.55 (C4), 40.14 (C1), 38.91 (C22), 37.97 (C10), 33.71 (C7), 32.87 (C17), 32.23 (C21), 29.19 (C29), 28.75 (C23), 28.41 (C28), 27.51 (C16), 27.39 (C15), 27.30 (C2), 23.88 (C27), 18.26 (C26), 19.32 (C6), 18.74 (C24), 18.11 (C25); 2ValOMe: 172.99 (COOMe), 172.87 (COOMe), 58.69 (α-CH), 58.28 (α-CH), 52.71 (OCH_3_), 52.58 (OCH_3_), 31.98 (2CH), 19.50 (CH_3_), 19.40 (CH_3_), 17.05 (CH_3_), 16.99 (CH_3_). Anal. calcd. for C_54_H_84_N_2_O_18_ C 61.81, H 8.07, N 2.67 %; found, C 61.73, H 7.95, N 2.58. M = 1049.22.

### 4.7. 3-O-{2-O-[N-(β-D-Glucopyranosyluronoyl)-D-valine methyl ester]-N-(β-D-glucopyranosyluronoyl)-D-valine methyl ester}-(3β, 20β)-11-oxo-30-noroleane-12-ene-30-oic Acid (**3**) [43]

An amorphous solid, yield 53%; [α]*_D_*^20^ +60° (*c* 0.06, EtOH). Lit. [43]: [α]*_D_*^20^ +65° (*c* 0.06, EtOH). HPLC (96.8 ± 0.8%, τ 2.86 min) (Supplemental Figure S27). IR (ν, cm^−1^): 3600-3200 (OH, NH), 1739 (COOMe), 1661 (C^11^=O), 1533 (CONH). ^1^H NMR (CD_3_OD, *δ*): 7.90 (1H, s, NH), 7.33 (1H, s, 1NH), 5.57 (1H, c, H12), 4.70 (1H, d, *J* = 7.7, H1′’), 4.62 (1H, d, *J* = 7.5, H1′), 4.43-4.42 (2H, m, H3′’, H3′), 3.83-3.76 (3H, m, H4′’, H4′, H3), 3.74 (6H, c, 2OCH_3_), 3.65-3.60 (2H, m), 3.57-3.49 (4H, м), 3.44-3.40 (1H, m), 3.34-3.31 (3H, m), 3.22-3.19 (1H, m), 3.15-3.13 (1H, m), 2.86 (1H, c, H18), 2.69-2.67 (2H, m), 2.42 (1H, s, H9), 2.19-2.11 (5H, m), 1.93-1.53 (7H, m), 1.40 (6H, s, 2CH_3_), 1.36 (1H, s), 1.30-1.20 (2H, m), 1.16 (3H, s, CH_3_), 1.12 (6H, s, 2CH_3_), 1.06 (3H, s, CH_3_), 1.03-0.99 (2H, m), 0.95 (3H, s, CH_3_), 0.94 (3H, s, CH_3_), 0.92 (3H, s, CH_3_), 0.84 (3H, s, CH_3_), 0.82 (3H, s, CH_3_), 0.80-0.75 (2H, m). ^13^C NMR (CD_3_OD, *δ*): 201.19 (C11), 179.00 (C30), 170.80 (C13), 170.20 (C1′’), 170.13 (C6′), 127.52 (C12), 104.85 (C1′’), 103.60 (C1′), 89.31 (C3), 82.34 (C2′), 76.25 (C5′’), 75.95 (C5′), 75.75 (C3′’), 74.89 (C3′), 74.25 (C2′’), 71.67 (C4′’,C4′), 61.72 (C9), 55.14 (C5), 48.49 (C18), 45.37 (C8), 43.52 (C20), 43.21 (C14), 40.98 (C19), 39.22 (C4), 38.84 (C1), 37.64 (C22), 36.66 (C10), 32.41 (C7), 31.60 (C17, C21), 27.89 (C29), 27.45 (C28), 26.93 (C23), 26.21 (C16), 26.08 (C15), 25.99 C2), 22.25 (C27), 18.13 (C26), 17.96 (C6), 17.06 (C25), 16.85 (C24); 2D-ValOMe: 171.80 (COOMe), 171.51 (COOMe), 57.13 (2α-CH), 51.42 (2OCH_3_), 30.68 (CH), 30.63 (CH), 18.15 (CH_3_), 18.13 (CH_3_), 15.72 (CH_3_), 15.53 (CH_3_). Anal. calcd. for C_54_H_84_N_2_O_18_ C 61.81, H 8.07, N 2.67 %; found, C 61.67, H 7.88, N 2.55%. M = 1049.22.

### 4.8. 3-O-{2-O-[N-(β-D-Glucopyranosyluronoyl)-L-isoleucine methyl ester]-N-(β-D-glucopyranosyluronoyl)-L-isoleucine methyl ester}-(3β, 20β)-11-oxo-30-norolean-12-ene-30-oic Acid (**4**)

An amorphous solid, yield 54%; [α]_D_^20^ + 58° (*c* 0.04, MeOH). HPLC (98.2 ± 0.8%, τ 2.88 min) (Supplemental Figure S28). IR (ν, cм^−1^): 3500-3200 (OH, NH), 1739 (COOMe), 1661 (C=O), 1532 (CONH). ^1^H NMR (CD_3_OD, δ): 5.58 (1H, s, H12), 4.58 (1H, d, *J* = 7.4, H1″), 4.48 (1H, d, *J* = 7.4, H1′), 4.45-4.42 (2H, m, H3′’, H3′), 3.82-3.77 (3H, m, H4′’, H4′, H3), 3.72 (3H, s, OCH_3_), 3.70 (3H, s, OCH_3_), 3.69-3.58 (6H, m), 3.54-3.42 (5H, m), 3.32-3.22 (6H, m), 2.73 (1H, m, H18), 2.45 (1H, s, H9), 2.21-2.10 (2H, m), 1.94-1.58 (15H, m), 1.50-1.45 (5H, m), 1.41 (6H, s, 2CH_3_), 1.28-1.20 (2H, m), 1.16 (3H, s, CH_3_), 1.13 (3H, s, CH_3_), 1.11 (3H, s, CH_3_), 1.06 (3H, s, CH_3_), 0.96-0.91 (13H, m, 3CH_3_, 2CH_2_), 0.84, 0.83 (6H, both s, 2CH_3_), 0.80-0.77 (2H, m). ^13^C NMR (CD_3_OD, δ): 201.09 (C11), 179.03 (C30), 171.45 (C13), 169.92 (C6″), 169.86 (C6′), 127.56 (C12), 103.72 (C1″), 103.54 (C1′), 88.96 (C3), 80.27 (C2′), 76.27 (C5″), 75.90 (C5′), 74.94 (C3″), 74.65 (C3′), 74.02 (C2″), 72.02 (C4″), 71.94 (C4′), 61.74 (C9), 55.02 (C5), 48.52 (C18), 45.37 (C14), 43.53 (C20), 43.24 (C8), 41.07 (C19), 39.30 (C4), 38.95 (C1), 37.36 (C22), 36.72 (C10), 32.43 (C7), 31.60 (C17), 30.66 (C21), 27.86 (C29), 27.43 (C28), 27.07 (C23), 26.24 (C23), 26.03 (C16, C15), 22.55 (C27), 17.99 (C26), 17.12 (C25), 14.61 (C24); 2IleOMe: 171.70 (COOEt), 171.63 (COOMe), 56.62 (α-CH), 56.22 (α-CH), 51.39 (OCH_3_), 51.25 (OCH_3_), 37.65 (CH), 37.49 (CH), 25.25 (CH_2_), 24.79 (CH_2_), 15.92 (CH_3_), 15.69 (CH_3_), 10.61 (CH_3_), 10.48 (CH_3_). Anal. calcd. for C_56_H_88_N_2_O_18_ C 62.43, H 8.23, N 2.60%; found, C 62.24, H 8.05, N 2.46%. M = 1077.27.

### 4.9. 3-O-{2-O-[N-(β-D-Glucopyranosyluronoyl)-L-phenylalanine methyl ester]-N-(β-D-glucopyranosyluronoyl)-L-phenylalanine methyl ester}-(3β, 20β)-11-oxo-30-norolean-12-ene-30-oic Acid (**5**) [42]

An amorphous solid, yield 52%. R_f_ 0.52 (бензол-этанол, 5:1); [α]_D_^20^ + 62°C (*c* 0.05, EtOH). Lit. [42]: [α]_D_^20^ + 62°C (*c* 0.04, EtOH). HPLC (97.5 ± 0.8%, τ 4.03 min) (Supplemental Figure S29). IR (ν, cm^−1^): 3600-3200 (OH, NH), 1742 (COOMe), 1645 (C=O), 1551 (CONH), 1497 (Ph). ^1^H NMR (CD_3_OD, *δ*): 7.26-7.08 (10H, m, 2C_6_H_5_), 5.55 (1H, s, H12), 4.77 (1H, d, *J* = 7.7, H1″), 4.72 (2H, d, *J* = 7.5, H1′), 3.75-3.70 (5H, m), 3.68, 3.66 (6H, both s, 2OCH_3_), 3.62-3.57 (4H, m), 3.45-3.35 (3H, m), 3.22-3.18 (3H, m), 3.15-3.04 (5H, m), 2.63-2.05 (5H, m), 1.85-1.52 (8H, m), 1.36 (6H, s, 2CH_3_), 1.22-1.15 (2H, m), 1.12 (3H, s, CH_3_), 1.09 (3H, s, CH_3_), 1.06-1.02 (2H, m), 0.96 (3H, s, CH_3_), 0.88-0.86 (2H, m), 0.80 (3H, s, CH_3_), 0.75 (3H, s, CH_3_), 0.73-0.67 (2H, m). ^13^C NMR (CD_3_OD, *δ*): 201.11 (C11), 179.05 (C30), 171.32 (C13), 169.88 (C6″), 169.75 (C6′), 127.52 (C12), 103.46 (C1″), 103.27 (C1′), 89.29 (C3), 79.56 (C2′), 76.42 (C5″), 75.80 (C5′), 74.50 (C3″), 74.36 (C3′), 73.96 (C2″), 72.06 (C4″), 71.90 (C4′), 61.70 (C9), 55.00 (C5), 48.47 (C18), 45.34 (C8), 43.57 (C20), 43.19 (C14), 41.10 (C19), 39.24 (C4), 38.92 (C1), 37.64 (C22), 37.05 (C10), 32.36 (C7), 31.56 (C17), 30.65 (C21), 27.84 (C29), 27.43 (C28), 27.03 (C23), 26.91 (C2), 26.19 (C16), 25.98 (C15), 22.56 (C27), 17.93 (C26), 17.19 (C6), 17.07 (C25, C24); 2PheOMe: 171.55 (COOMe), 171.32 (COOMe), 136.12 (C Ar), 135.97 (C Ar), 129.07 (2C Ar), 128.94 (2C Ar), 128.83 (C Ar), 128.31 (2C Ar), 128.27 (C Ar), 126.78 (2C Ar), 53.40 (α-CH), 53.03 (α-CH), 51.59 (OCH_3_), 51.46 (OCH_3_), 36.87 (CH_2_), 36.64 (CH_2_). Anal. calcd. for C_62_H_84_O_18_N_2_ C 62.02, H 7.39, N 2.45%, found, C 61.95, H 7.30, N 2.35%. M = 1145.30.

### 4.10. 3-O-{2-O-[N-(β-D-glucopyranosyluronoyl)-L-tyrosine methyl ester]-N-(β-D-glucopyranosyluronoyl)-L-tyrosine methyl ester}-(3β,20β)-11-oxo-30-noroleane-12-ene-30-oic acid (6) [42,46]

An amorphous solid, yield 56%; [α]_D_^20^ + 61° (c 0.08, MeOH). Lit. [46]: [α]_D_^20^ + 60° (c 0.04, MeOH). HPLC (98.0 ± 0.8%, τ 7.95 min) (Supplemental Figure S30). IR (ν, cm^−1^): 3600-3200 (OH, NH), 1734 (COOMe), 1665 (C=O), 1615, 1597, 1517 (Tyr, CONH). ^1^H NMR (CD_3_OD, *δ*): 7.87 (2H, s, 2NH), 6.99 (2H, d, *J* = 8.3, H Ar.), 6.95 (2H, d, *J* = 8.3, H Ar), 6.78 (2H, d, *J* = 8.3, H Ar), 6.68 (2H, d, *J* = 8.3, H Ar), 5.57 (1H, s, H12), 4.81 (1H, d, *J* = 7.7, H1″), 4.69 (2H, m), 4.55 (1H, d, *J* = 7.5, H1′), 3.83-3.73 (2H, m), 3.68, 3.65 (6H, s, 2OCH_3_), 3.65-3.63 (2H, m), 3.50-3.44 (4H, m), 3.30-3.28 (2H, m), 3.14-3.03 (6H, m), 2.96 (1H, s), 2.84 (1H, s, H18), 2.80-2.64 (2H, m), 2.39 (1H, s, H9), 2.20-1.54 (10H, m), 1.38 (6H, br s, 2CH_3_), 1.25-1.16 (2H, m), 1.15 (3H, s, CH_3_), 1.11 (3H, s, CH_3_), 1.08 (3H, s, CH_3_), 1.00 (3H, s, CH_3_), 0.95-0.90 (2H, m), 0.81 (3H, s, CH_3_), 0.77 (3H, s, CH_3_), 0.72-0.68 (2H, m). ^13^C NMR (CD_3_OD, *δ*): 201.20 (C11), 179.02 (C30), 171.41 (C13), 169.94 (C6″), 169.75 (C6′), 127.58 (C12), 103.45 (C1″), 103.19 (C1′), 89.29 (C3), 79.34 (C2′), 76.36 (C5″), 75.78 (C5′), 74.38 (C3″, C3′), 73.89 (C2″), 72.08 (C4″), 71.95 (C4′), 61.73 (C9), 55.05 (C5), 48.44 (C18), 45.38 (C8), 43.54 (C20), 43.19 (C14), 41.04 (C19), 39.30 (C4), 39.02 (C1), 37.67 (C22), 36.66 (C10), 32.38 (C7), 31.58 (C17), 30.64 (C21), 27.93 (C29), 27.50 (C28), 27.15 (C23), 26.22 (C2), 25.99 (C16), 25.83 (C15), 22.71 (C27), 18.00 (C26), 17.06 (C6), 15.99 (C25), 15.80 (C24); 2TyrOMe: 171.58 (COOMe), 171.52 (COOMe), 156.20 (C Ar), 156.13 (C Ar), 130.18 (2C Ar), 130.02 (2C Ar), 126.57 (C Ar), 126.57 (C Ar), 115.20 (2C Ar), 115.08 (2C Ar), 53.59 (α-CH), 53.41 (α-CH), 51.67 (OCH_3_), 51.58 (OCH_3_), 36.23 (2CH_2_). Anal. calcd. for C_62_H_84_N_2_O_20_ C 63.25; H 7.19; N 2.38%; found, C 63.15, H 7.28, N 2.30%. M = 1177.40.

### 4.11. 3-O-{2-O-[N-(β-D-Glucopyranosyluronoyl)-D-tyrosine methyl ester]-N-(β-D-glucopyranosyluronoyl)-D-tyrosine methyl ester}-(3β, 20β)-11-oxo-30-noroleane-12-ene-30-oic Acid (**7**)

An amorpous solid, yield 55%; [α]_D_^20^ + 46° (*c* 0.04, MeOH). HPLC (98.5 ± 0.8%, τ 2.66 min) (Supplemental Figure S31). IR (ν, cm^−1^): 3600-3200 (OH, NH), 1736 (COOCH_3_), 1656 (C=O), 1616, 1594, 1516 (CONH, Tyr). ^1^H NMR (CD_3_OD, *δ*): 7.96, 7.70 (2H, s, 2NH), 7.00 (2H, d, *J* = 7.8, H Ar), 6.96 (2H, d, *J* = 7.8, H Ar), 6.79 (2H, d, *J* = 7.4, H Ar), 6.71 (2H, d, *J* = 7.2, H Ar), 5.61 (1H, br s, H12), 4.72-4.58 (4H, m), 3.77-3.75 (2H, m), 3.68, 3.67 (6H, s, 2OCH_3_), 3.50-3.48 (4H, m), 3.31-3.28 (2H, m), 3.10-3.03 (6H, m), 2.95 (1H, s), 2.83 (1H, s, H18), 2.67-2.57 (3H, m), 2.38-1.56 (10H, m), 1.38 (6H, br s, 2CH_3_), 1.26 (2H, s), 1.16 (2H, s), 1.11 (3H, s, CH_3_), 1.08 (3H, s, CH_3_), 1.01 (3H, s, CH_3_), 0.99 (6H, s, 2CH_3_), 0.84-0.82 (2H, m), 0.77 (3H, s, CH_3_), 0.69 (2H, m). ^13^C NMR (CD_3_OD, *δ*): 202.25 (C11), 180.19 (C30), 170.96 (C13), 171.22 (C6″), 171.16 (C6′), 127.79 (C12), 104.58 (C1″), 104.32 (C1′), 90.45 (C3), 80.53 (C2′), 77.47 (C5″), 76.94 (C5′), 75.56 (C3″), 75.47 (C3′), 74.91 (C2″), 73.17 (C4″, C4′), 62.94 (C9), 56.32 (C5), 49.61 (C18), 46.55 (C8), 44.71 (C20), 44.38 (C14), 42.27 (C19), 40.50 (C4, C1), 37.87 (C22), 36.99 (C10), 33.59 (C7), 32.76 (C17), 31.70 (C21), 29.14 (C29), 28.58 (C28), 28.43 (C23), 27.39 (C16), 27.18 (C15, C2), 23.97 (C27); 19.26 (C26), 18.26 (C6), 17.21 (C25), 17.05 (C24); 2D-TyrOMe: 172.72 (COOMe), 172.62 (COOMe), 157.30, 157.22 (2C Ar), 131.37 (2C Ar), 131.20 (2C Ar); 130.28 (2C Ar), 116.44 (2C Ar), 116.33 (2C Ar), 54.78 (α-CH); 54.63 (α-CH); 52.95 (OCH_3_), 52.86 (OCH_3_), 37.48, 37.40 (2CH_2_). Anal. cacl. for C_62_H_84_N_2_O_20_ C 63.25; H 7.19; N 2.38%; found, C 63.05, H 7.10, N 2.25%. M = 1177.40.

### 4.12. 3-O-{2-O-[N-(β-D-Glucopyranosyluronoyl)-L-methionine methyl ester]-N-(β-D-glucopyranosyluronoyl)-L-methionine methyl ester}-(3β, 20β)-11-oxo-30-noroleane-12-ene-30-oic Acid (**8**) [50]

An amorphous solid, yield 57%, [α]_D_^20^ + 58° (*c* 0.06, MeOH). HPLC (95.8 ± 0.8%, τ 2.65) (Supplemental Figure S32). Lit. [50]: [α]_D_^20^ + 56° (*c* 0.02, MeOH). IR (ν, cm^−1^): 3500-3200 (OH, NH); 1739 (COOH); 1656 (C=O); 1534 (CONH). ^1^H NMR (CD_3_OD, *δ*): 7.84 (1H, br s, NH), 7.36 (1H, br. s, NH), 5.57 (1H, c, H12), 4.77 (1H, d, *J* = 7.4, H1′’), 4.66-4.52 (3H, m, H-1′, H3′’, H3′), 3.87-3.75 (3H, m, H2′’, H2′, H3), 3.75, 3.73 (6H, both s, 2OCH_3_), 3.69-3.41 (6H, m), 3.33-3.30 (1H, m), 2.86 (1H, s, H18), 2.70-2.48 (8H, m), 2.41 (1H, s, H9), 2.18-2.16 (4H, m), 2.09, 2.07 (4H, both s), 2.04-1.60 (12H, m), 1.41 (6H, s, 2CH_3_), 1.37 (1H, s), 1.27-1.22 (2H, m), 1.16 (2H, s), 1.12 (6H, s, 2CH_3_), 1.06 (3H, s, CH_3_), 1.02-0.99 (4H, m), 0.99-0.86 (2H, m), 0.83 (3H, s, CH_3_), 0.80-0.74 (2H, m). ^13^C NMR (CD_3_OD, *δ*): 201.03 (C11), 178.94 (C30), 170.14 (C6″), 170.05 (C6′), 169.32 (C13), 127.60 (C12), 103.85 (C1″), 103.67 (C1′), 89.23 (C3), 80.42 (C2′), 76.16 (C5″), 75.77 (C5′), 74.90 (C3″), 74.59 (C3′), 74.46 (C2″), 71.91 (C4″), 71.58 (C4′), 61.77 (C9), 55.14 (C5), 48.47 (C18), 45.34 (C8), 43.54 (C20), 43.23 (C14), 41.10 (C19), 39.37 (C4), 39.11 (C1), 37.68 (C22), 36.72 (C10), 32.44 (C7), 31.61 (C17), 30.19 (C21), 29.61 (C29), 27.93 (C28), 27.49 (C23), 27.14 (C2), 26.25 (C16), 26.02 (C15), 22.74 (C27), 18.03 (C26), 17.15 (C6), 16.01 (C24), 15.88 (C25); 2MetOMe: 171.99 (COOOMe), 171.92 (COOMe), 51.78 (α-CH), 51.58 (α-CH), 51.27 (OCH_3_), 51.06 (OCH_3_), 30.88 (CH_2_), 30.75 (CH_2_), 29.97 (CH_2_), 29.87 (CH_2_), 14.14 (CH_3_), 14.05 (CH_3_). Anal. calcd. for C_54_H_84_O_18_N_2_S_2_ C 58.25, H 7.60, N 2.52; S 5.75%; found, C 58.04, H 7.48, N 2.46, S 5.64%. M = 1113.24.

### 4.13. 3-O-{2-O-[N-(β-D-Glucopyranosyluronoyl)-L-leucine ethyl ester]-N-(β-D-glucopyranosyluronoyl)-L-leucine ethyl ester}-(3β,20β)-11-oxo-30-noroleane-12-ene-30-oic Acid (**9**) [46]

An amorphous solid, yield 56%; [α]_D_^20^ + 58° (*c* 0.07, EtOH). Lit. [46]: [α]_D_^20^ + 59° (*c* 0.12, EtOH). HPLC (96.8 ± 0.8%, τ 2.65 min) (Supplemental Figure S33). IR (*ν*, cm^−1^): 3500-3200 (OH, NH), 1739 (COOEt), 1662 (C=O), 1533 (CONH). ^1^H NMR (CD_3_OD, *δ*): 7.33 (2H, s, 2NH), 5.57 (1H, s, H12), 4.76 (1H, d, *J* = 7.7, H1″), 4.62 (1H, d, *J* = 7.7, H1′), 4.56-4.51 (4H, m), 4.17-4.09 (2H, m), 3.80-3.75 (2H, m), 3.75-3.34 (6H, m), 3.31-3.21 (3H, m), 2.93-2.67 (6H, m), 2.56 (1H, s, H9), 2.22-1.45 (14H, m), 1.40 (6H, s, 2CH_3_), 1.36 (1H, s), 1.29 (3H, s, CH_3_), 1.27-1.23 (6H, m, 2CH_3_), 1.16 (3H, s, CH_3_), 1.13 (6H, s, 2CH_3_), 1.06 (3H, s, CH_3_), 0.98-0.90 (11H, m, 3CH_3_, 2CH_2_), 0.88-0.86 (2H, m), 0.83 (6H, s, 2CH_3_), 0.79-0.76 (2H, m). ^13^C NMR (CD_3_OD, *δ*): 201.12 (C11), 178.97 (C30), 171.51 (C13), 170.22 (C6″), 170.09 (C6′), 127.53 (C12), 103.83 (C1″), 103.65 (C1′), 89.00 (C3), 80.47 (C2′), 76.23 (C5″), 75.97 (C5′), 74.92 (C3″), 74.62 (C3′), 74.34 (C2″), 72.23 (C4″), 71.66 (C4′), 61.75 (C9), 55.05 (C5), 48.50 (C18), 45.36 (C8), 43.51 (C20), 43.21 (C14), 41.04 (C19), 40.38 (C4), 39.30 (C1), 37.64 (C22), 36.67 (C10), 32.41 (C7), 31.58 (C17), 30.63 (C21), 27.84 (C29), 27.39 (C28), 27.01 (C23), 26.89 (C2), 26.20 (C16), 25.99 (C15), 22.85 (C27), 17.93 (C26), 17.10 (C6), 15.93 (C25), 15.73 (C24); 2LeuOEt: 174.26 (COOEt), 174.19 (COOEt), 50.56 (α-CH), 50.41 (α-CH), 42.89 (CH_2_), 42.55 (CH_2_), 40.59 (CH_2_), 40.38 (CH_2_), 24.63 (2CH), 22.13 (CH_3_), 22.09 (CH_3_), 20.80 (CH_3_), 20.64 (CH_3_), 13.17 (CH_3_), 13.03 (CH_3_). Anal. calcd. for C_58_H_92_N_2_O_18_ C 63.02, H 8.39, N 2.53 %; found, C 62.84, H 8.22, N 2.55%. M = 1105.33.

### 4.14. 3-O-{2-O-[N-(β-D-Glucopyranosyluronoyl)-D-leucine ethyl ester]-N-(β-D-glucopyranosyluronoyl)-D-leucine ethyl ester}-(3β,20β)-11-oxo-30-noroleane-12-ene-30-oic Acid (**10**)

An amorphous solid, yield 55%; [α]_D_^20^ + 48° (*c* 0.06, EtOH). HPLC (97.6 ± 0.8%, τ 2.74 min) (Supplemental Figure S34). IR (*ν*, cm^−1^): 3500-3200 (OH, NH), 1737 (COOEt), 1660 (C=O), 1535 (CONH). ^1^H NMR (CD_3_OD, *δ*): 7.75 (1H, br s, NH), 7.55 (1H, br s, NH), 5.57 (1H, s, H12), 4.75 (1H, d, *J* = 7.9, H1″), 4.56 (1H, d, *J* = 7.7, H1′), 4.54-4.48 (2H, m), 4.18-4.12 (1H, m), 3.78-3.75 (5H, m), 3.66-3.60 (3H, m), 3.56-3.45 (4H, m), 3.30 (4H, 2CH_2_), 3.22-3.20 (2H, m), 2.86 (1H, s, H18), 2.80-2.57 (2H, m), 2.43 (1H, s, H9), 2.20-1.60 (12H, m), 1.41 (9H, s, 3CH_3_), 1.37-1.23 (6H, m), 1.17 (3H, s, CH_3_), 1.13 (9H, s, 3 CH_3_), 1.07 (3H, s, CH_3_), 0.99-0.94 (10H, m, 2CH_3_, 2CH_2_), 0.92-0.90 (2H, m), 0.86 (3H, s, CH_3_), 0.83 (3H, s, CH_3_), 0.79-0.77 (2H, m). ^13^C NMR (CD_3_OD, *δ*): 202.69 (C11), 180.47 (C30), 172.99 (C13), 171.89 (C6″), 171.39 (C6′), 128.84 (C12), 105.05 (C1″), 104.93 (C1′), 90.48 (C3), 81.79 (C2′), 77.65 (C5′’), 77.23 (C5′), 76.48 (C3″), 76.00 (CC3′), 73.43 (C2″), 73.29 (C4″), 73.01 (C4′), 63.08 (C9), 56.36 (C5), 49.89 (C18), 46.73 (C8), 44.88 (C20), 44.59 (C14), 41.70 (C19), 40.62 (C4), 40.24 (C1), 38.97 (C22), 38.02 (C10), 33.74 (C7), 32.94 (C17), 31.97 (C21), 29.18 (C29), 28.75 (C28), 28.41 (C23), 27.55 (C16), 27.44 (C15), 26.55 (C2), 23.82 (C27), 19.28 (C26), 18.44 (C6), 17.30 (C25), 17.01 (C24); 2D-LeuOEt: 175.67 (COOEt), 175.31 (COOEt), 51.84 (α-CH), 51.73 (α-CH), 44.04 (CH_2_), 43.91 (CH_2_), 42.37 (CH_2_), 42.14 (CH_2_), 25.97 (CH), 25.91 (CH), 23.45 (2CH_3_), 22.16 (CH_3_), 21.92 (CH_3_), 14.48 (CH_3_), 14.38 (CH_3_). Anal. calcd. for C_58_H_92_N_2_O_18_ C 63.02, H 8.39, N 2.53 %; found, C 62.90, H 8.25, N 2.48 %. M = 1105.33.

### 4.15. 3-O-{2-O-[N-(β-D-Glucopyranosyluronoyl)-L-phenylalanine ethyl ester]-N-(β-D-Glucopyranosyluronoyl)-L-phenylalanine ethyl ester}-(3β, 20β)-11-oxo-30-noroleane-12-ene-30-oic Acid (**11**) [46]

An amorphous solid, yield 58%; [α]_D_^20^ + 60° (*c* 0.04, MeOH). Lit. [46]: [α]_D_^20^ + 56° (*c* 0.1, EtOH). HPLC (96.6 ± 0.8%, τ 3.82 min) (Supplemental Figure S35). IR (ν, cm^−1^): 3500-3200 (OH, NH), 1738 (COOEt), 1661 (C=O), 1652, 1529 (CONH, Ph), 1500 (Ph). ^1^H NMR (CD_3_OD, *δ*): 7.87 (2H, s, 2NH), 7.32-7.15 (10H, m, 2C_6_H_5_). 5.59 (1H, s, H12), 4.80 (1H, d, *J* = 7.7, H1″), 4.72 (2H, m), 4.58 (1H, d, *J* = 7.6, H1′), 4.15-4.09 (3H, m), 3.83-3.60 (4H, m), 3.49-3.41 (5H, m), 3.30-3.07 (6H, m), 2.69 (1H, m), 2.37 (1H, s, H9), 2.20-1.55 (14H, m), 1.38 (6H, s, 2CH_3_), 1.27-1.24 (2H, m), 1.20-1.19 (4H, m), 1.17 (3H, s, CH_3_), 1.15 (3H, s, CH_3_), 1.11 (3H, s, CH_3_), 1.07 (3H, s, CH_3_), 1.02 (3H, s, CH_3_), 0.92-0.89 (2H, m), 0.82 (3H, s, CH_3_), 0.78 (3H, s, CH_3_), 0.70-0.66 (2H, m). ^13^C NMR (CD_3_OD, *δ*): 200.85 (C11), 179.09 (C30), 170.84 (C13), 170.01 (C6″), 169.78 (C6′), 127.66 (C12), 103.48 (C1″), 103.31 (C1′), 89.27 (C3), 79.64 (C2′), 76.30 (C5″), 75.82 (C5′), 74.44 (C3″), 74.41 (C3′), 73.84 (C2″), 72.18 (C4″), 71.99 (C4′), 61.77 (C9), 55.15 (C5), 48.43 (C18), 45.36 (C8), 43.60 (C20), 43.19 (C14), 41.22 (C19), 39.34 (C4), 39.15 (C1), 37.73 (C22), 36.71 (C10), 32.44 (C7), 31.61 (C17), 30.75 (C21), 27.97 (C29), 27.56 (C28), 27.22 (C23), 26.27 (C2), 26.06 (C16), 25.91 (C15), 22.81 (C27), 18.06 (C26), 17.12 (C6), 16.08 (C25), 15.86 (C24); 2PheOEt: 171.39 (COOEt), 170.87 (COOEt), 136.17 (C Ar), 136.10 (C Ar), 129.19 (2C Ar), 129.08 (2C Ar), 128.59 (2C Ar), 128.34 (2C Ar), 128.30 (C Ar), 126.82 (C Ar), 61.37 (CH_2_), 61.23 (CH_2_), 53.51 (α-CH), 53.21 (α-CH), 37.28 (CH_2_), 36.95 (CH_2_), 13.27 (CH_3_), 13.25 (CH_3_). Anal. calcd. for C_64_H_88_N_2_O_18_ C 65.51, H 7.56, N 2.39%; found, C 65.42, H 7.68, N 2.30%. M = 1173.35.

### 4.16. 3-O-{2-O-[N-(β-D-Glucopyranosyluronoyl)-D-phenylalanine ethyl ester]-N-(β-D-glucopyranosyluronoyl)-D-phenylalanine ethyl ester}-(3β,20β)-11-oxo-30-noroleane-12-ene-30-oic Acid (**12**)

An amorphous solid, yield 56%; [α]_D_^20^ + 45° (*c* 0.12, EtOH). HPLC (98.8 ± 0.8%, τ 2.92 min) (Supplemental Figure S36). IR (ν, cm^−1^): 3500-3200 (OH, NH), 1738 (COOEt), 1661 (C=O), 1529 (CONH), 1500 (Ph). ^1^H NMR (CD_3_OD, *δ*): 7.60 (2H, d, *J* = 7.4, 2NH), 7.33-7.11 (10H, m, 2C_6_H_5_). 5.62 (1H, s, H12), 4.55-4.52 (2H, m), 4.50 (1H, d, *J* = 7.6, H1″)), 4.39 (1H, d, *J* = 7.4, H1′), 4.03-3.99 (3H, m), 3.70-3.57 (2H, m), 3.45-3.39 (10H, m), 3.30-3.18 (2H. m), 3.05-2.98 (5H, m), 2.96-2.87 (1H, m), 2.60-2.57 (1H, m, H18), 2.48 (1H, s), 2.28 (1H, s, H9), 2.06-2.04 (2H, m), 1.78-1.47 (6H, m), 1.32 (6H, s, 2CH_3_), 1.09 (2H, s), 1.08 (6H, s, 2CH_3_), 1.07 (2H, s), 1.10 (3H, s, 2CH_3_), 0.94 (3H, s, CH_3_), 0.88-0.85 (2H, m), 0.73 (3H, s, CH_3_), 0.72 (3H, s, CH_3_), 0.68-0.64 (2H, m). ^1^H NMR (DMSO-d_6_, *δ*): 7.97 (1H, d, *J* = 7.6, NH), 7.73 (1H, d, *J* = 7.4, NH), 7.31-7.15 (10H, m, 2C_6_H_5_). 5.40 (1H, s, H12), 4.67 (1H, d, *J* = 8.1, H1″)), 4.58 (1H, d, *J* = 8.2, H1′), 4.19-4.13 (5H, m), 3.63-3.43 (8H, m), 3.33-2.96 (8H, m), 2.84 (1H, m, H18), 2.40 (1H, s, H9), 2.22-1.58 (10H, m), 1.40 (6H, s, 2CH_3_), 1.27-1.18 (12H, m, 2CH_3_, 2CH_2_), 1.17 (3H, s, CH_3_), 1.13, 1.12 (6H, both s, 2CH_3_), 1.05 (3H, s, CH_3_), 0.95-0.89 (2H, m), 0.85, 0.82 (6H, both s, 2CH_3_), 0.78-0.72 (2H, m). ^13^C NMR (CD_3_OD, *δ*): 200.79 (C11), 178.90 (C30), 171.30 (C13), 170.20 (C6″), 169.89 (C6′), 127.64 (C12), 103.73 (C1″), 103.02 (C1′), 89.65 (C3), 79.04 (C2′), 76.30 (C5″), 76.00 (C5′), 74.41 (C3″), 74.14 (C3′), 74.02 (C2′), 72.27 (C4″), 72.07 (C4′), 61.77 (C9), 55.07 (C5), 48.50 (C18), 45.38 (C8), 43.56 (C20), 43.25 (C14), 41.13 (C19), 39.40 (C4), 39.11 (C1), 37.73 (C22), 37.39 (C10), 32.49 (C7), 31.68 (C17), 30.74 (C21), 28.08 (C29), 27.66 (C28), 27.27 (C23), 26.31 (C16), 26.22 (C15), 26.08 (C2), 22.85 (C27), 18.20 (C26), 17.16 (C6), 16.33 (C25), 15.93 (C24); 2D-PheOEt: 170.99 (COOEt), 170.91 (COOEt), 136.40 (C Ar), 136.02 (C Ar), 129.24 (2C Ar), 128.98 (2C Ar), 128.55 (2C Ar), 128.44 (2C Ar), 126.95 (2C Ar), 61.44 (CH_2_), 61.27 (CH_2_), 53.97 (α-CH), 53.78 (α-CH), 36.84 (CH_2_), 36.73 (CH_2_), 13.41 (CH_3_), 13.35 (CH_3_). Anal. calcd. for C_64_H_88_N_2_O_18_ C 65.51, H 7.56, N 2.39%; found, C 65.35, H 7.45, N 2.35%. M = 1173.35.

### 4.17. 3-O-{2-O-[N-(β-D-Glucopyranosyluronoyl)-L-tyrosine ethyl ester]-N-(β-D-glucopyranosyluronoyl)-L-tyrosine ethyl ester}-(3β,20β)-11-oxo-30-noroleane-12-ene-30-oic Acid (**13**)

An amorphous solid, yield 60%; [α]_D_^20^ + 62° (*c* 0.1, EtOH). HPLC (97.6 ± 0.8%, τ 2.54 min) (Supplemental Figure S37). IR (*ν*, cm^−1^): 3500-3200 (OH, NH), 1737 (COOEt), 1652 (C=O), 1615, 1596, 1516 (Tyr, CONH). ^1^H NMR (CD_3_OD, *δ*): 7.88 (2H, s, 2NH), 7.00 (2H, d, *J* = 8.2, 2H Ar), 6.96 (2H, d, *J* = 8.2, 2H Ar), 6.77 (2H, d, *J* = 8.3, 2H Ar), 6.70 (2H, d, *J* = 8.3, 2H Ar), 5.57 (1H, s, H12), 4.81 (1H, d, *J* = 7.7, H1″), 4.66 (2H, k, *J_1_* = 6.4, *J_2_* =14.2), 4.56 (1H, d, *J* =7.3 Hz, H1′), 4.16-4.09 (5H, m), 3.80-3.73 (2H, m), 3.69-3.61 (3H, m), 3.49-3.42 (4H, m), 3.30-3.13 (3H, m), 3.08-3.00 (3H, m), 2.84 (1H, s, H18), 2.68 (1H, m), 2.40 (1H, s, H9), 2.20-1.56 (10H, m), 1.42 (1H, s), 1.39 (6H, s, 2CH_3_), 1.35-1.25 (2H, m), 1.22-1.17 (7H. m, CH_3_, 2CH_2_), 1.15 (3H, s, CH_3_), 1.12 (3H, s, CH_3_), 1.08 (3H, s, CH_3_), 1.01 (3H, s, CH_3_), 0.96-0.84 (2H, m), 0.82 (3H, s, CH_3_), 0.78 (3H, s, CH_3_), 0.72-0.70 (2H, m). ^13^C NMR (CD_3_OD, *δ*): 201.24 (C11), 179.00 (C30), 171.57 (C13), 169.93 (C6′’), 169.76 (C6′), 127.57 (C12), 103.46 (C1″), 103.22 (C1′), 89.33 (C3), 79.42 (C2′), 76.46 (C5″), 75.84 (C5′), 74.41 (C3′, C3″), 73.97 (C2″), 72.11 (C4″), 72.00 (C4′), 61.73 (C9), 55.05 (C5), 48.49 (C18), 45.38 (C8), 43.57 (C20), 43.21 (C14), 41.08 (C19), 39.28 (C4), 38.94 (C1), 37.68 (C22), 36.67 (C10), 32.42 (C7), 31.59 (C17), 30.68 (C21), 27.89 (C29), 27.47 (C28), 27.14 (C23), 26.24 (C2), 26.02 (C16), 25.83 (C15), 22.61 (C27), 17.98 (C26), 17.08 (C6), 16.00 (C25), 15.73 (C24); 2D-TyrOEt: 171.34 (COOEt), 171.08 (COOEt), 156.22 (C Ar), 156.17 (C Ar), 130.21 (2C Ar), 130.07 (2C Ar), 126.60 (2C Ar), 115.14 (2C Ar), 115.03 (2C Ar), 61.28 (CH_2_), 61.21 (CH_2_), 53.68 (α-CH), 53.48 (α-CH), 36.31 (CH_2_), 36.28 (CH_2_), 13.19 (2CH_3_). Anal. calcd. for C_62_H_88_N_2_O_20_. C 63.77, H 7.36, N 2.32%; found, C 63.63, H 7.19, N 2.28%. M=1205.35.

### 4.18. 3-O-{2-O-[N-(β-D-Glucopyranosyluronoyl)-D-tyrosine ethyl ester]-N-(β-D-glucopyranosyluronoyl)-D-tyrosine ethyl ester}-(3β,20β)-11-oxo-30-noroleane-12-ene-30-acid (**14**)

An amorphous solid, yield 58%; [α]_D_^20^ + 44° (*c* 0.12, EtOH). HPLC (98.8 ± 0.8%, τ 2.75 min) (Supplemental Figure S38). IR (*ν*, cm^−1^): 3500-3200 (OH, NH), 1736 (COOEt), 1659, 1650, 1614, 1595, 1516 (Tyr, CONH). ^1^H NMR (DMSO-d_6_, *δ*): 9.25 (2H, br s, 2OH Tyr), 7.96 (1H, d, *J* = 7.3 Hz, NH), 7.62 (1H, d, *J* = 7.4 Hz, NH), 6.98 (2H, d, *J* = 8.1, 2H Ar), 6.92 (2H, d, *J* = 8.0, 2H Ar), 6.68 (2H, d, *J* = 7.7, 2H Ar), 6.64 (2H, d, *J* = 7.7, 2H Ar), 5.71 (1H, br s), 5.46 (1H, br s), 5.39 (1H, s, H12), 5.07 (1H, br s), 4.88 (1H, br s), 4.53 (1H, d, *J* = 7.0 Hz, H1′’), 4.46 (1H, d, *J* =7.0 Hz, H1′), 4.38 (2H, d, *J*=6.3 Hz), 4.00-3.98 (3H, m), 3.70-3.37 (12H, m), 3.28-2.36 (8H, m), 2.29 (1H, s, H9), 2.04-1.39 (6H, m), 1.30 (6H, s, 2CH_3_), 1.20-1.17 (2H, m), 1.09 (2H, s), 1.08 (3H, s, CH_3_), 1.06 (6H, s, 2CH_3_), 1.05 (2H. m), 0.99 (3H, s, CH_3_), 0.93 (3H, s, CH_3_), 0.78 (2H,m), 0.72 (3H, s, CH_3_), 0.69 (3H, s, CH_3_), 0.64 (2H, m). ^13^C NMR (DMSO-d_6_, *δ*): 199.54 (C11), 178.21 (C30), 170.27 (C13), 168.99 (C6′’), 168.77 (C6′), 127.10 (C12), 103.86 (C1′’, C1′), 88.79 (C3), 80.65 (C2′), 76.59 (C5′’), 76.41 (C5′), 75.29 (C3′’), 74.95 (C3′), 74.80 (C2′’), 72.05 (C4′’), 71.71 (C4′), 61.55 (C9), 54.76 (C5), 48.53 (C18), 45.32 (C8), 43.52 (C20), 43.33 (C14), 41.11 (C19), 39.24 (C4), 39.00 (C1), 37.93 (C22), 36.96 (C10), 32.53 (C7), 31.94 (C17), 30.85 (C21), 28.78 (C29), 28.44 (C28), 28.29 (C23), 27.73 (C2), 26.50 (C16), 26.18 (C15), 23.34 (C27), 18.79 (C26), 17.31 (C6), 16.54 (C25), 16.51 (C24); 2D-TyrOEt: 171.39 (COOEt), 171.28 (COOEt), 156.47 (2C Ar), 130.49 (2C Ar), 130.28 (2C Ar), 127.10 (C Ar), 127.01 (C Ar), 115.69 (2C Ar), 115.49 (2C Ar), 61.10 (CH_2_), 61.01 (CH_2_), 54.24 (α-CH), 54.12 (α-CH), 36.74 (CH_2_), 36.51 (CH_2_), 14.36 (CH_3_), 14.32 (CH_3_). Anal. calcd. for C_62_H_88_N_2_O_20_. C 63.54, H 7.05, N 2.22%; found, C 63.45, H 6.95, N 2.20%. M = 1205.35.

### 4.19. 3-O-{2-O-[N-(β-D-Glucopyranosyluronoyl)-β-alanine ethyl ester]-N-(β-D-glucopyranosyluronoyl)-β-alanine ethyl ester}- (3β, 20β)-11-oxo-30-noroleane-12-ene-30-acid (**15**)

An amorphous solid, yield 60%; [α]_D_^20^ + 54° (c 0.08, EtOH). HPLC (97.5 ± 0.8%, τ 2.66 min) (Supplemental Figure S39). ^1^H NMR (CD_3_OD, *δ*): 7.89 (2H, s, 2NH), 5.57 (1H, s, H12), 4.75 (1H, d, *J* = 7.8, H1″), 4.54 (1H, d, *J* = 7.6, H1′), 3.74-3.62 (6H, m), 3.59-3.53 (3H, m), 3.52-3.42 (8H, m), 3.31-3.28 (3H, m), 3.22-3.18 (3H, m), 2.71-2.69 (2H, m), 2.57-2.53 (5H, m), 2.43 (1H, s, H9), 2.20-2.11 (4H, m), 1.95-1.60 (12H, m), 1.45-1.42 (2H, m), 1.40 (6H, s, 2CH_3_), 1.37-1.23 (5H, m), 1.16 (3H, s, CH_3_), 1.13, 1.12 (6H, both s, 2CH_3_), 1.05 (3H, s, CH_3_), 1.03-0.90 (3H, m), 0.83 (6H, s, 2CH_3_), 0.77-0.75 (2H, m). ^13^C NMR (CD_3_OD, *δ*): 201.26 (C11), 179.02 (C30), 171.53 (C13), 170.29 (C6″), 170.23 (C6′), 127.55 (C12), 103.93 (C1″), 103.62 (C1′), 89.39 (C3), 80.89 (C2′), 76.21 (C5″), 75.78 (C5′), 74.90 (C3″), 74.57 (C3′), 74.40 (C2″), 72.04 (C4″,C4′), 61.75 (C9), 55.06 (C5), 48.50 (C18), 45.40 (C8), 43.52 (C20), 43.23 (C14), 41.02 (C19), 39.31 (C4), 38.93 (C1), 37.65 (C22), 36.64 (C10), 32.42 (C7), 31.60 (C17), 30.63 (C21), 27.88 (C29), 27.45 (C28), 27.01 (C23), 26.21 (C2), 26.00 (C16), 25.89 (C15), 22.57 (C27), 17.96 (C26), 17.04 (C6), 15.73 (C25, C24); 2β-AlaOEt: 174.10 (COOEt), 173.79 (COOEt), 34.71 (CH_2_), 34.55 (CH_2_), 33.33 (CH_2_), 33.06 (CH_2_), 15.67 (2CH_3_). Anal. calcd. for C_52_H_80_N_2_O_18_ C 61.16; H 7.89; N 2.74%; found, C 61.05; H 7.66; N 2.65. M = 1021.17.

### 4.20. 3-O-{2-O-[N-(β-D-Glucopyranosyluronoyl)-glycyl-L-phenylalanine ethyl ester]-N-(β-D-glucopyranosyluronoyl)-glycyl-L-phenylalanine ethyl ester}-(3β,20β)-11-oxo-30-noroleane-12-ene-30-acid (**16**) [46]

An amorphous solid, yield 55%; [α]_D_^20^ + 64° (c 0.08, EtOH). Lit. [46]: [α]_D_^20^ + 62˚ (c 0.08, EtOH). HPLC (97.2 ± 0.8%, τ 2.44 min) (Supplemental Figure S40). IR (*ν*, cm^−1^): 3500-3200 (OH, NH), 1739 (COOEt), 1656, 1653, 1525, 1516 (Ph, CONH). ^1^H NMR (500 MГц, CD_3_OD, δ): 7.90 (4H, br s, 4NH), 7.30-7.18 (10H, m, 2C_6_H_5_), 5.60 (1H, s, H12), 4.76 (1H, d, *J* = 7.5, H1″), 4.70-4.67 (2H, m), 4.53 (1H, d, *J* = 7.5, H1′), 4.14-4.05 (2H, m), 3.94-3.72 (2H, m), 3.69 (2H, s), 3.67, 3.66 (4H, both s), 3.64-3.28 (4H, m), 3.15-2.98 (4H, m), 2.76-2.69 (4H, m), 2.62 (1H, s, H9), 2.57-2.38 (4H, m), 2.23-1.57 (12H, m), 1.39 (6H, s, 2CH_3_), 1.28-1.17 (5H, m), 1.14 (3H, s, CH_3_), 1.12 (6H, s, 2CH_3_), 1.09 (3H, s, CH_3_), 1.02 (3H, s, CH_3_), 0.97-0.85 (2H, m), 0.82 (3H, s, CH_3_), 0.76 (3H, s, CH_3_), 0.76-0.72 (2H, m). ^13^C NMR (MHz, CD_3_OD, *δ*): 201.04 (C11), 180.02 (C30), 170.71 (C13), 169.43 (C6″), 169.26 (C6′), 127.53 (C12), 103.88 (C1″), 103.61 (C1′), 89.42 (C3), 80.92 (C2′), 76.53 (C5″), 76.20 (C5′), 75.94 (C3″), 75.78 (C3′), 74.55 (C2″), 72.14 (C4″), 72.01 (C4′), 61.76 (C9), 54.99 (C5), 48.54 (C18), 45.34 (C8), 43.82 (C20), 43.20 (C14), 42.32 (C19), 39.38 (C4), 38.93 (C1), 37.76 (C22), 36.69 (C10), 32.40 (C7), 31.59 (C17), 30.84 (C21), 27.91 (C29), 27.63 (C28), 26.90 (C23), 26.83 (C2), 26.22 (C16), 26.04 (C15), 22.50 (C27), 17.96 (C26), 17.04 (C6), 15.77 (C25), 15.50 (C24); Gly-PheOEt: 171.88 (COOEt), 171.57 (COOEt), 166.96 (CONH), 166.76 (CONH), 136.75 (C Ar), 136.54 (C Ar), 130.10 (C Ar), 128.92 (2C Ar), 128.21 (2C Ar), 128.13 (2Ar-C), 126.66 (C Ar), 126.60 (C Ar), 66.64 (CH_2_), 66.32 (CH_2_), 53.92 (α-CH), 53.87 (α-CH), 41.54 (CH_2_), 41.33 (CH_2_), 37.21 (CH_2_), 37.09 (CH_2_), 9.30 (2CH_3_). Anal. calcd. for C_68_H_94_N_4_O_20_ C 63.43; H 7.36; N 4.35%; found, C 63.32; H 7.26; N 4.25. M = 1287.45.

### 4.21. 3-O-{2-O-[N-(β-D-Glucopyranosyluronoyl)-L-valine]-N-(β-D-glucopyranosyluronoyl)-L-valine}-(3β,20β)-11-oxo-30-noroleane-12-ene-30-oic Acid (**17**) [47]

An amorphous solid, yield 54%; [α]_D_^20^ + 57° (*c* 0.06; EtOH). Lit. [47]: [α]_D_^20^ + 55° (*c* 0.06; EtOH). HPLC (98.0 ± 0.8%, τ 2.66 min) (Supplemental Figure S41); IR (ν, sm^−1^): 3500-3200 (OH, NH), 1718 (COOH), 1662 (C=O), 1528 (CONH). ^1^H NMR (CD_3_OD, *δ*): 7.89 (2H, s, 2NH), 5.72 (1H, s, H12), 4.76 (1H, d, *J* = 7.2, H1″), 4.60 (1H, d, *J* = 7.0, H1′), 4.42-4.33 (3H, m, H3″, H3′, H3), 3.84-3.76 (4H, m), 3.52-3.45 (4H, m); 3.35-3.21 (6H, m), 2.43 (1H, c, H9), 2.21-2.18 (2H, m), 1.99-1.60 (12H, m), 1.45 (3H, s, CH_3_), 1.28 (3H, s, CH_3_), 1.25-1.20 (2H, m), 1.15 (3H, s, CH_3_), 1.12 (6H, s, 2CH_3_), 1.05 (3H, s, CH_3_), 1.02-0.94 (13H, m, 3CH_3_, 2CH_2_), 0.90-0.88 (2H, m), 0.84 (3H, s, CH_3_), 0.80 (3H, s, CH_3_), 0.76-0.74 (2H, m). ^13^C NMR (CD_3_OD, *δ*): 202.70 (C11), 178.62 (C30), 172.44 (C13), 171.68 (C6″), 171.57 (C6′), 129.20 (C12), 105.00 (C1″), 104.86 (C1′), 90.29 (C3), 81.66 (C2′), 77.80 (C5″), 77.35 (C5′), 76.36 (C3′’), 76.08 (C3′), 75.24 (C2″), 73.76 (C4′,C4″), 63.20 (C9), 56.49 (C5), 48.20 (C18), 46.79 (C8), 45.05 (C20), 44.65 (C14), 42.77 (C19), 40.73 (C4), 40.36 (C1), 38.78 (C22), 38.15 (C10), 33.92 (C7), 33.00 (C17), 31.80 (C21), 29.40 (C29), 29.14 (C23), 28.51 (C28), 27.69 (C16), 27.49 (C15), 26.10 (C2), 23.77 (C27), 19.84 (C26), 18.00 (C6), 17.29 (C24), 16.97 (C25). 2Val: 174.95 (COOH), 174.22 8(COOH), 58.90 (α-CH), 58.35 (α-CH), 32.25 (CH_2_), 32.09 (CH_2_), 19.58 (CH_3_), 19.47 (CH_3_), 18.73 (CH_3_), 18.49 (CH_3_). Anal. calc. for C_52_H_80_N_2_O_18_. C 61.16, H 7.90, N 2.74%; found C 60.95, H 7.80, N 2.65. E = 1021.17.

### 4.22. 3-O-{2-O-[N-(β-D-Glucopyranosyluronoyl)-L-leucine]-N-(β-D-glucopyranosyluronoyl)-L-leucine}-(3β,20β)-11-oxo-30-noroleane-12-ene-30-oic Acid (**18**) [46,47]

An amorphous solid, yield 56%; [α]_D_^20^ + 63° (c 0.06, EtOH). Lit. [46]: [α]_D_^20^ + 65° (c 0.06, EtOH). HPLC (98.7 ± 0.8%, τ 2.70 min) (Supplemental Figure S42); IR (ν, sm^−1^): 3500-3200 (OH, NH), 1720 (COOH), 1662 (C=O), 1540 (CONH). ^1^H NMR (CD_3_OD, *δ*): 7.89 (2H, s, 2NH), 5.58 (1H, s, H12), 4.78 (1H, d, *J* = 7.8, H1″), 4.58 (1H, d, *J* = 7.6, H1′), 4.55-4.50 (2H, m), 3.83-3.68 (5H, m), 3.64-3.45 (5H, m), 3.30-3.20 (2H, m), 2.72-2.66 (3H, m), 2.41 (1H, s, H9), 2.24-1.83 (6H, m), 1.71-1.60 (10H, m), 1.40 (6H, s, 2CH_3_), 1.36 (1H, s), 1.33-1.23 (2H, m), 1.16 (3H, s, CH_3_), 1.12 (6H, br s, 2CH_3_), 1.05 (3H, s, CH_3_), 0.98-0.91 (16H, m), 0.83 (6H, s, 2CH_3_), 0.77-0.74 (2H, m). ^13^C NMR (CD_3_OD, *δ*): 201.11 (C11), 179.01 (C30), 171.60 (C13), 170.24 (C6″), 169.80 (C6′), 127.53 (C12), 103.84 (C1″), 103.66 (C1′), 88.98 (C3), 80.50 (C2′), 76.11 (C5″), 75.68 (C5′), 74.82 (C3″), 74.62 (C3′), 74.26 (C2″), 72.20 (C4″), 71.79 (C4′), 61.75 (C9), 55.05 (C5), 48.54 (C18), 45.36 (C8), 43.53 (C20), 43.21 (C14), 41.05 (C19), 39.31 (C4), 39.04 (C1), 37.65 (C22), 36.67 (C10), 32.41 (C7), 31.59 (C17), 30.63 (C21), 27.90 (C29), 27.44 (C28), 27.05 (C23), 26.31 (C2), 26.21 (C16), 25.98 (C15), 22.64 (C-27), 17.96 (C26), 17.10 (C6), 15.96 (C25), 15.96 (C24); 2Leu: 174.43 (2COOH), 50.83 (α-CH), 50.67 (α-CH), 40.50 (CH_2_), 40.30 (CH_2_), 24.64 (2CH), 22.22 (CH_3_), 22.18 (CH_3_), 20.83 (CH_3_), 20.66 (CH_3_). Anal. calcd. for C_54_H_84_N_2_O_18_ C 61.81, H 8.07, N 2.67; found, C 61.62, H 7.88, N 2.54. M = 1049.22.

### 4.23. 3-O-{2-O-[N-(β-D-Glucopyranosyluronoyl)-L-isoleucine]-N-(β-D-glucopyranosyluronoyl)-L-isoleucine}-(3β,20β)-11-oxo-30-noroleane-12-ene-30-oic Acid (**19**) [47,49]

An amorphous solid, yield 55%; [α]_D_^20^ + 56° (*c* 0.04; EtOH). Lit. [48]: [α]_D_^20^ + 58° (*c* 0.06; MeOH). HPLC (95.8 ± 0.8%, τ 2.78 min) (Supplemental Figure S43); IR (ν, cm^−1^): 3500-3200 (OH, NH), 1716 (COOH), 1662 (C=O), 1539 (CONH). ^1^H NMR (CD_3_OD, *δ*): 7.87 (2H, s, 2NH), 5.57 (1H, s, H12), 4.79 (1H, d, *J* = 7.5, H1′’), 4.60 (1H, d, *J* = 7.4, H1′), 4.42-4.38 (2H, m), 3.87-3.50 (10H, m), 3.34-3.30 (2H, m), 3.22-3.18 (4H, m), 2.68-2.62 (2H, m), 2.41 (1H, s, H9), 2.18-1.68 (14H, m), 1.40 (6H, br s, 2CH_3_), 1.31 (1H, s), 1.30 (3H, s, CH_3_), 1.28 (1H, s), 1.24-1.22 (2H, m), 1.16 (3H, s, CH_3_), 1.11 (6H, br s, 2CH_3_), 1.06 (2H, br s), 1.01 (1H, br s), 0.98 (2H, s), 0.97 (3H, s, CH_3_), 0.95 (3H, s, CH_3_), 0.93 (3H, s, CH_3_), 0.84 (2H, s), 0.82 (3H, s, CH_3_), 0.80-0.78 (2H, m). ^13^C NMR (CD_3_OD, *δ*): 201.13 (C11), 179.10 (C30), 171.54 (C13), 170.11 (C6″), 170.04 (C6′), 127.54 (C12), 104.90 (C1″), 103.52 (C1′), 88.85 (C3), 80.07 (C2′), 76.22 (C5″), 75.77 (C5′), 74.74 (C3″), 74.59 (C3′), 73.49 (C2″), 72.15 (C4″, C4′), 61.75 (C-9), 54.99 (C5), 48.51 (C18), 46.50 (C8), 45.39 (C20), 43.54 (C14), 41.06 (C19), 39.35 (C4), 38.94 (C1), 37.67 (C22), 36.69 (C10), 32.46 (C7), 31.62 (C17), 30.67 (C21), 27.96 (C29), 27.52 (C28), 27.12 (C23), 26.25 (C2), 26.04 (C16, C15), 22.64 (C27), 18.06 (C26), 17.09 (C6), 15.98 (C25), 15.78 (C24). 2Ile: 173.35 (2COOH), 56.97 (α-CH), 56.59 (α-CH), 37.43 (CH), 37.18 (CH), 25.14 (CH_2_), 24.73 (CH_2_), 14.82 (2CH_3_), 10.91 (CH_3_), 10.79 (CH_3_). Anal. calcd. for C_54_H_84_N_2_O_18_ C 61.81, H 8.07, N 2.67; found, C 61.65, H 7.90, N 2.55. M = 1049.22.

### 4.24. 3-O-{2-O-[N-(β-D-Glucopyranosyluronoyl)-L-phenylalanine]-N-(β-D-glucopyranosyluronoyl)-L-phenylalanine}-(3β,20β)-11-oxo-30-noroleane-12-ene-30-oic Acid (**20**) [47,49]

An amorpous solid, yield 56%; [α]_D_^20^ + 60° (c 0.04, MeOH). Lit. [48]: [α]_D_^20^ + 59° (c 0.04, MeOH). HPLC (96.5 ± 0.8%, τ 2.74 min) (Supplemental Figure S44); IR (ν, cm^−1^): 3500-3200 (OH, NH), 1716 (COOH), 1657 (C=O), 1538 (CONH). ^1^H NMR (CD_3_OD, *δ*): 7.61-7.18 (10H, m, 2C_6_H_5_), 5.57 (1H, s, H12), 4.78-4.53 (4H, m), 4.20-4.10 (2H, m), 3.78-3.40 (8H, m), 3.34-3.12 (8H, m), 2.63-2.60 (2H, m), 2.37 (1H, br s, H9), 2.19-2.10 (2H, m), 1.93-1.54 (12H, m), 1.38 (6H, br s, 2CH_3_), 1.29-1.22 (2H, m), 1.16 (3H, s, CH_3_), 1.10 (3H, s, CH_3_), 1.05-1.02 (4H, m), 0.96-0.84 (2H, m), 0.81 (6H, s, 2CH_3_), 0.78-0.74 (2H, m).^13^C NMR (CD_3_OD, *δ*): 201.15 (C11), 179.01 (C30), 170.60 (C13), 169.94 (C6′, C6″), 127.55 (C12), 104.80 (C1″), 103.44 (C1′), 89.36 (C3), 82.30 (C2′), 76.10 (C5″), 75.91 (C5′), 74.85 (C3′’), 74.40 (C3′), 73.60 (C2″), 72.03 (C4″), 71.64 (C4′), 61.78 (C9), 55.22 (C5), 48.61 (C18), 45.37 (C8), 43.53 (C20), 43.21 (C14), 41.04 (C19), 39.22 (C4), 38.98 (C1), 37.67 (C22), 36.06 (C10), 32.44 (C7), 31.62 (C17), 30.66 (C21), 27.95 (C29), 27.52 (C28), 26.99 (C23), 26.23 (C2), 26.02 (C15, C16), 22.65 (C27), 18.04 (C26), 17.07 (C6), 15.80 (C25), 15.58 (C24). 2Phe: 172.51 (COOH), 171.48 (COOH), 136.39 (C Ar), 136.18 (C Ar), 129.24 (2C Ar), 129.15 (C Ar), 128.91 (2C Ar), 128.27 (2C Ar), 126.80 (C Ar), 53.30 (α-CH), 52.92(α-CH), 36.88 (CH_2_), 36.63 (CH_2_). Anal. calcd. for C_60_H_80_N_2_O_18_. C 64.49, H 7.22, N 2.50%; found, C 64.35, H 7.10, N 2.40. M = 1117.24.

### 4.25. 3-O-{2-O-[N-(β-D-Glucopyranosyluronoyl)-L-lysine(ϵ-carbobenzoxy)]-N-(β-D-glucopyranosyluronoyl)-L-lysine(ϵ-carbobenzoxy)}-(3β,20β)-11-oxo-30-noroleane-12-ene-30-oic Acid (**21**) [46,51]

An amorphous solid, yield 54%; [α]_D_^20^ + 49° (*c* 0.05, MeOH). Lit. [50]: [α]_D_^20^ + 47° (*c* 0.02, MeOH). HPLC (95.8 ± 0.8%): τ = 2.83 min (Supplemental Figure S45); IR (ν, cm^−1^): 3500-3200 (OH, NH), 1714 (COOH), 1662 (C=O), 1532 (CONH). ^1^H NMR (DMSO-d_6_, δ): 7.70 (1H, br s, NH),, 7.54 (1H, br s, NH), 7.27-7.22 (10H, m, 2C_6_H_5_), 7.00 (2H, br s, 2NH), 5.41 (1H, s, H12), 4.95 (4H. s), 4.56-4.17 (5H, m), 3.65-3.40 (15H, m), 3.32-2.96 (10H, m), 2.48 (1H, s, H9), 2.22-1.98 (8H, m), 1.80-1.39 (15H, m), 1.28 (6H, br s, 2CH_3_), 1.18 (2H, s), 1.04 (3H, s, CH_3_), 1.01 (2H, m), 0.98 (6H, s, 2CH_3_), 0.92 (3H, s, CH_3_), 0.88-0.78 (2H, m), 0.71, 0.70 (6H, both s, 2CH_3_), 0.64-0.62 (2H, m). ^13^C NMR (DMSO-d_6_, *δ*): 199.54 (C11), 178.15 (C30), 170.25 (C13), 168.73 (C6″), 168.62 (C6′), 127.49 (C12), 104.81(C1″), 103.88 (C1′), 88.38 (C3), 82.23 (C2′), 76.40 (C5″), 76.05 (C5′), 75.21 (C3″), 75.06 C3′), 72.88 (C2″), 72.13 (C4″), 71.39 (C4′), 61.51 (C9), 54.79 (C5), 48.42 (C18), 45.24 (C8), 43.44 (C20), 43.22 (C14), 40.52 (C19), 39.31 (C4), 39.14 (C1), 37.50 (C22), 36.67 (C10), 31.86 (C7), 31.53 (C17); 28.70 (C29), 28.20 (C28); 27.58 (C23), 26.50 (C2), 26.05 (C16); 25.86 (C15), 23.26 (C27); 18.65 (C26, C6); 16.48 (C25, C24); 2Lys(Z): 173.98 (2COOH), 156.50 (2C Ar), 137.62 (C Ar), 137.55 (C Ar), 130.50 (C Ar), 128.73 (2C Ar), 128.12 (C Ar), 128.05 (C Ar), 127.97 (C Ar), 127.65 (C Ar), 122.38 (C Ar), 65.87 (CH_2_), 65.54 (CH_2_), 52.45 (α-CH), 51.93 (α-CH), 29.53 (CH_2_), 29.40 (CH_2_), 23.04 (CH_2_), 22.63 (CH_2_). Anal. calcd. for C_70_H_98_N_4_O_22_ C 62.38, H 7.33, N 4.16%; found, C 62.24, H 7.23, N 4.20%. M = 1347.56.

### 4.26. Viruses and Cells

DENV-1 isolate CMUH 2018-4 and DENV-2 strain 16681 used in this study were propagated in monkey kidney epithelial Vero E6 cells (Bioresource Collection and Research Center (BCRC), Hsinchu, Taiwan). The cultured media of infected Vero E6 cells were harvested 5 days post-infection, and virus titer of the stock was determined using the mean (50%) tissue culture infectious dose (TCID50) per mL [45,59,60]. Vero E6 cells and human lung epithelial A549 cells (BCRC, Hsinchu, Taiwan) that were cultured in DMEM supplemented with 10% fetal bovine serum (FBS) and penicillin/streptomycin, were used to explore antiviral potential and the mechanisms of GL conjugates with methyl or ethyl esters of L- and D-amino acids (Compounds **2**–**21**) against DENV1 and DENV2.

### 4.27. Cytotoxicity Assay

Vero E6 cells and A549 cells (5 × 10^3^ cells/well) were treated with the serial dilution of GL conjugates at 0, 0.1, 10, 20, and 50 μM for examining the in vitro cytotoxicity of tested compounds. After 96-h incubation at 37 °C, the cells were incubated with MTT (3-(4,5-Dimethylthiazol-2-yl)-2,5-Diphenyltetrazolium Bromide) solution for another 4 h incubation, mixed with DMSO for 15 min after the removal of media, and then measured the absorbance at 570 nm. The viability (%) of treated cells was quantified according to the reduced ability of MTT. Finally, the concentration for reducing 50% cell viability (the 50% cytotoxic concentration, CC_50_) was determined by regression analysis of the concentration–response survival curve.

### 4.28. Antiviral Activity Assays

In the antiviral screening assay, each tested GL conjugate at 10 μM was used to survey the anti-DENV activity in Vero E6 cells using cytopathic effect (CPE) and infectivity reduction assays as described in our prior report [45,59,60]. In CPE and infectivity reduction assays, Vero E6 cells were infected by DENV2 at an MOI of 0.005 and simultaneously treated 10 μM of indicated GL conjugate for 96 h, and then photographed by microscope for examining the levels of DENV2-induced CPE in infected cells treated with or without GL conjugate. Meanwhile, the cells were further performed using immunofluorescence assay (IFA) with anti-DENV NS4B antibodies (GeneTex, Inc., Hsinchu, Taiwan) and anti-rabbit IgG antibodies conjugated with AF555 (ThermoFisher, Waltham, MA, USA) plus DAPI (4′,6-diamidino-2-phenylindole) nuclear staining. Finally, the DENV-2 infectivity was measured according to the ratio of DENV NS4B-positive cells to DAPI-stained cells in the well of mock-treated or treated cells, as described in [45,59,60]. In the antiviral potency assay, Vero E6 and A549 cells were infected with the fixed amount of DENV (DENV1 at an MOI of 0.1, or DENV2 (at MOIs of 0.05 and 0.005), and simultaneously treated with 0.1, 1, and 10 μM of active GL conjugates (Compounds **3**, **6**, **11**, and **21**), respectively. After 96-h incubation, the cells were performed using CPE and infectivity reduction assays, as described above. Finally, the concentration for reducing 50% viral infectivity (the 50% inhibitory concentration, IC_50_) was calculated by regression analysis of the concentration-response infectivity reduction curve. In addition, selectivity index (SI) was a measure based on the ratio of CC_50_ to IC_50_.

### 4.29. DENV2 Yield Reduction Assay

Ten-fold serial dilutions of the cultured media of treated/infected cells harvested in the antiviral potency assay were applied to a monolayer of Vero E6 cells in the 96-well plates. After 96-h incubation, the number of CPE in each well of the dilution was calculated, and the indicated dilution with a 50% positivity rate of CPE in the cell culture was expressed as the mean (50%) tissue culture infectious dose (TCID50). Finally, virus yield in cultured media of treated/infected cells was determined using above end-point dilution assay and displayed as TCID50 per milliliter, as described by the Spearman–Kerber method [61].

### 4.30. Time-of-Addition/Compound Removal Assay

The time-of-addition assays consisted of three modes including attachment treatment/removal, entry-treatment/removal, and post-entry treatment/removal modes [59,60]. In the attachment-treatment/removal mode, Vero E6 cells monolayer in 6-well plate was placed at 4 °C for 2 h and incubated with the fresh-prepared mixture of DENV-2 (MOI = 0.005) with the active compound at indicated concentrations for 2 h at 4 °C, and then washed with phosphate-buffered saline (PBS). In the entry-treatment/removal mode, Vero E6 cells were simultaneously treated with the active compound and infected DENV-2 (MOI = 0.005) in 6-well plates, incubated for 1 h at 37 °C, and then washed with PBS. In the post-entry treatment/removal mode, the cells were infected with DENV-2 for 1 h at 37 °C, treated with the active compounds at indicated concentrations for 1 h at 37 °C, respectively, and then washed by PBS. After active compound removal (washout), the cells were incubated at 37 °C for 96 h, and then were analyzed by immunofluorescent and DAPI staining assay for determining the residual DENV-2 infectivity, as described above. The inhibitory activity of the transient treatment by active compounds on attachment, entry, and post-entry stages of DENV replication in vitro was determined based on the residual infectivity [59,60].

### 4.31. Targeting Viral E Protein-Mediated Attachment Assay with Chimeric DENV2 CprME/JEV and Chimeric DENV2 prME/ZIKV Single-Round Infectious Particles (SRIPs)

DNA-launched cytomegaloviruses (CMV) promoter-driven JEV and ZIKV replicons had been constructed, as described in our prior reports [62,63]. Wild type JEV and ZIKV single-round infectious particles (SRIPs) were collected in the cultured media of JEV C-prM-E and ZIKV prM-E protein-expressing cell lines post-transfection with JEV and ZIKV replicons, respectively [62,63]. To produce chimeric DENV2-CprME/JEV replicon SRIPs, the coding regions of DENV2 C-prM-E proteins were amplified using PCR with specific primer pairs, and then cloned into the expression plasmid pcDNA3.1-His-C. The primer pairs were 5′-GATGACGAC AAGCTTGCGGCCGCGATGAATGACCAACGGAAAAAG-3′ and 5′-GATGCCACCCGGGATCCTCTAGATTAGGCCTGCACCATGACTCCCAAATAC-3′ for DENV2 C-prM-E gene fragments. For generating chimeric DENV2 prME/ZIKV replicon SRIPs, the coding regions of DENV2 prM-E proteins were amplified using PCR with specific primer pairs, and then cloned into the expression plasmid pcDNA3.1-His-C. The primer pairs were 5′-GTGGAATTCTTCCATTTAACCACACGT-3′ and 5′-GATGCCACCCGGGATCCTCTAGATTAGGCCTGCACCATGACTCCCAAATAC-3′ for DENV2 prM-E gene fragments. After sequencing to make sure no mutation in DENV2 structure protein genes, resultant plasmids pcDNA3.1-DENV2 C-prM-E and pcDNA3.1-DENV2 prM-E were transfected onto 90% confluence of the cells grown in a 6-wells plate using Lipofectamine LTX (Invitrogen) according to the manufacturer’s guidelines. The transfected cells were selected by the treatment with 500 µg/mL of G418 for 14 days, as DENV2 C-prM-E DENV2 and prM-E stably expressing cell lines, respectively. Next, chimeric DENV2 CprME/JEV SRIPs were harvested from the cultured media of C-prM-E stably expressing cells transfected with JEV replicon, and chimeric DENV2 prME/ZIKV SRIPs were collected from the cultured media of prM-E stably expressing cells transfected with ZIKV replicon after a 5-day incubation. In the attachment-treatment/compound-removal assay, Vero E6 cell monolayer was pre-cooling at 4 °C for 1 h, and incubated with the fresh-prepared mixture of the active compound with chimeric DENV2 CprME/JEV, DENV2 prME/ZIKV, wild-type JEV or ZIKV SRIPs (MOI = 1) at 4 °C for 1 h, and then washed with PBS. After the removal of the mixture (the compound and SRIP), the cells were incubated at 37 °C for 18 h, and then were analyzed by immunofluorescent assay with anti-JEV NS3 and anti-ZIKV NS5 antibodies, respectively, plus DAPI staining for determining the residual infectivity of wild-type and chimeric SRIPs, as described above.

### 4.32. Docking Studies to DENV-2 E Protein

Molecular docking of the potential lead compounds to the DENV-2 E protein (UniprotKB accession: P18356, residue 1-495) was performed by BIOVIA Discovery Studio software using the cryo-EM structure of DENV-2 E protein (PDB: 7KV8) [64]. The generated docking poses were further evaluated by Libdock scores. The docked complexes were presented by PyMOL 2.3.3.

### 4.33. Statistical Analysis

The *p*-value of the data from three repeats in the designed experiments was determined using one-way ANOVA and Scheffe’s post-hoc test using SPSS 12.0 (SPSS, Inc., Chicago, IL, USA). A *p*-value of less than 0.05 was judged as a statistically significant result of the test.

## 5. Conclusions

The performed antiviral studies of a library of known and new GL derivatives bearing L- or D-amino acids residues in the carbohydrate part of glycoside revealed three hit compounds GL-D-ValOMe **3,** GL-TyrOMe **6,** GL-PheOEt **11,** which displayed high inhibitory activity against DENV1 and DENV 2 in Vero E6 and A549 cells. GL conjugates **3** and **11** affected the virus binding stage to the cell surface in the attachment mode of the time-of-addition assay, and the GL conjugate with GL-PheOEt **11** is superior in this test D-ValOMe **3**. The E protein-mediated attachment assay with chimeric DENV2 CprME/JEV replicon and DENV2 prME/ZIKV replicon SRIPs revealed that GL conjugates **3** and **11** significantly reduced the attachment of chimeric DENV2 CprME/JEV and DENV2 prME/ZIKV SRIPs in Vero E6 cells in concentration-dependent manners, confirming the putative inhibitory effect on DENV E-mediated attachment of Compounds **3** and **11.** Among the discovered hit GL conjugates, Compounds **3** and **11** may be chosen as the lead molecules perspective for the development of DENV E protein inhibitors due to the simple synthetic procedure for their preparation and high inhibitory activity against DENV2. Thus, the conjugation of GL derivatives with amino acids methyl/ethyl esters is a prospective way to produce the new potent DENV inhibitors. Altogether, our findings provide new insights into the structure–activity relationship of GL derivatives conjugated with amino acid residues and can be the new fundamental basis for the search and development of novel flaviviruses inhibitors based on natural compounds. GL derivative leads will be the subject of abundant studies of antiviral activity against other flaviviruses and flaviviruses targets (NS2B-NS3 protease, NS3 helicase, and NS5 RNA-dependent RNA polymerase).

## Figures and Tables

**Table 1:** Antiviral activity of Glycyrrhizic acid derivative against Dengue virus type 2.

Compounds			Vero E6 Cells			A549 Cells		
	CPE Reduction at 10 μM ^a^	Inhibition (%) at 10 μM ^b^	Cytotoxicity (CC_50_, μM) ^c^	Inhibition of DENV2 Infectivity (IC_50_, μM) ^e^	Selectivity Index (CC_50_/ IC_50_)	Cytotoxicity (CC_50_, μM) ^c^	Inhibition of DENV2 Infectivity (IC_50_, μM) ^e^	Selectivity Index (CC_50_/ IC_50_)
**1**	+++	70.5	>100 μM	8.1 ± 0.21	>12.3	ND	ND	ND
**2**	−	ND ^d^	ND	ND	ND	ND	ND	ND
**3**	++++	90.7	>100 μM	0.50 ± 0.17	>201.6	>100 μM	0.12 ± 0.028	>833
**4**	−	ND	ND	ND	ND	ND	ND	ND
**6**	++++	94.0	>100 μM	5.98 ± 0.41	>16.7	>100 μM	<0.1	>1000
**7**	++	51.7	ND	ND	ND	ND	ND	ND
**8**	++	51.6	ND	ND	ND	ND	ND	ND
**9**	+++	68.9	ND	ND	ND	ND	ND	ND
**11**	++++	91.3	>100 μM	0.18 ± 0.01	>552	>100 μM	1.56 ± 0.49	>64
**15**	++	53.6	ND	ND	ND	ND	ND	ND
**16**	+++	63.8	ND	ND	ND	ND	ND	ND
**18**	+++	63.1	ND	ND	ND	ND	ND	ND
**21**	++++	92.8	>100 μM	2.68 ± 0.54	>37.3	>100 μM	0.97 ± 0.34	>103

^a^ −, no effect; ++, 25–50% inhibition of CPE; +++, 50–75%; ++++, 75–100%. ^b^ Inhibitory rate = (1 − % of NS4B-positive treated infected cells/% of NS4B-positive mock-treated infected cells) * 100. ^c^ 50% cytotoxic concentration of the derivative that inhibited 50% of viability in treated cells. ^d^ not detected. ^e^ 50% antiviral concentration of the derivative that inhibited 50% of infectivity in treated, infected cells.

## Data Availability

Not applicable.

## Supplementary Materials

The following supporting information can be downloaded at: https://www.mdpi.com/article/10.3390/ijms231810309/s1.

## Abbreviations

GL	Glycyrrhizic acid
DENV	Dengue virus
ZIKV	Zika virus
JEV	Japanese Encephalitis Virus
WNV	West Nile virus
RdRp	RNA-dependent RNA polymerase
EDC	1-ethyl-3-(3-dimethylaminopropyl)carbodiimid hydrochloride
DMF	*N,N*′-Dimethylformamide
HOSu	*N*-hydroxysuccinimide
DCC	*N,N*′-dicyclohexylcarbodiimide
IR	Infrared
NMR	Nuclear magnetic resonance
Hz	Hertz
J	Coupling constants
s	Singlet
d	Doublet
m	Multiplet
DMSO	Dimethyl sulfoxide
EtOH	Ethanol
MeOH	Methanol
Me	Methyl
Z	Carbobenzoxy
CC	Column chromatography
TLC	Thin-layer chromatography
HPLC	High-performance liquid chromatography
τ	Retention time
CPE	Cytopathic effect
IFA	Immunofluorescence assay
MTT	3-(4,5-Dimethylthiazol-2-yl)-2,5-Diphenyltetrazolium Bromide
IC50	50% inhibitory concentration
CC50	50% cytotoxic concentration
SI	Selectivity index
TCID50	50%Tissue culture infectious dose
MOI	multiplicity of infection
DAPI	4′,6-diamidino-2-phenylindole
PBS	Phosphate-buffered saline
C-prM-E	capsid-pr-membrane-envelope protein
SRIP	single-round infectious particle
BHK-21	Baby Hamster Kidney-21
CMV	Cytomegalovirus
PCR	polymerase chain reaction
