# Supplementary material for: Glycyrrhizic Acid Derivatives Bearing Amino Acid Residues in the Carbohydrate Part as Dengue Virus E Protein Inhibitors: Synthesis and Antiviral Activity
Source: Int J Mol Sci. 2022 Sep 7;23(18):10309. doi: 10.3390/ijms231810309 (PMC9499324; doi:10.3390/ijms231810309)
Supplement: Supplementary file 1 [file ijms-23-10309-s001.zip › ijms-1865300-supplementary.pdf]

Supplementary materials

Glycyrrhizic acid derivatives bearing amino acids residues in the carbohydrate part as Dengue virus E protein inhibitors: synthesis and antiviral activity

Mann-Jen Hour [1], Yeh Chen [2,¶], Chen-Sheng Lin [3,¶], Lidia A. Baltina [4,*], Ju-Ying Kan [5], Yan-Ting Tsai [6,7], Yan-Tung Kiu [6], Hsueh-Chou Lai [8], Lia A. Baltina [4], Svetlana F. Petrova [4], Cheng-Wen Lin [5,6,8,*]

[¶]Co-first author
*Corresponding authors:
Professor Lidia A. Baltina, Ufa Institute of Chemistry, Ufa Federal Research Centre of RAS, 71, prospect Oktyabrya, Ufa 450054, Russian Federation, 71, prospect Oktyabrya, Ufa 450054, Russian Federation.
Professor Cheng-Wen Lin, Department of Medical Laboratory Science and Biotechnology, China Medical University, 91, Hsueh-Shin Rd., Taichung 40402, Taiwan, ROC.
E-mail address: baltina@anrb.ru; lidiabal1810@gmail.com (L.A. Baltina); cwlin@mail.cmu.edu.tw (C.W. Lin)

Part I: Supplemental Figures 1-23 for NMR spectra of novel GL derivatives and compounds-leads

Part II: Supplemental Figures 24 and 25 for Antiviral data

Part III: Supplementa1 Figures 26-45 for HPLC analysis for GL derivatives

Part I: NMR spectra of novel GL derivatives and compounds-leads

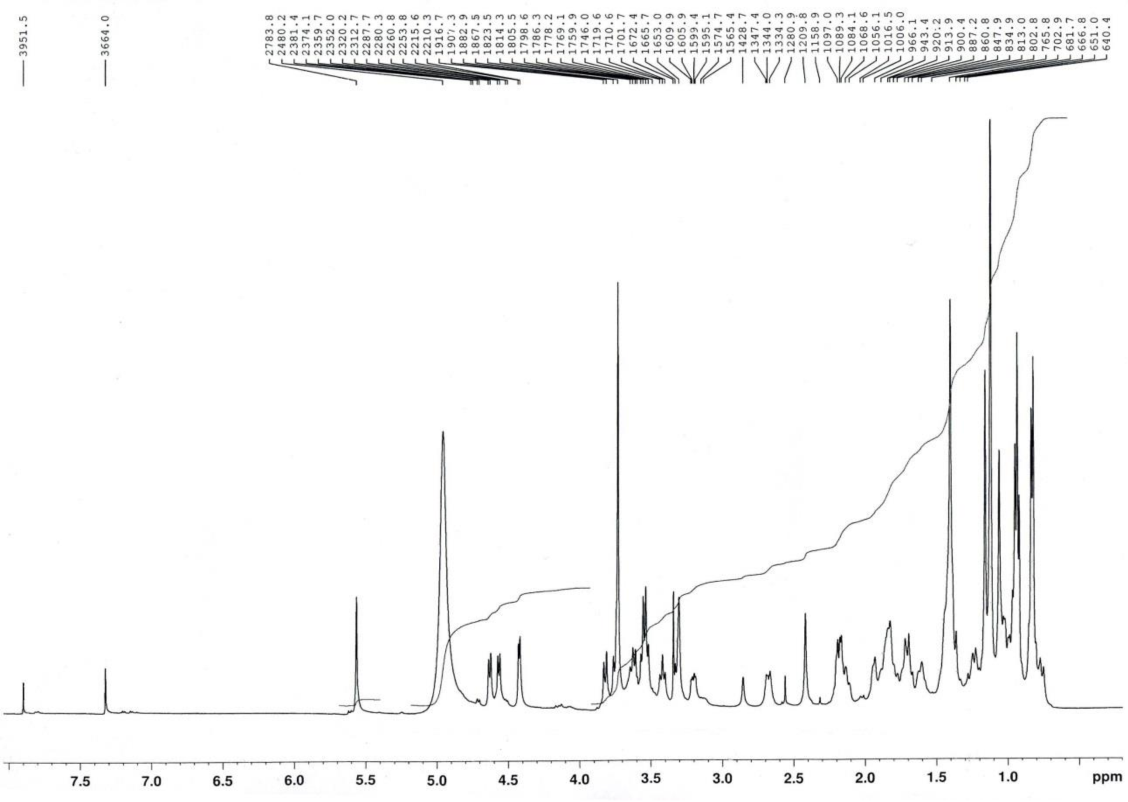

Supplemental Figure S1. ^{1}H NMR for compound-lead **3** (500 MHz, CD_3OD, δ, ppm)

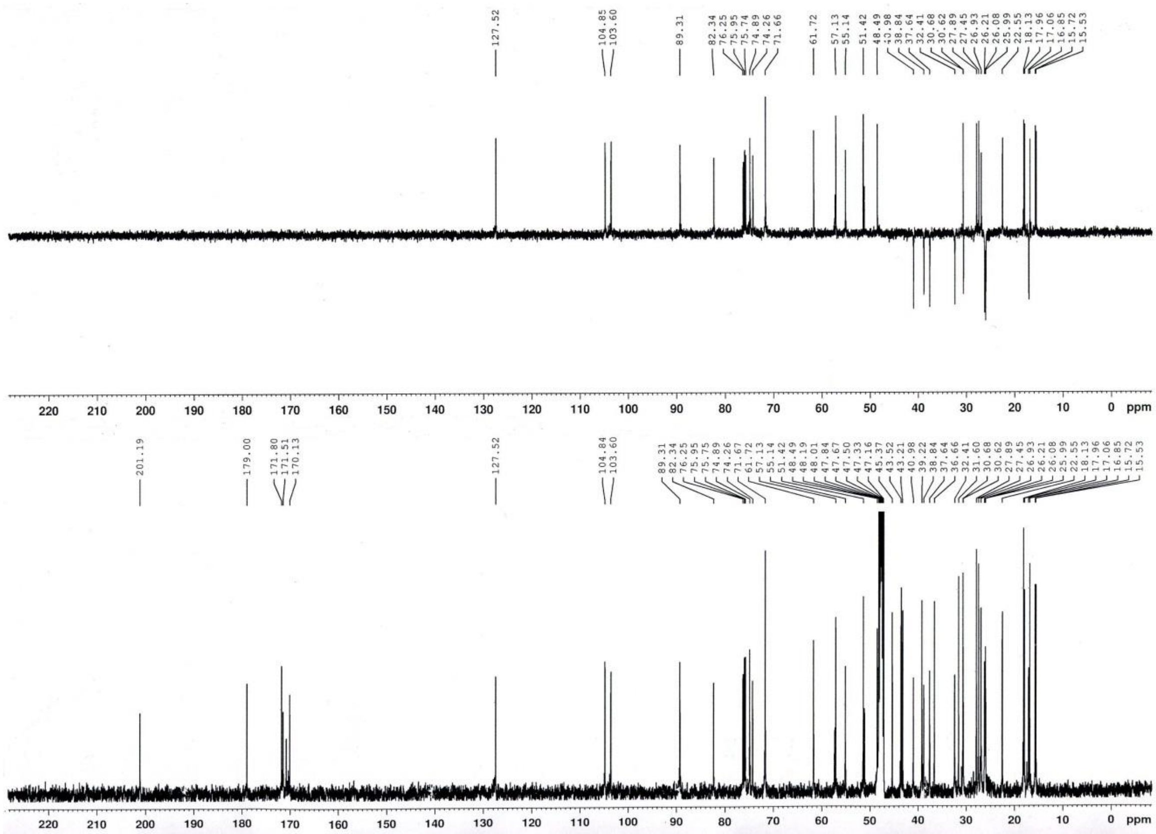

Supplemental Figure S2. ^{13}C NMR for compound-lead **3** (125 MHz, CD_3OD, δ, ppm)

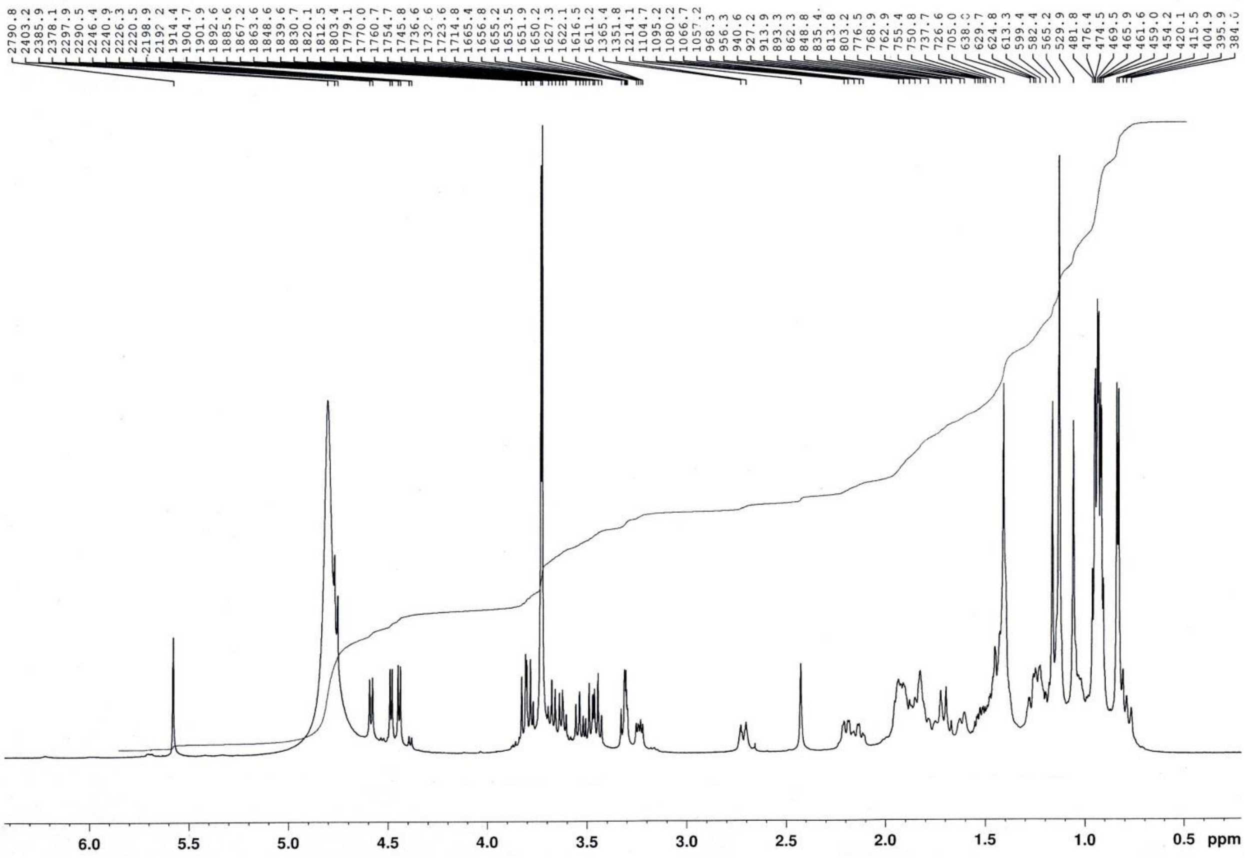

Supplemental Figure S3. ^{1}H NMR for compound **4** (500 MHz, CD_3OD, δ, ppm)

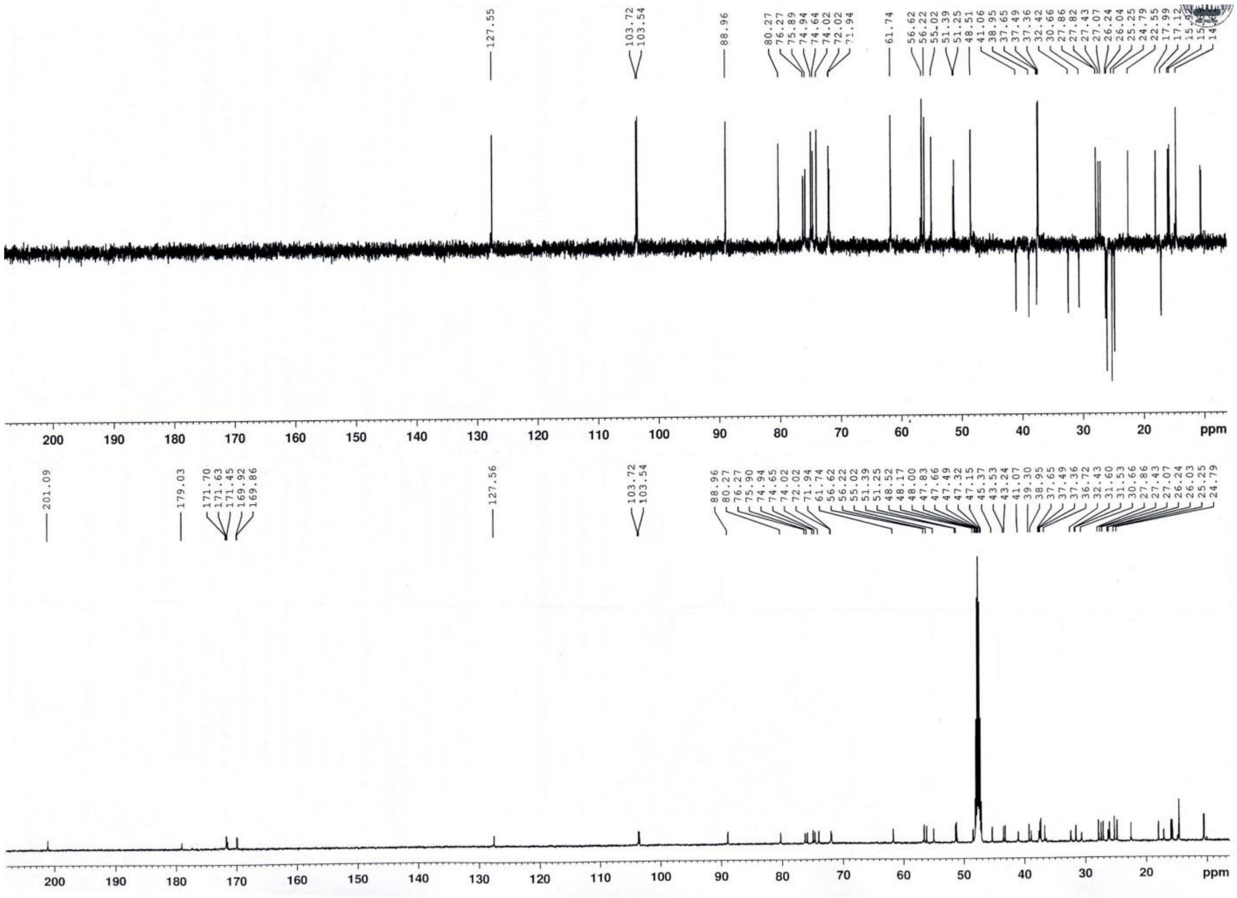

Supplemental Figure S4. ^{13}C NMR for compound **4** (125 MHz, CD_3OD, δ, ppm)

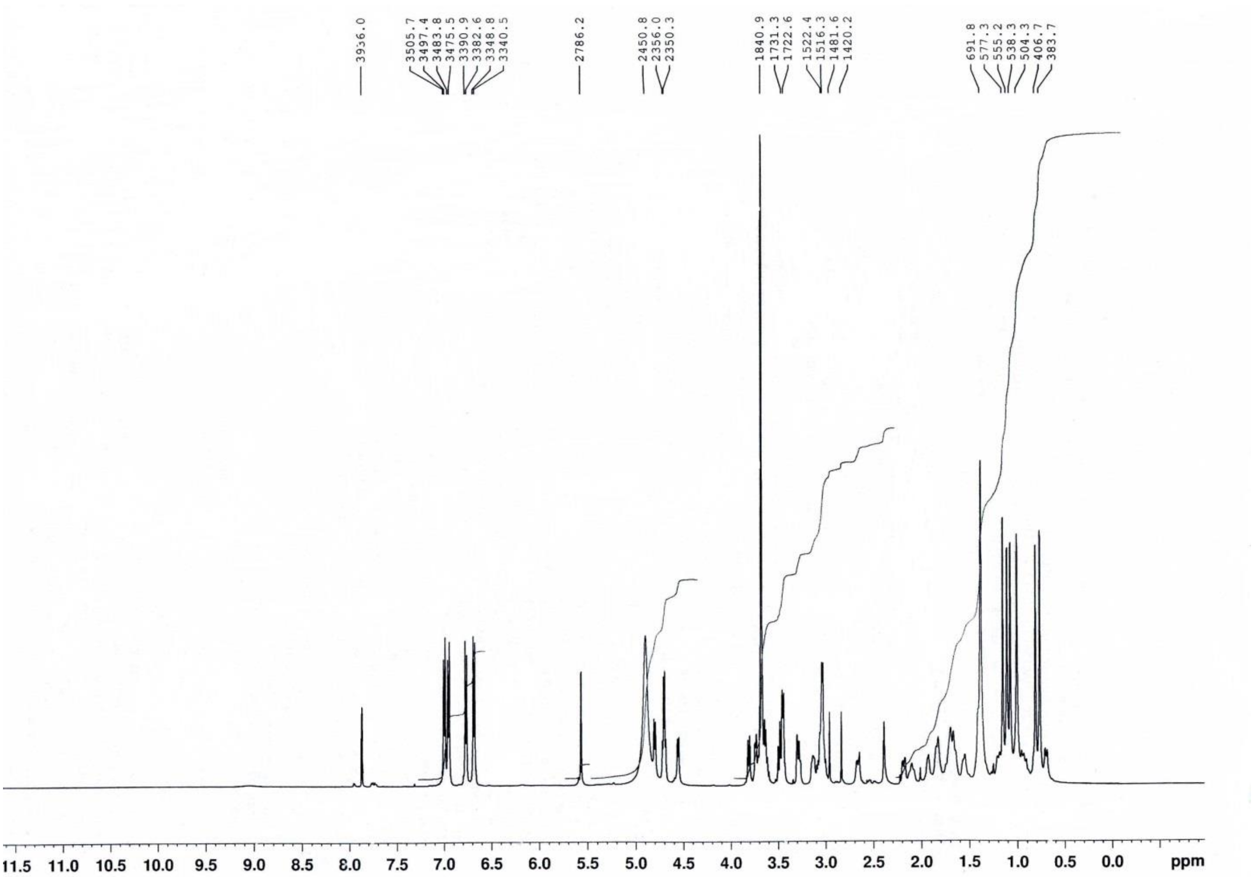

Supplemental Figure S5. ^{1}H NMR for compound-lead **6** (500 MHz, CD_3OD, δ, ppm)

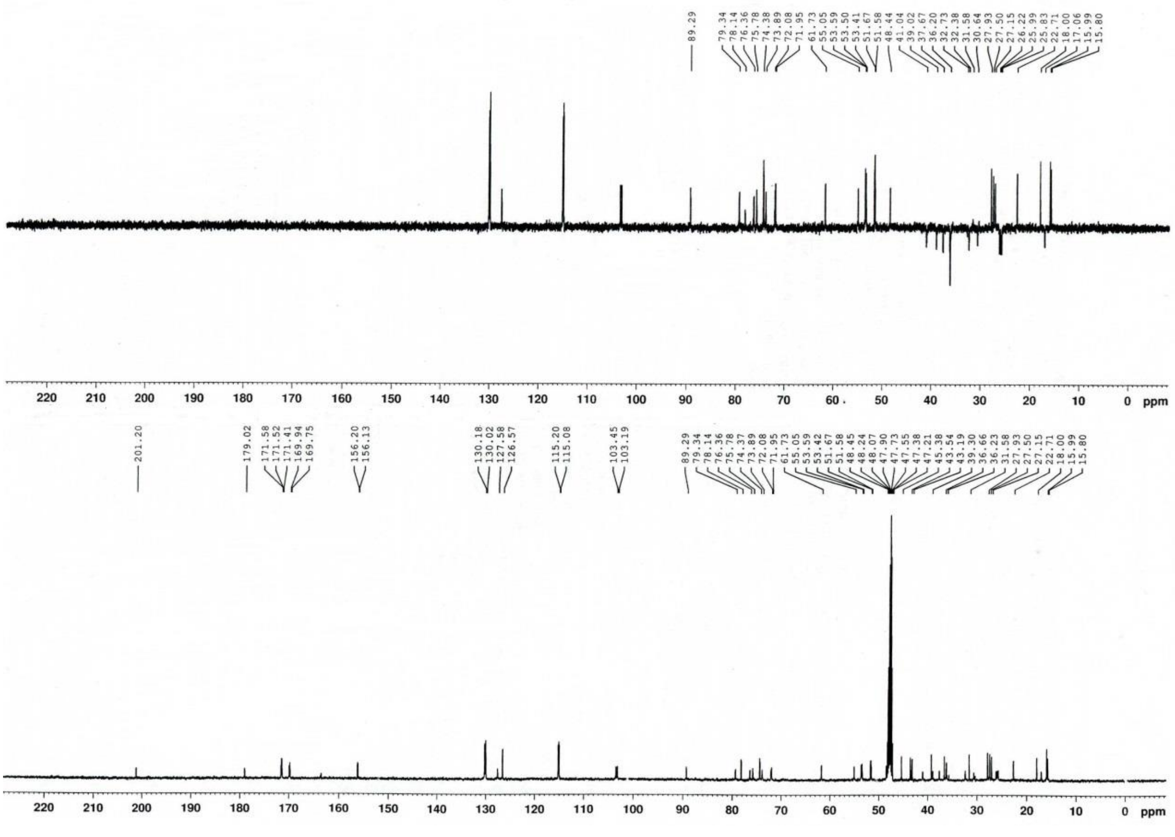

Supplemental Figure S6. ^{13}C NMR for compound-lead **6** (125 MHz, CD_3OD, δ, ppm)

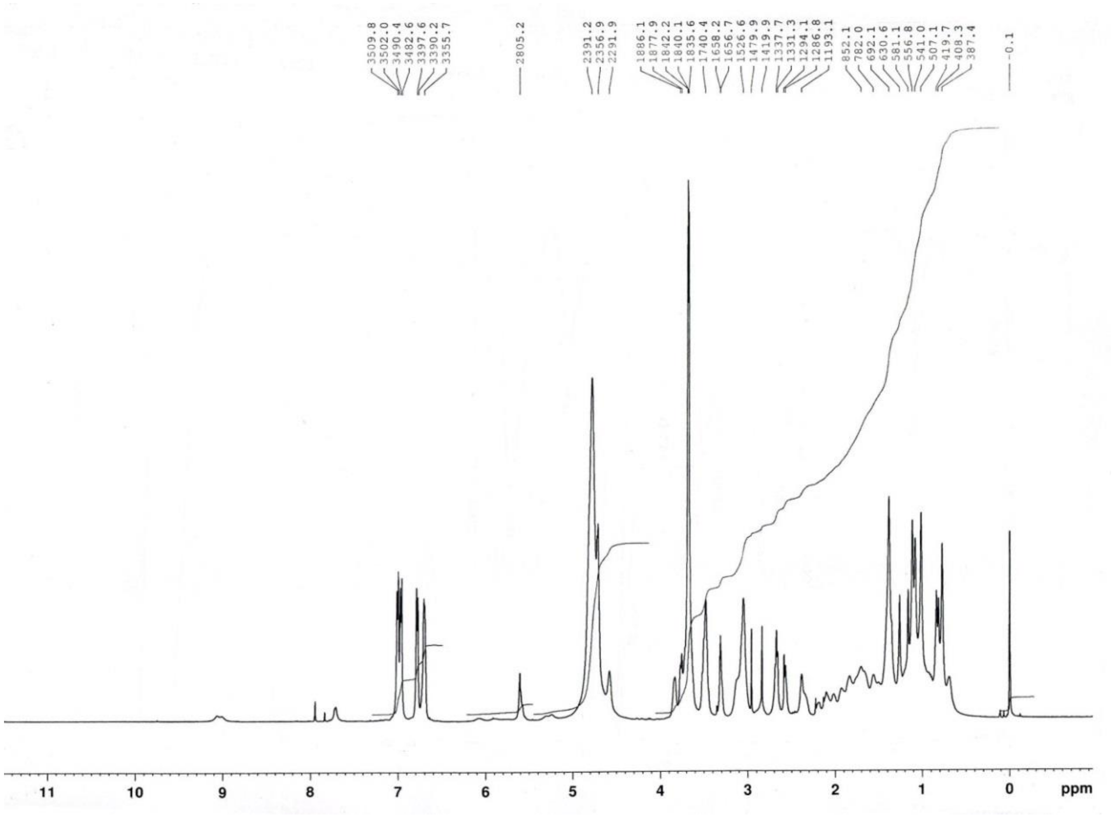

Supplemental Figure S7. ^{1}H NMR for compound **7** (500 MHz, CD_3OD, δ, ppm)

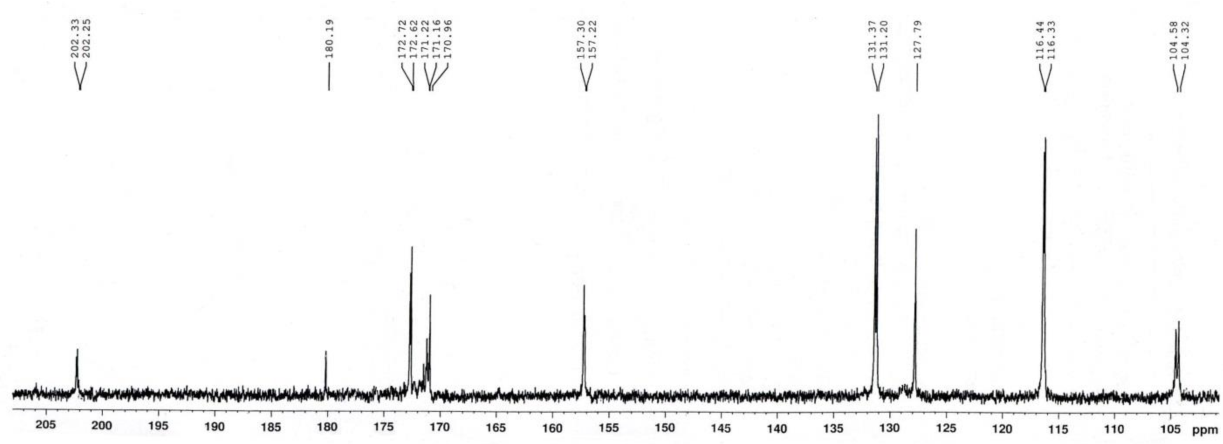

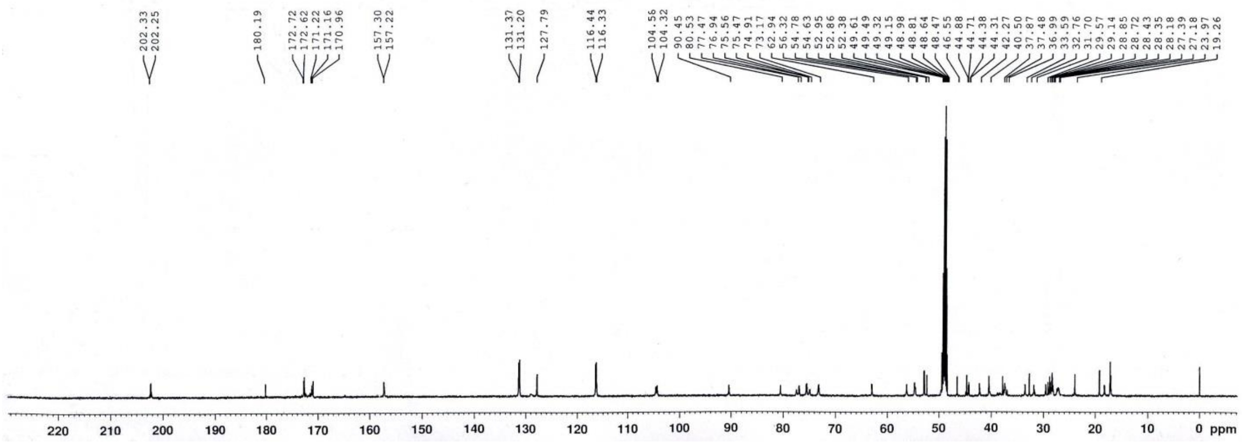

Supplemental Figure S8. ^{13}C NMR for compound **7** (125 MHz, CD_3OD, δ, ppm)

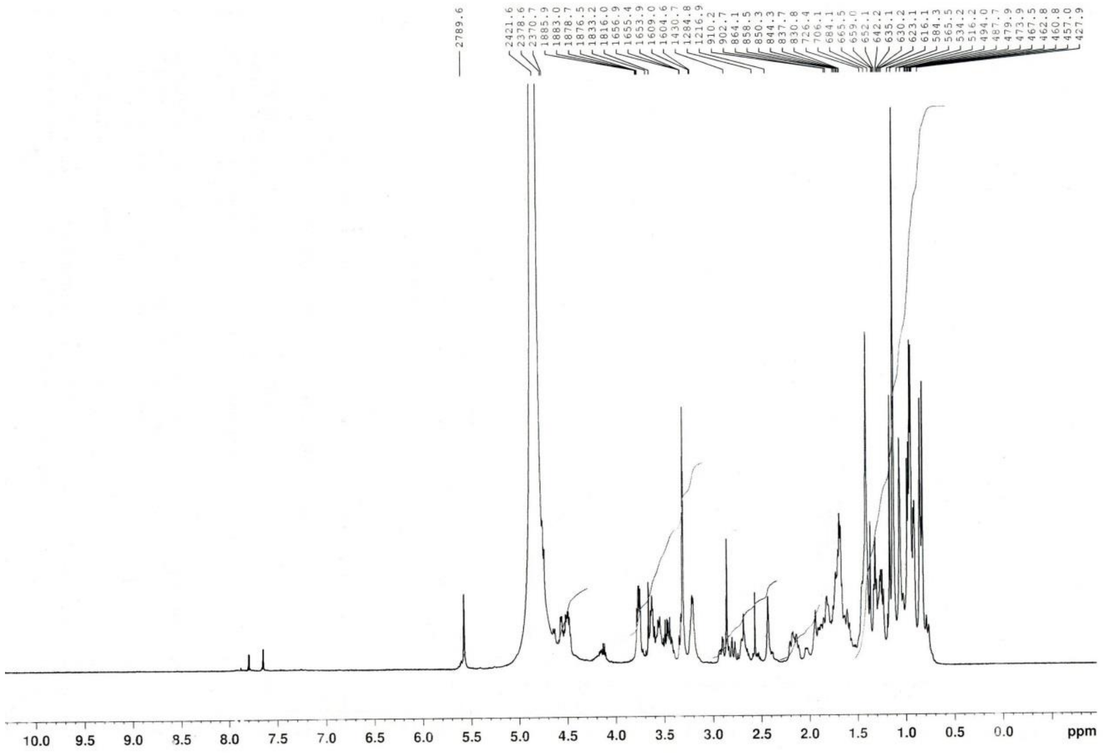

Supplemental Figure S9. ^{1}H NMR for compound **10** (500 MHz, CD_3OD, δ, ppm)

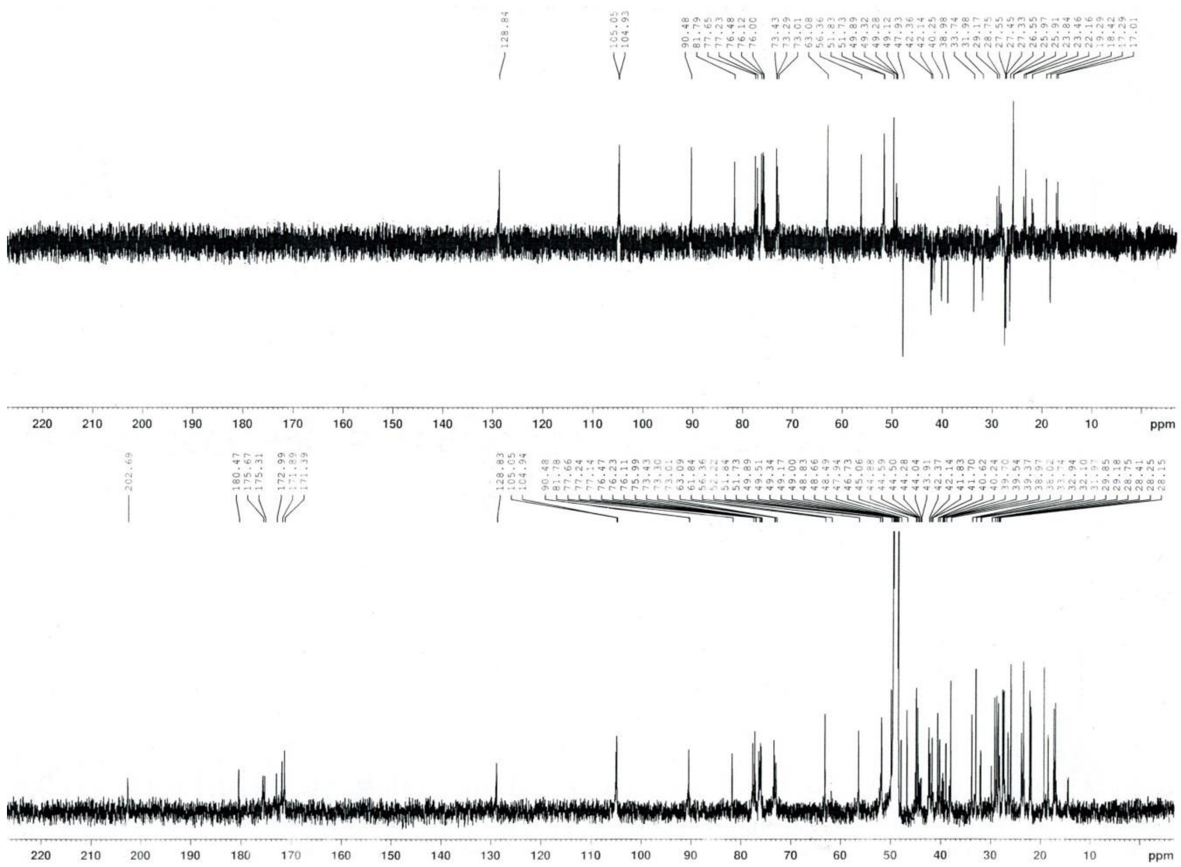

Supplemental Figure S10. ^{13}C NMR for compound **10** (125 MHz, CD_3OD, δ, ppm)

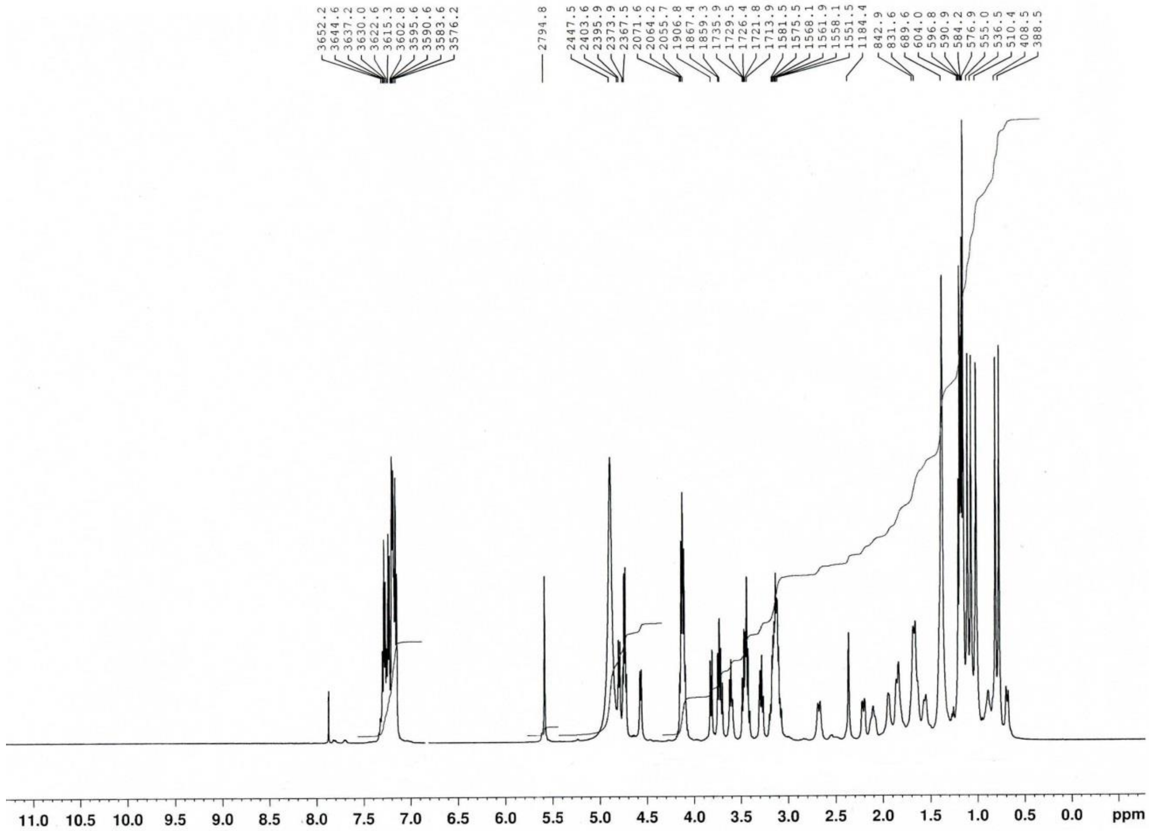

Supplemental Figure S11. ^{1}H NMR for compound-lead **11** (500 MHz, CD_3OD, δ, ppm)

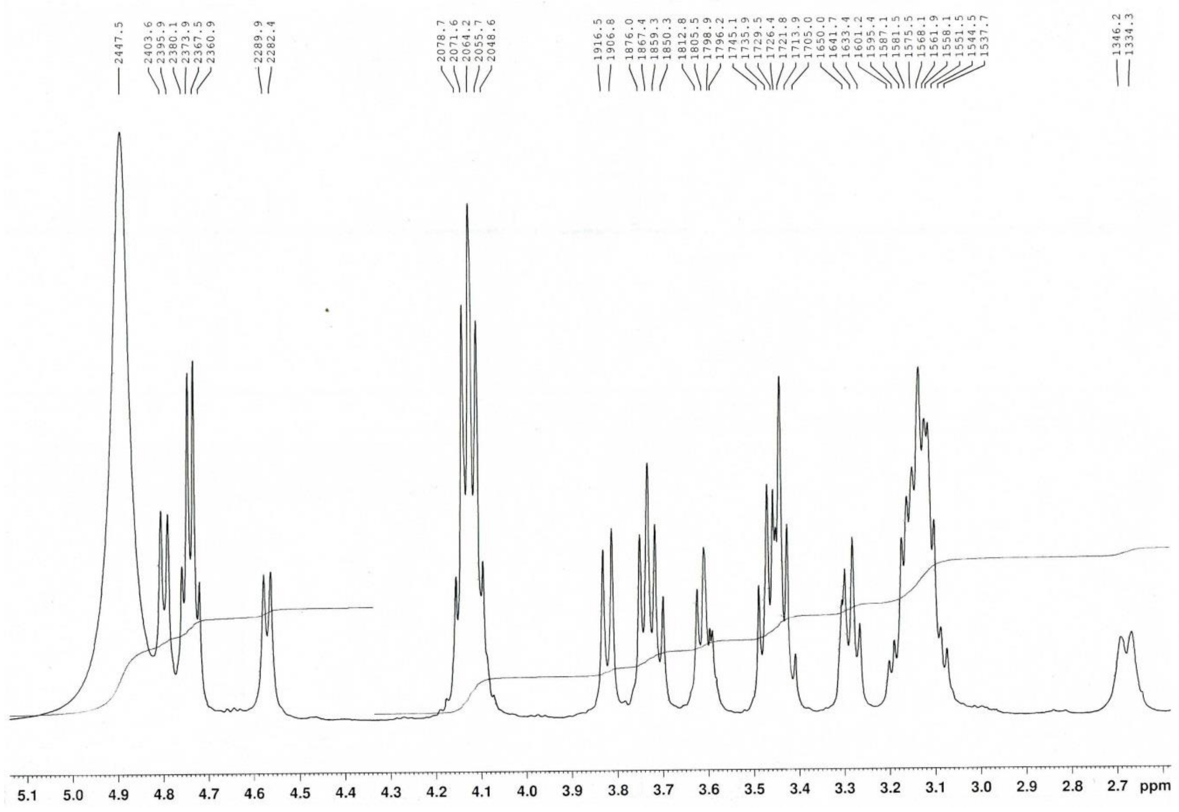

Supplemental Figure S12. A part of ^{1}H NMR for compound-lead **11** (500 MHz, CD_3OD, δ, ppm)

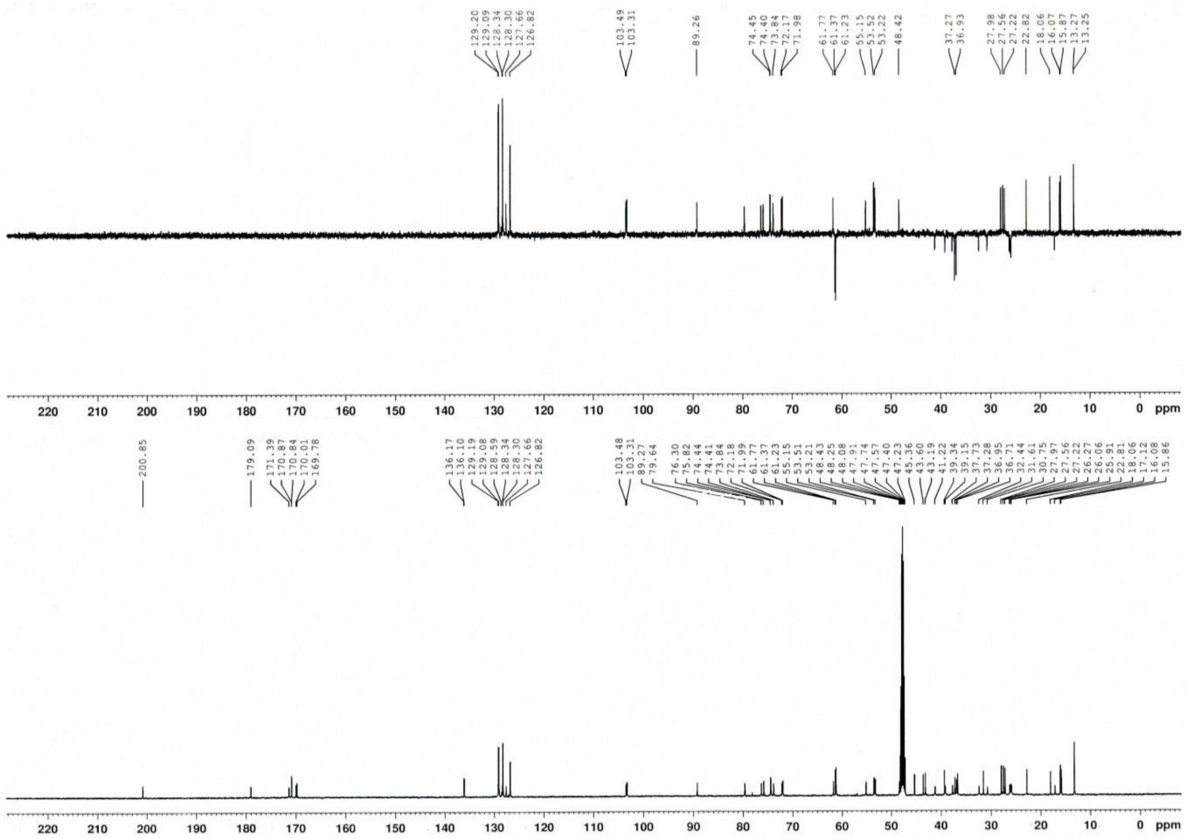

Supplemental Figure S13. ^{13}C NMR for compound-lead **11** (125 MHz, CD_3OD, δ, ppm)

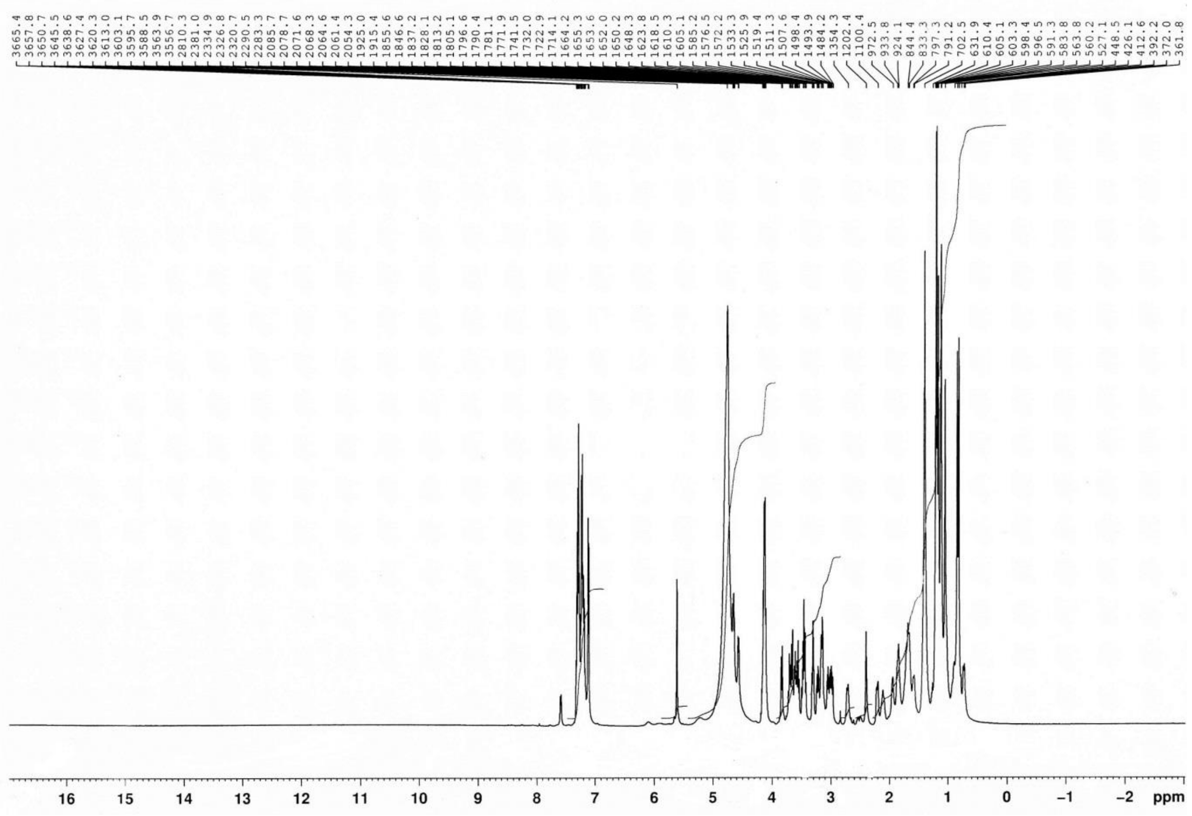

Supplemental Figure S14. ^{1}H NMR for compound **12** (500 MHz, CD_3OD, δ, ppm)

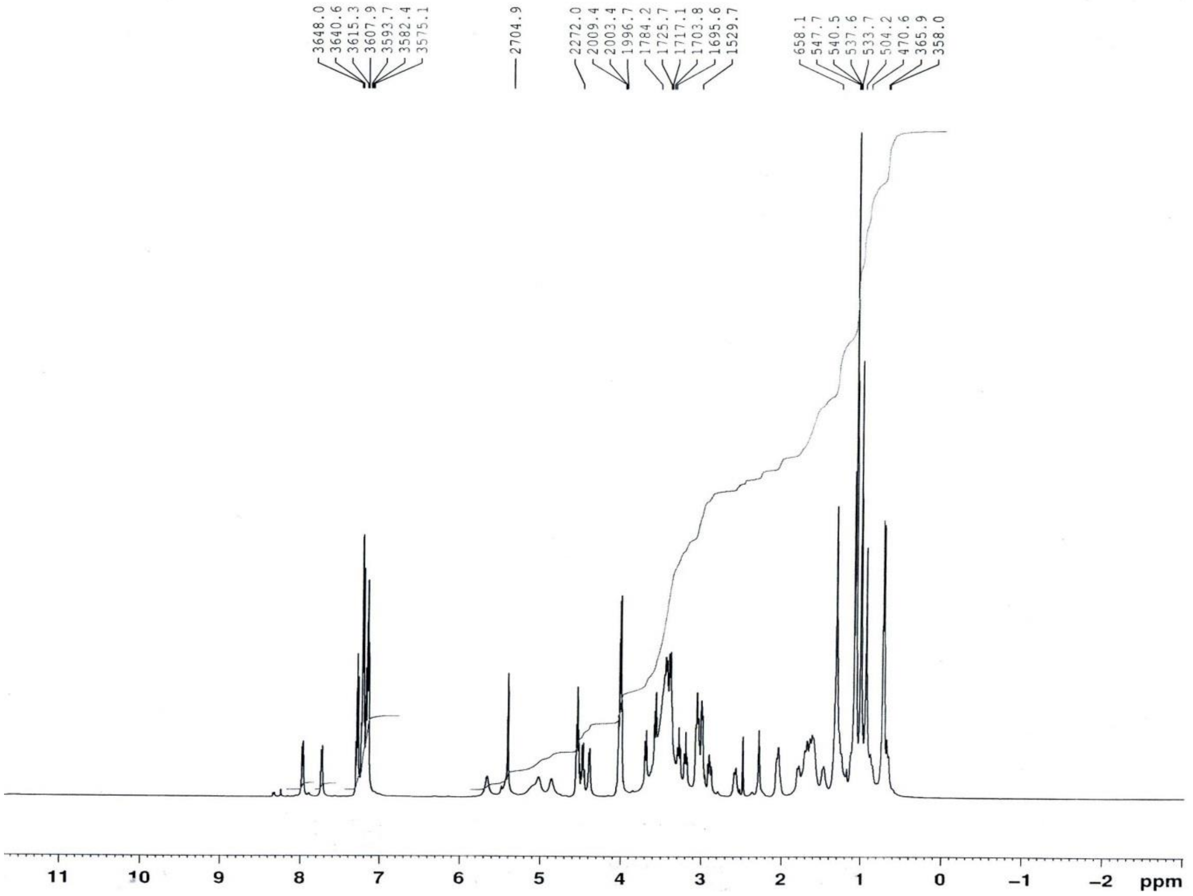

Supplemental Figure S15. ^{1}H NMR for compound **12** (500 MHz, DMSO-d_6, δ, ppm)

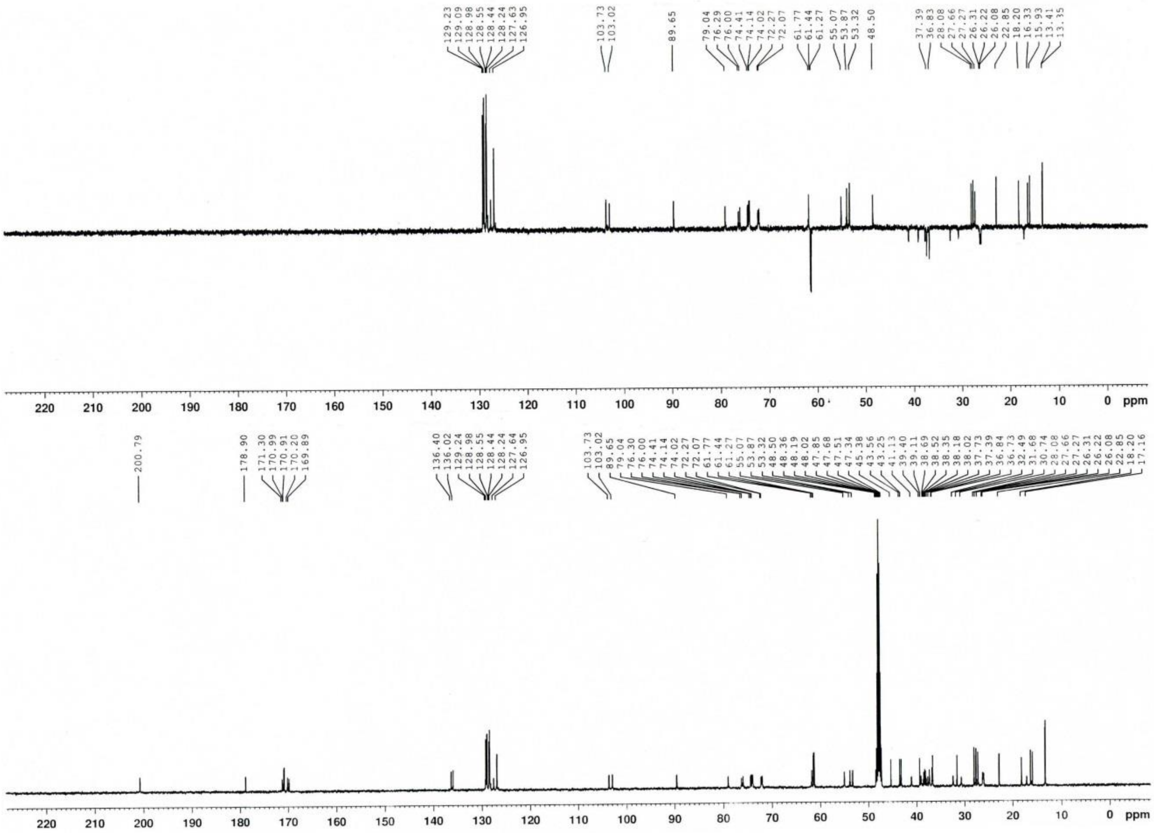

Supplemental Figure S16 . ^{13}C NMR for compound **12** (125 MHz, CD_3OD, δ, ppm)

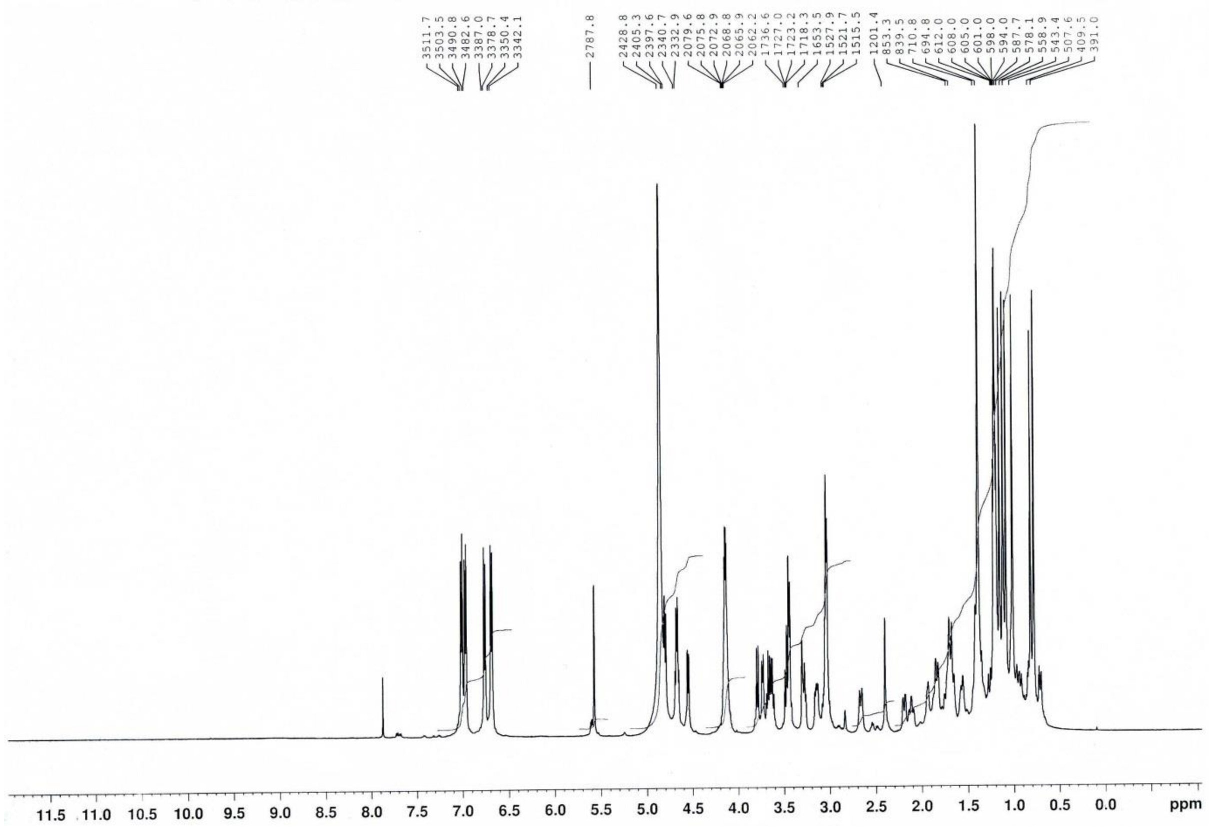

Supplemental Figure S17. ^{1}H NMR for compound **13** (500 MHz, CD_3OD, δ, ppm)

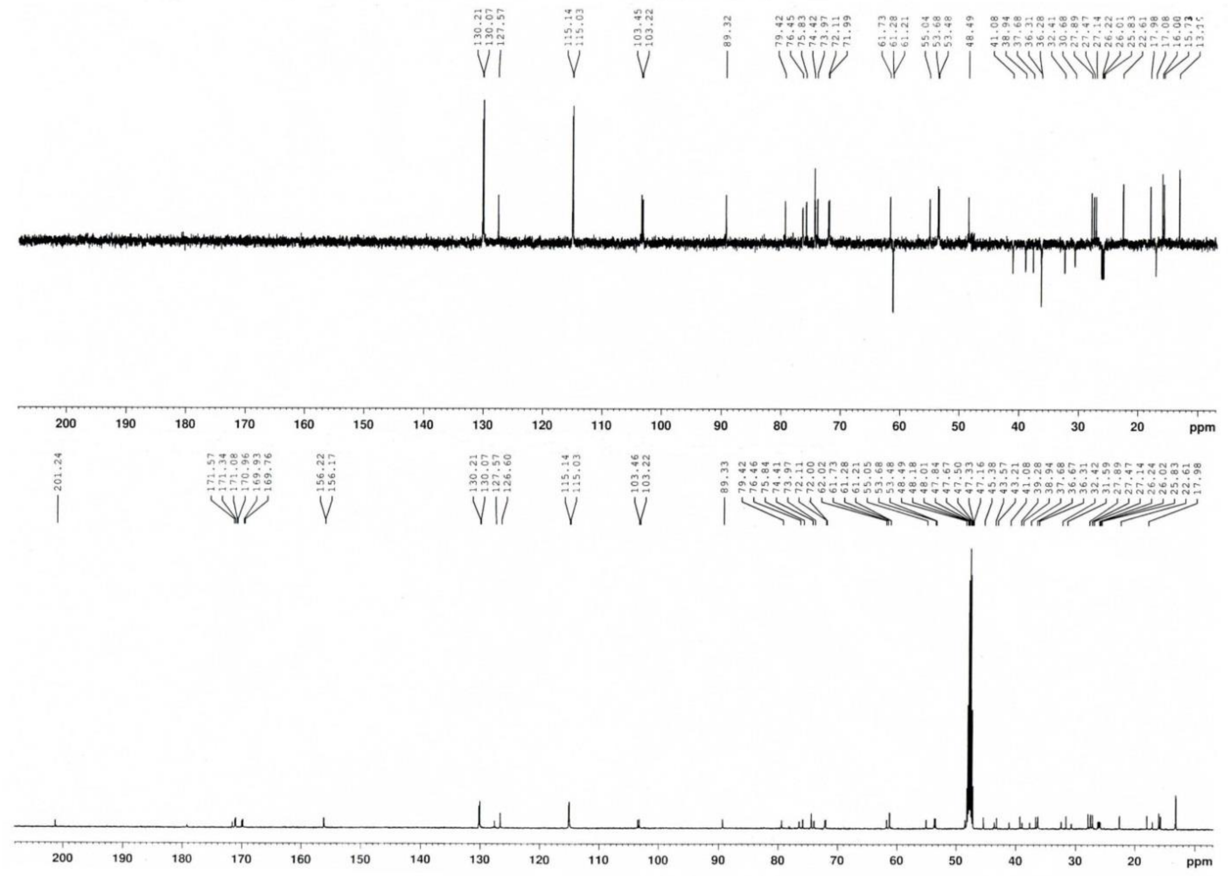

Supplemental Figure S18. ^{13}C NMR for compound **13** (125 MHz, CD_3OD, δ, ppm)

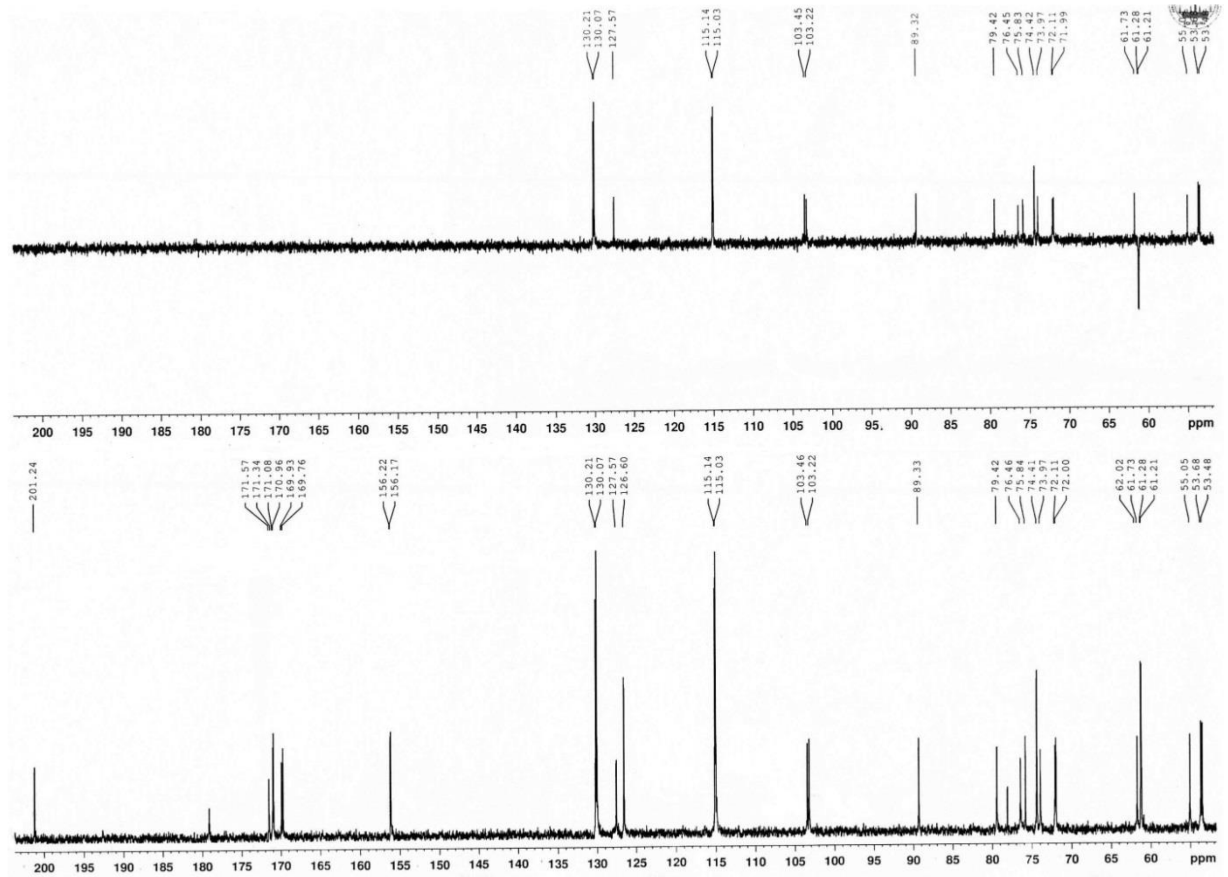

Supplemental Figure S19. ^{13}C NMR-2 for compound **13** (125 MHz, CD_3OD, δ, ppm)

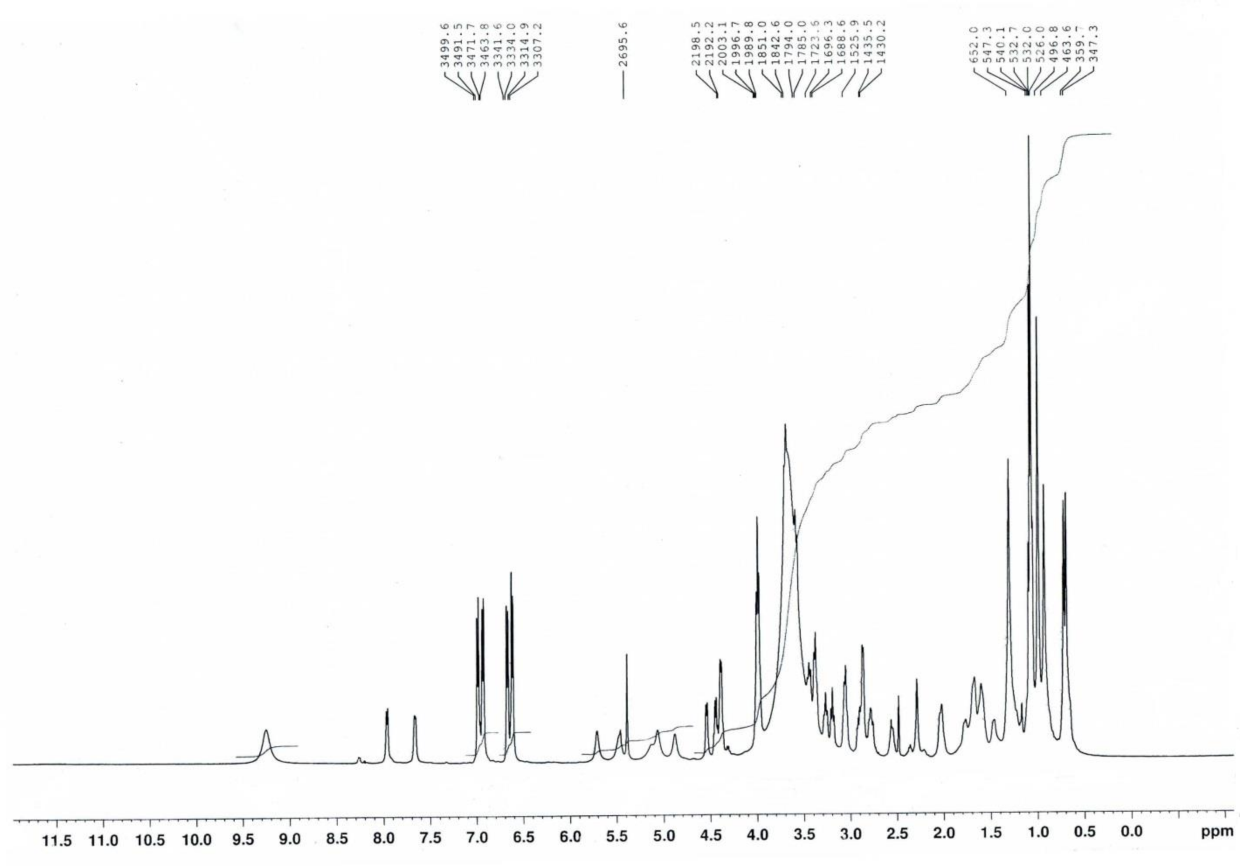

Supplemental Figure S20. ^{1}H NMR for compound **14** (500 MHz, DMSO-d_6, δ, ppm)

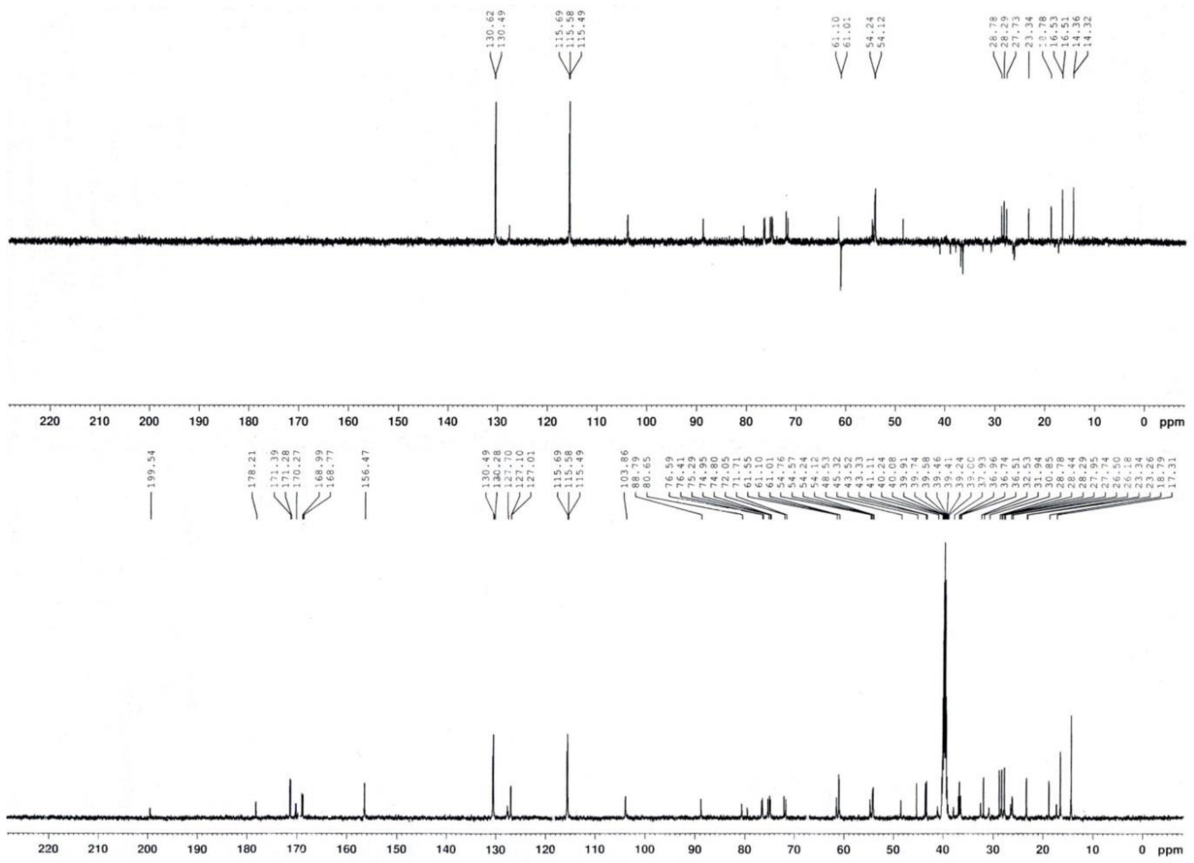

Supplemental Figure S21. ^{13}C NMR for compound **14** (125 MHz, DMSO-d_6, δ, ppm)

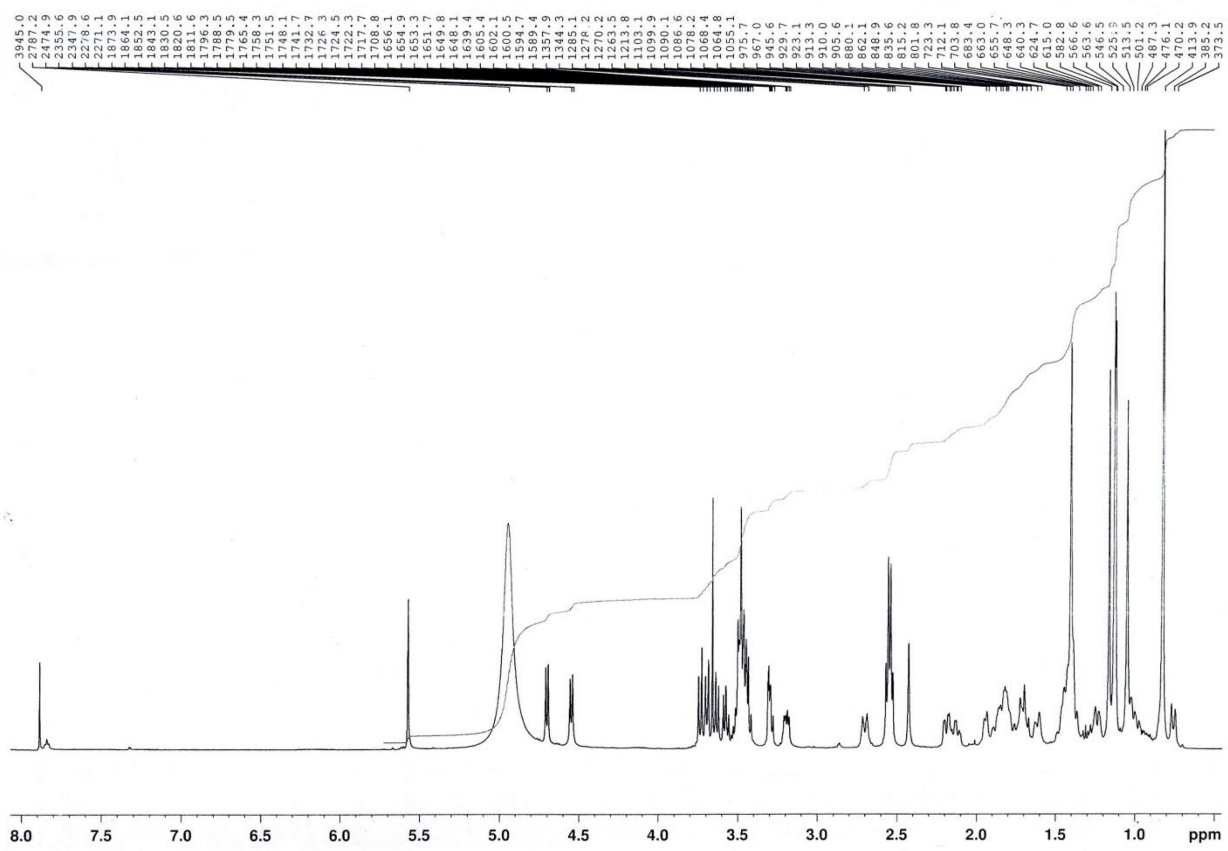

Supplemental Figure S22. ^{1}H NMR for compound **15** (500 MHz, CD_3OD, δ, ppm)

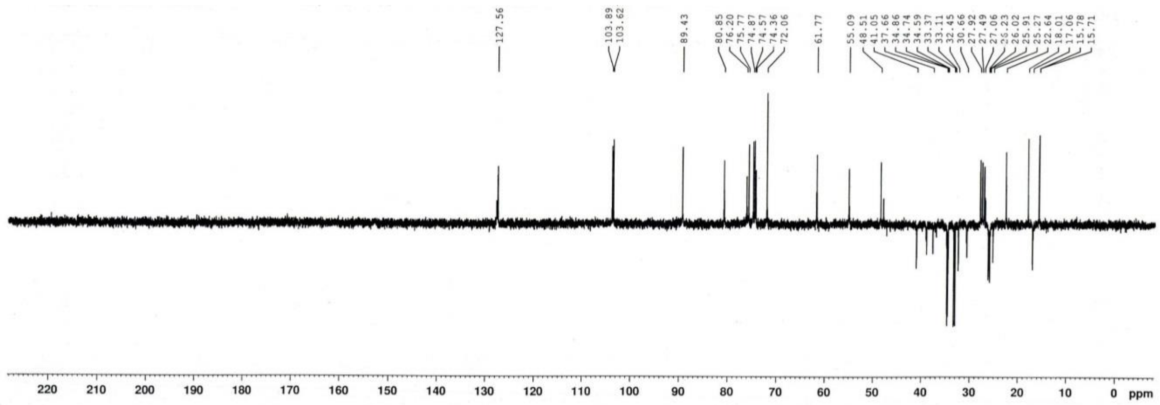

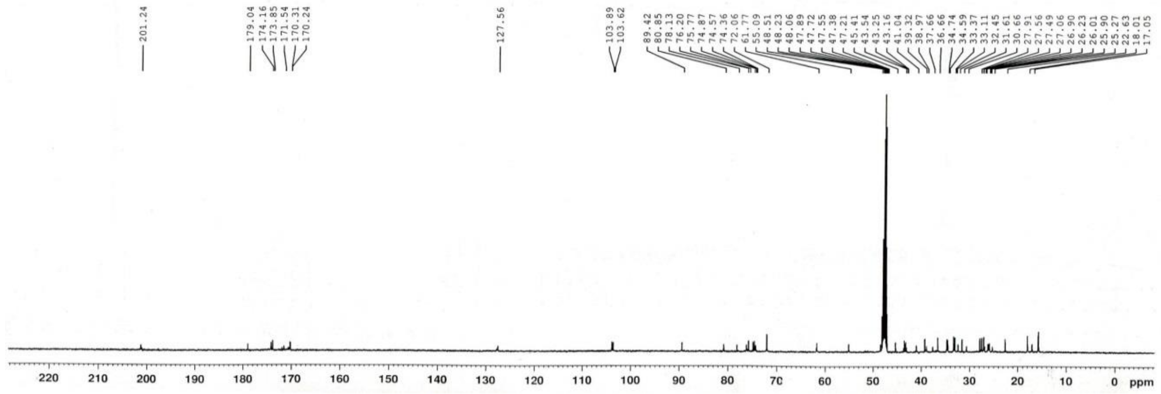

Supplemental Figure S23. ^{13}C NMR for compound **15** (125 MHz, CD_3OD, δ, ppm)

Part II: Antiviral data

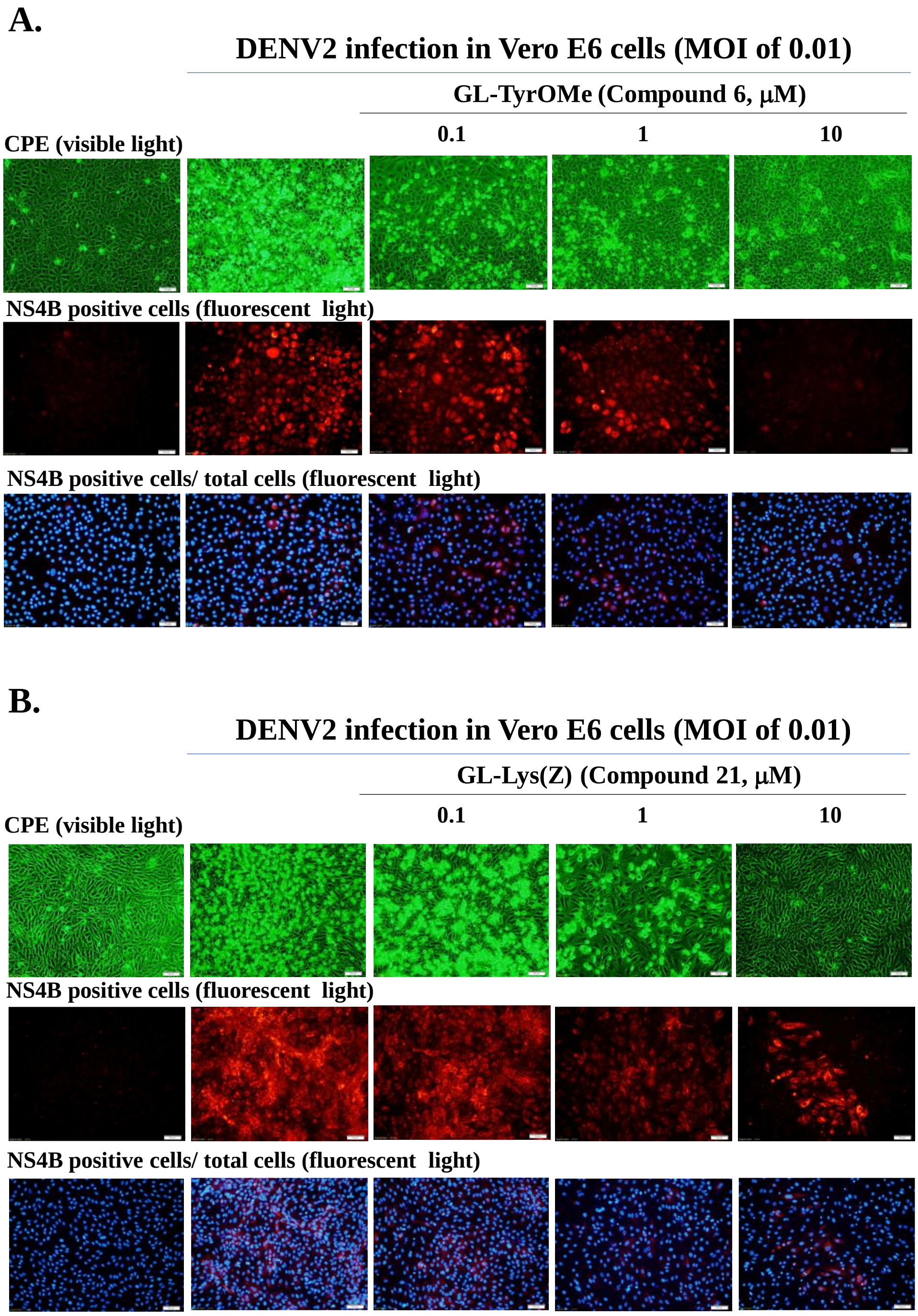

Supplemental Figure S24. Inhibition of DENV2-induced cytopathic effects and DENV2 protein expression by Compounds **6** and **21** in Vero E6 cells. Cells were infected with DENV2 at a MOI of 0.01 and immediately treated with the indicated concentrations of Compounds **6** and **21**. Images of DENV2-induced cytopathic effect were photographed 96 hours post infection by light microscopy (top). Treated/infected Vero E6 cells were also analyzed using immunofluorescence staining with anti-DENV2 NS4B antibodies. DENV2 infectivity was discovered by the ratio of DENV2 NS4B positive cells (middle) to total cells stained with DAPI (bottom).

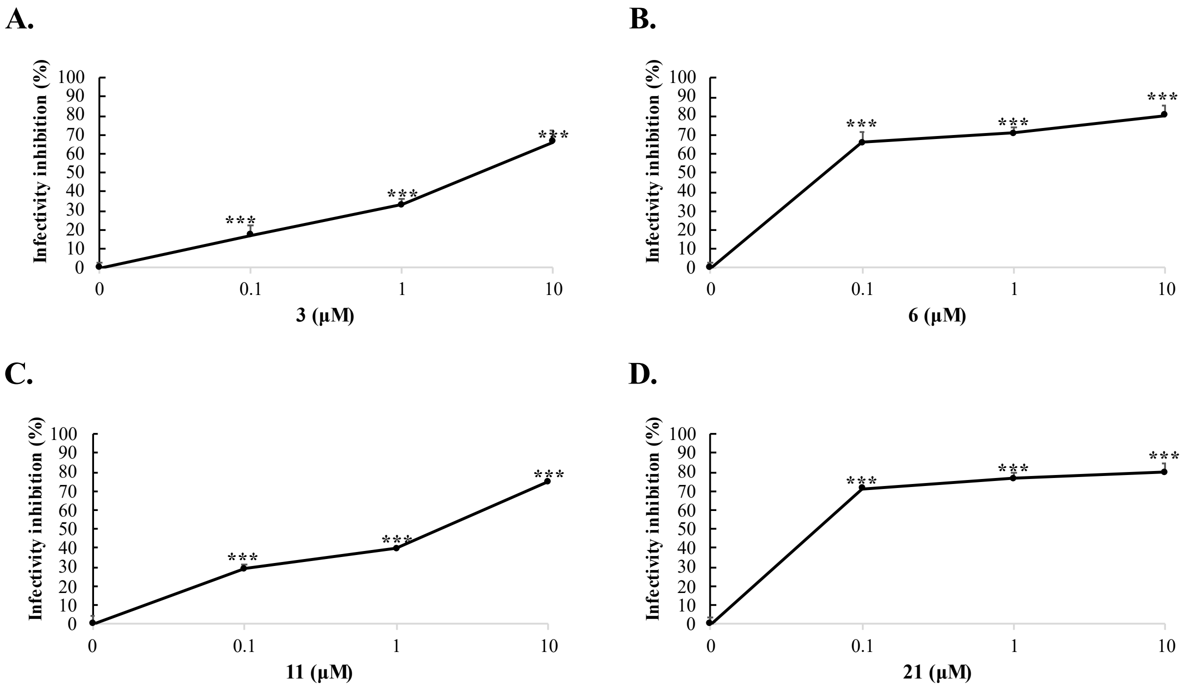

Supplemental Figure S25. Infectivity inhbition of DENV1 in Vero E6 cells by active GL conjugates. Cells were infected with DENV1 in the presence and absence of Compounds 3, 6, 11, and 21 at the concentrations of 0.1, 1, and 10 µM, respectively. Treated/infected Vero E6 cells were also analyzed using immunofluorescence staining with anti-DENV2 NS4B antibodies. Residual infectivity was discovered by the ratio of DENV2 NS4B positive cells to total cells stained with DAPI, and relative inhibition activity was determined based on full infectivity with the subtraction of its residual infectivity.

Part III: HPLC analysis for GL derivatives

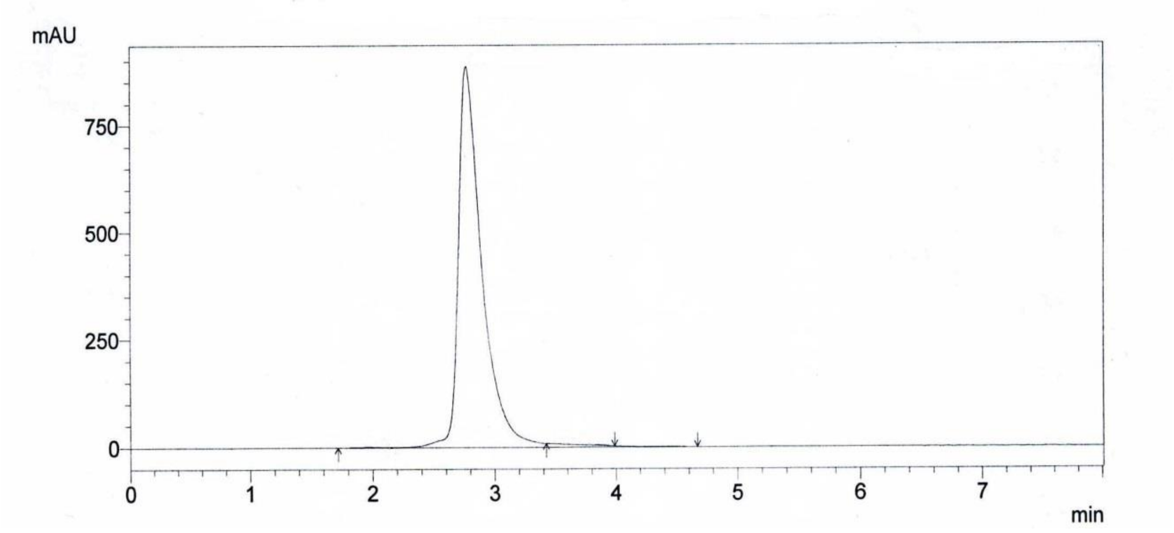

Supplemental Figure S26. HPLC for compound **2**. A Vydac 218 TPC18 column; a mobile phase CH_3OH; a flow rate 1.0 ml/min; a retention time was 2.77 min; UV detection was carried out at λ 254 nm; a purity was 97.8±0.8%.

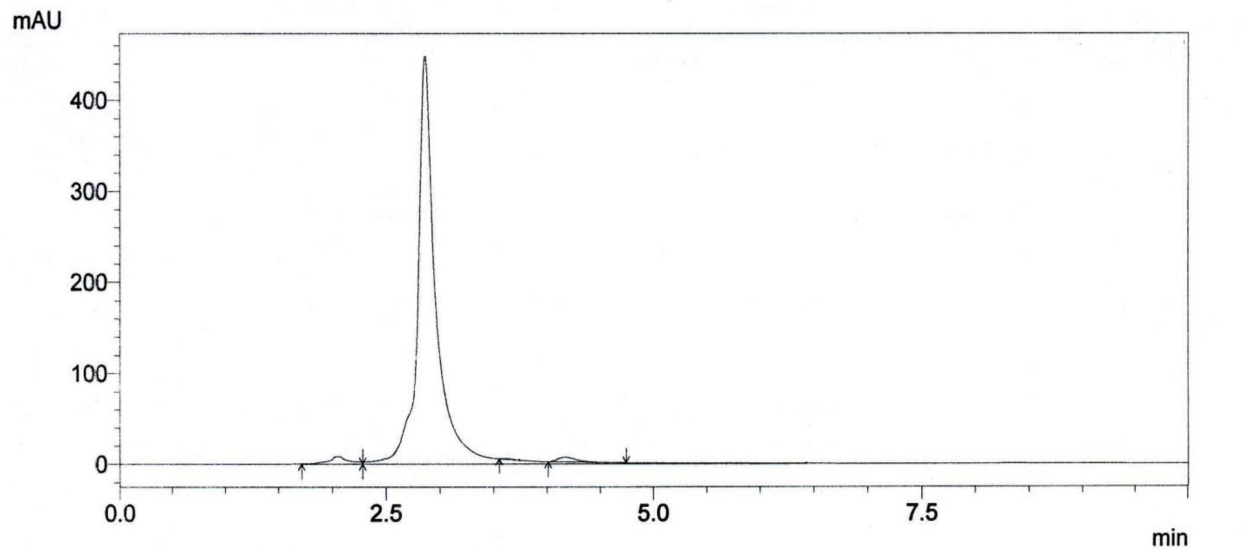

Supplemental Figure S27. HPLC for compound **3**. A Vydac 218 TPC18 column; a mobile phase CH_3OH; a flow rate 1.0 ml/min; a retention time was 2.86 min; UV detection was carried out at λ 254 nm; a purity was 96.8±0.8%.

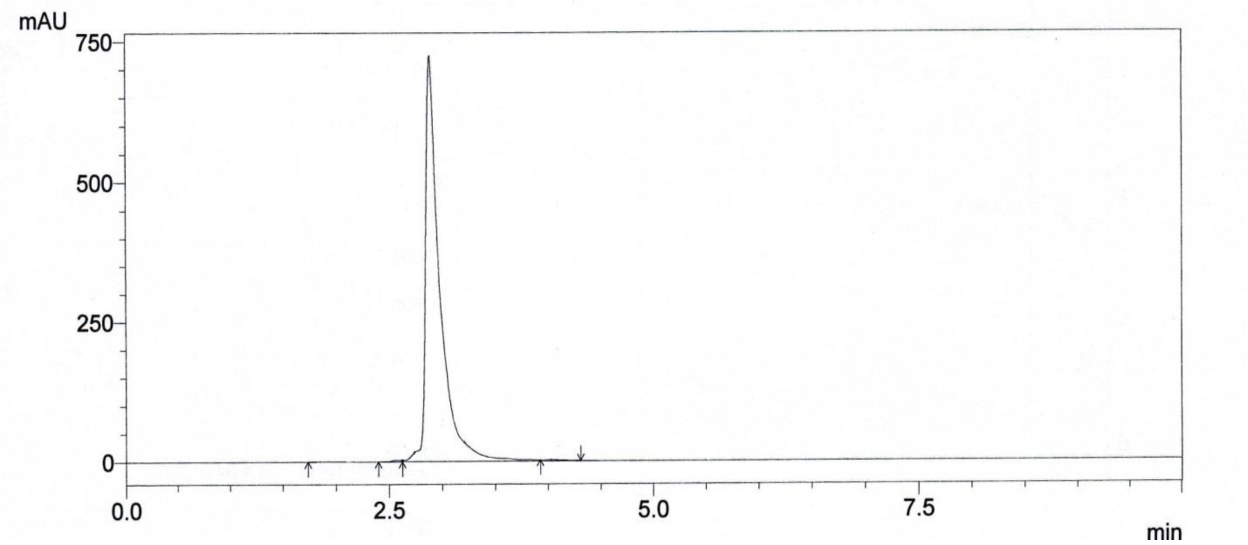

Supplemental Figure S28. HPLC for compound **4.** A Vydac 218 TPC18 column; a mobile phase CH_3OH; the flow rate 1.0 ml/min; a retention time was 2.88 min; UV detection was carried out at λ 254 nm; a purity was 98.2±0.8%.

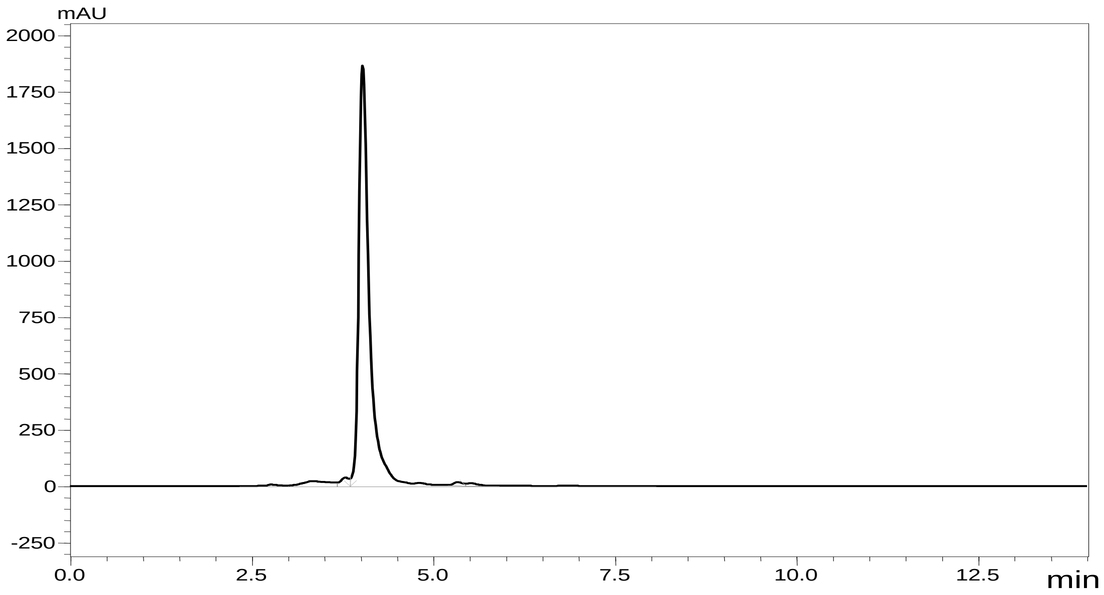

Supplemental Figure S29. HPLC for compound **5.** A Discovery C18 column; a mobile phase CH_3OH; a flow rate 1.0 ml/min; a retention time 4.03 min; UV detection was carried out at λ 254 nm; a purity was 97.5±0.8%.

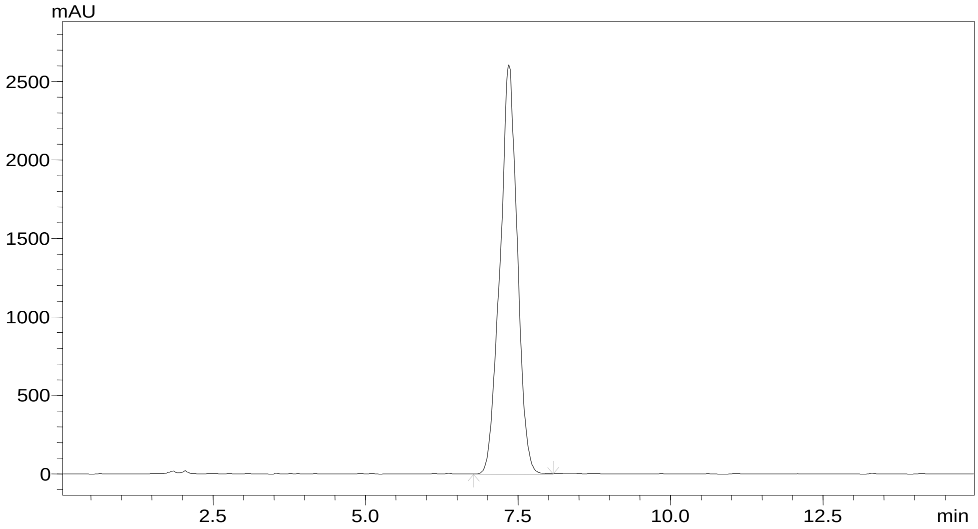

Supplemental Figure S30. HPLC for a leader compound **6.** An Atlantis C18 column; a mobile phase CH_3OH: 0.3 N CH_3COOH=80:20; a flow rate 1.0 ml/min; a retention time 7.95 min; UV detection was carried out at λ 254 nm; a purity was 98.0±0.8%.

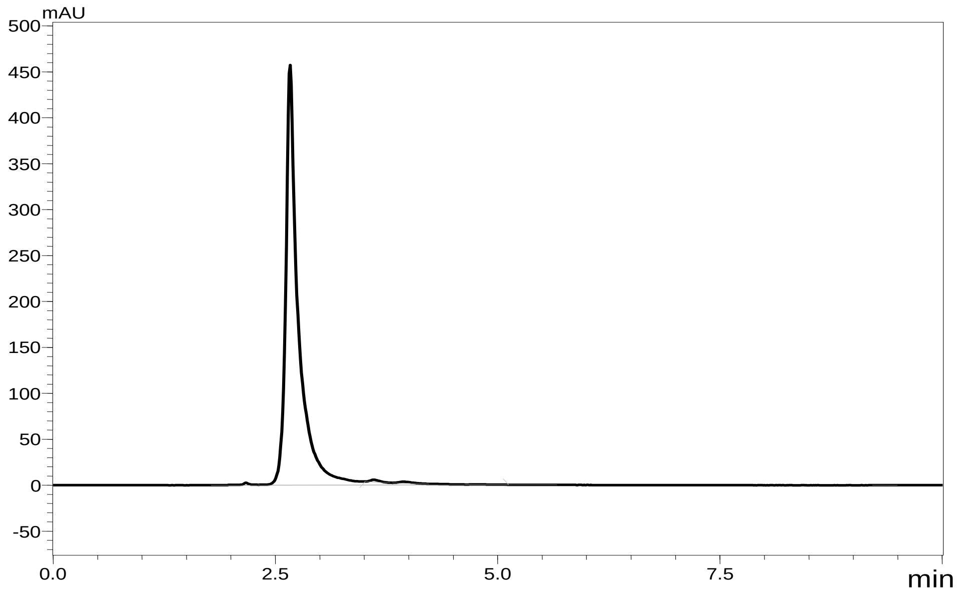

Supplemental Figure S31. HPLC for compound **7. A** Vydac 218 TPC18 column; a mobile phase was CH_3OH; a flow rate 1.0 ml/min; a retention time 2.66 min; UV detection was carried out at λ 254 nm; a purity was 98.5±0.8%.

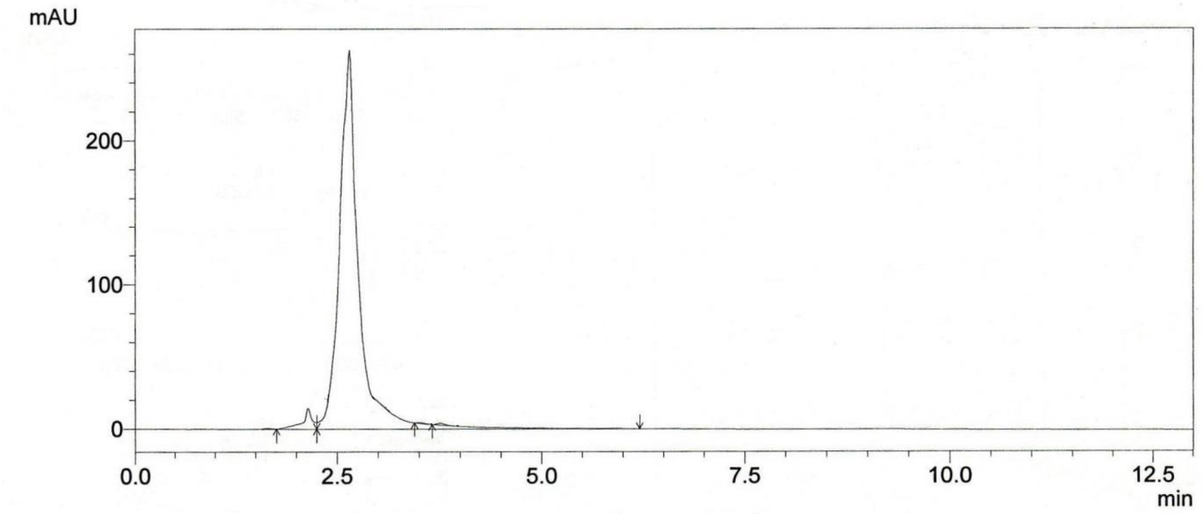

Supplemental Figure S32. HPLC for compound **8.** A Vydac 218 TPC18 column; a mobile phase was CH_3OH; a flow rate 1.0 ml/min; a retention time 2.65 min; UV detection was carried out at λ 254 nm; a purity was 95.8±0.8%.

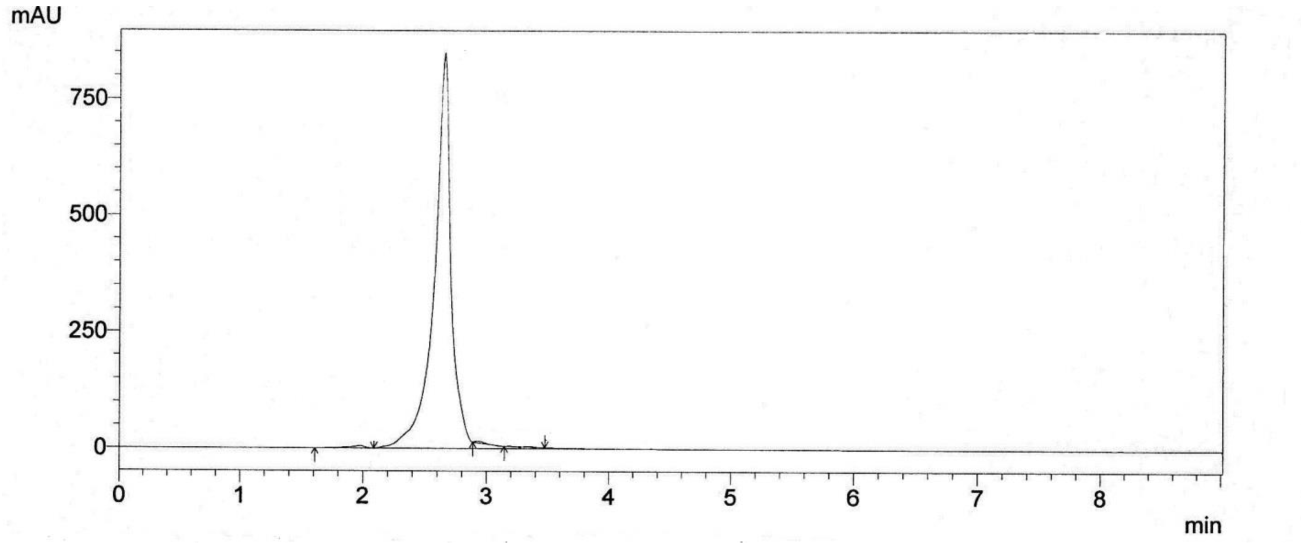

Supplemental Figure S33. HPLC for compound **9.** A Zorbax RX-C18 column; mobile phase CH_3OH; a flow rate 1.0 ml/min; a retention time 2.65 min; UV detection was carried out at λ 254 nm; a purity was 96.8±0.8%.

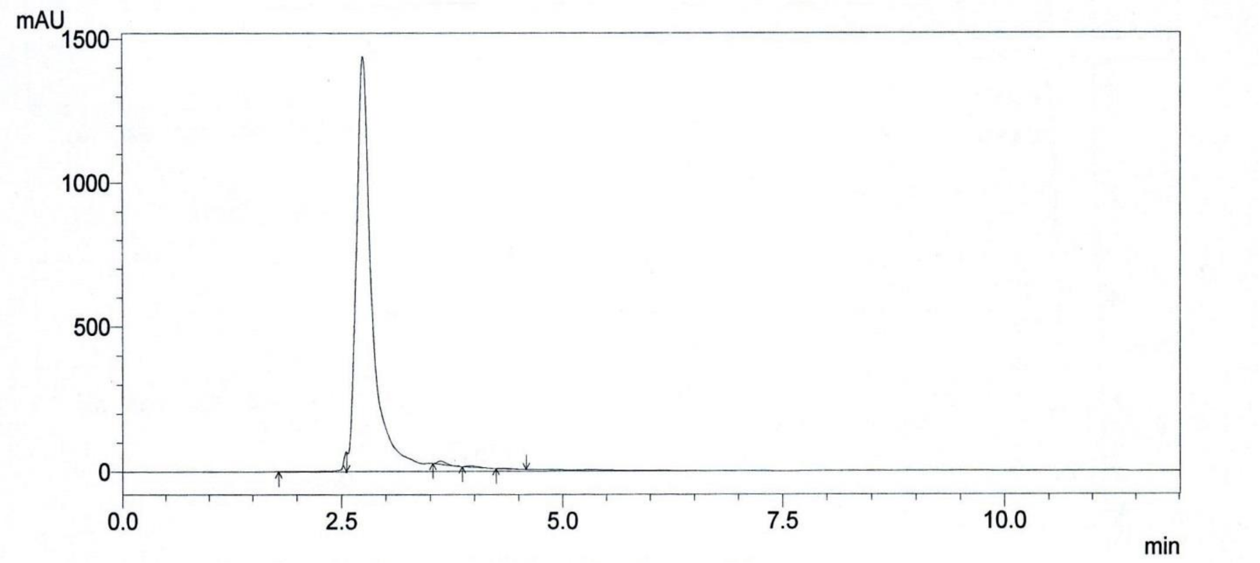

Supplemental Figure S34. HPLC for compound **10.** A Vydac 218 TPC18 column; a mobile phase CH_3OH; a flow rate 1.0 ml/min; a retention time 2.74 min; UV detection was carried out at λ 254 nm; a purity was 97.6±0.8%.

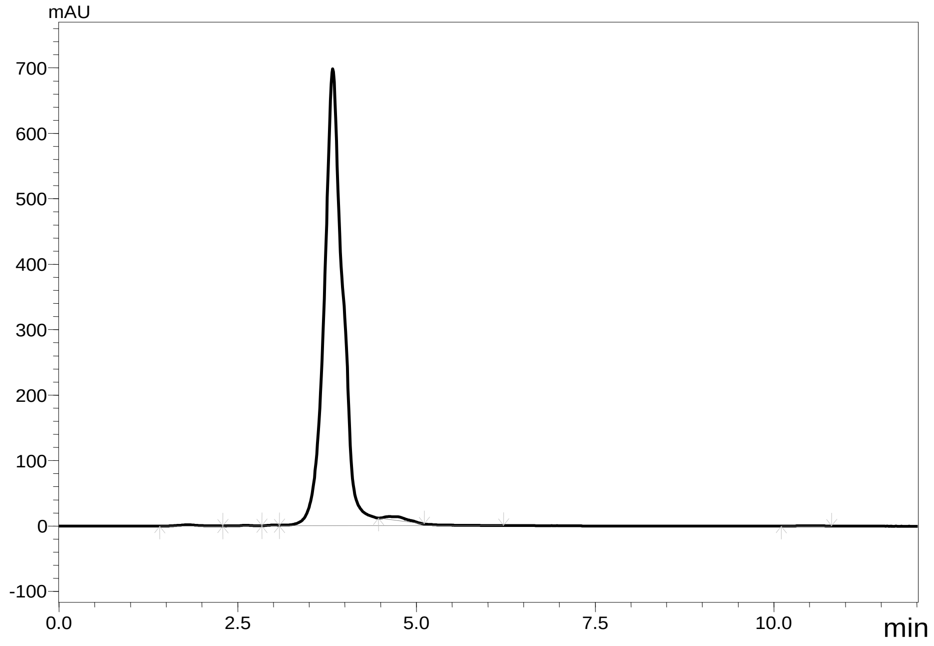

Supplemental Figure S35. HPLC for a leader compound **11.** An Atlantis C18 column; a mobile phase CH_3OH; a flow rate 1.0 ml/min; a retention time 3.82 min; UV detection was carried out at λ 254 nm; a purity was 96.6±0.8%.

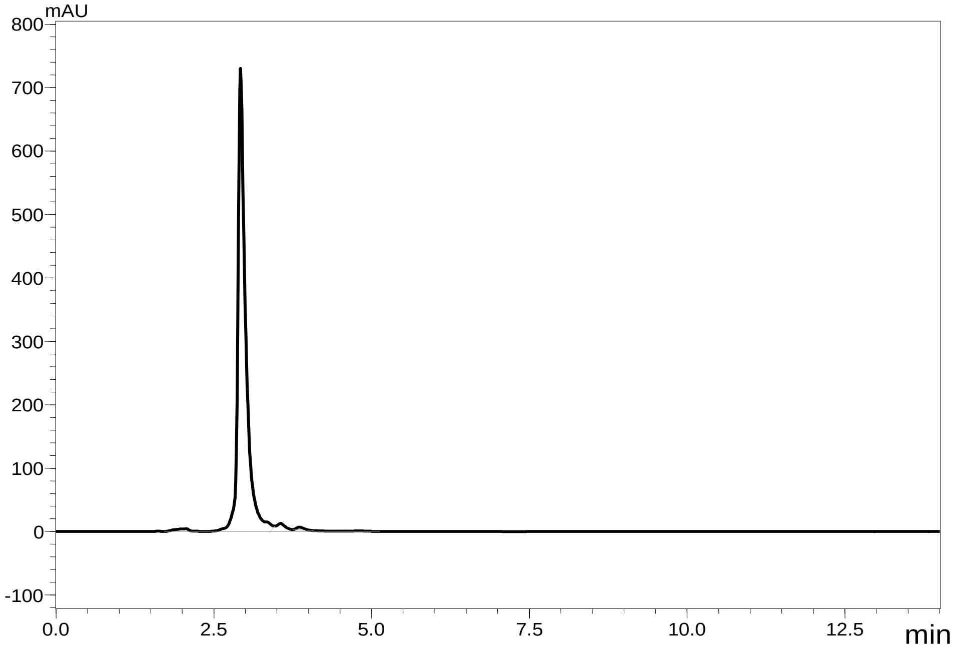

Supplemental Figure S36. HPLC for compound **12**. A Vydac 218 TPC18 column; a mobile phase CH_3OH; a flow rate 1.0 ml/min; a retention time 2.92 min; UV detection was carried out at λ 254 nm; a purity was 98.8±0.8%.

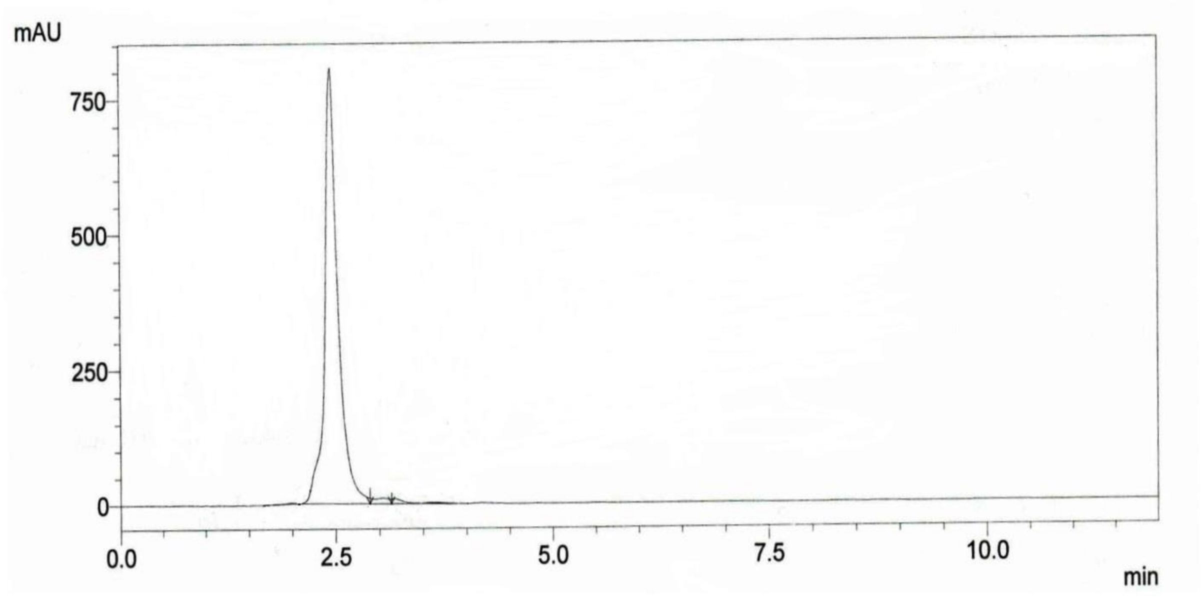

Supplemental Figure S37. HPLC for compound **13.** A Zorbax RX-C18 column; a mobile phase CH_3OH; a flow rate 1.0 ml/min; a retention time 2.54 min; UV detection was carried out at λ 254 nm; a purity was 97.6±0.8%.

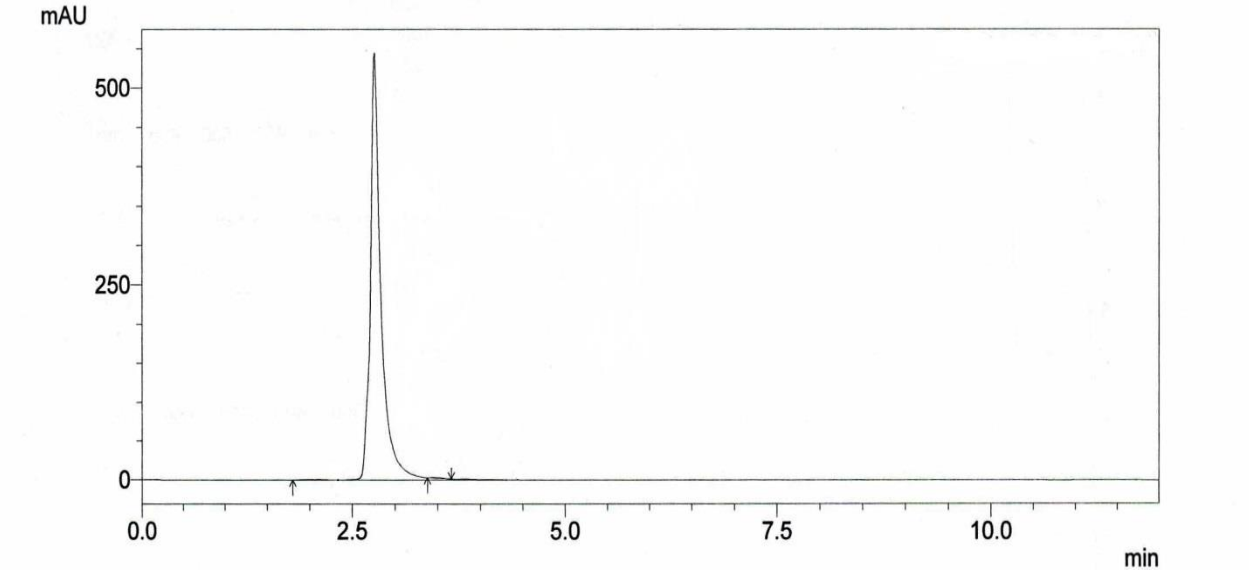

Supplemental Figure S38. HPLC for compound **14.** A Vydac 218 TPC18 column; a mobile phase CH_3OH; a flow rate 1.0 ml/min; a retention time 2.75 min; UV detection was carried out at λ 254 nm; a purity was 98.8±0.8%.

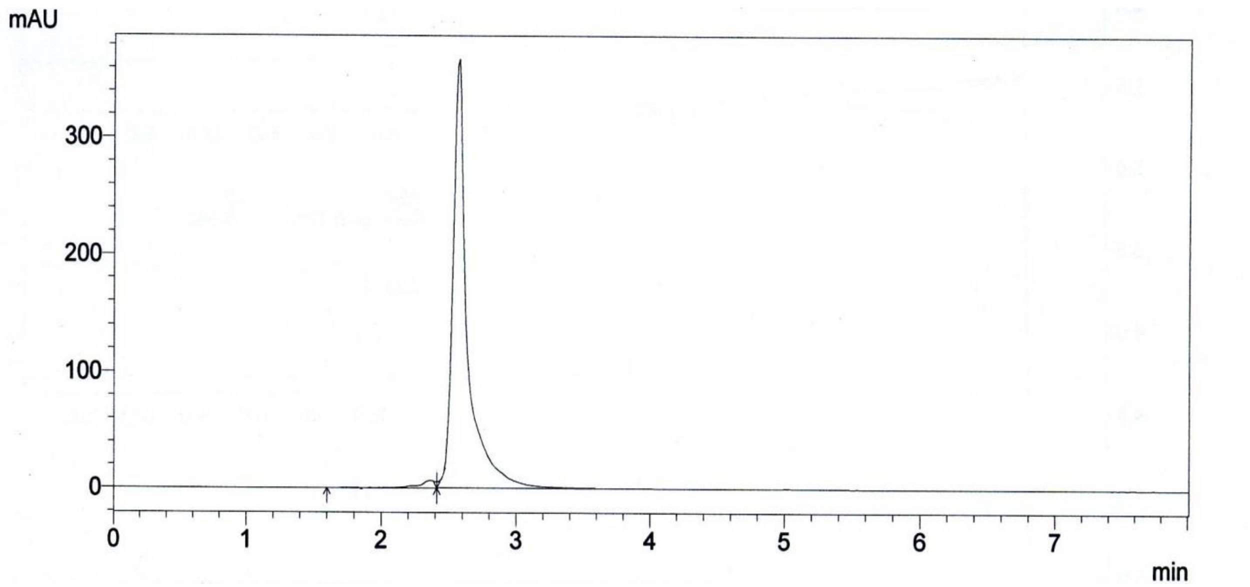

Supplemental Figure S39. HPLC for compound **15.** A Vydac 218 TPC18 column; a mobile phase CH_3OH; a flow rate 1.0 ml/min; a retention time 2.66 min; UV detection was carried out at λ 254 nm; a purity was 97.5±0.8%.

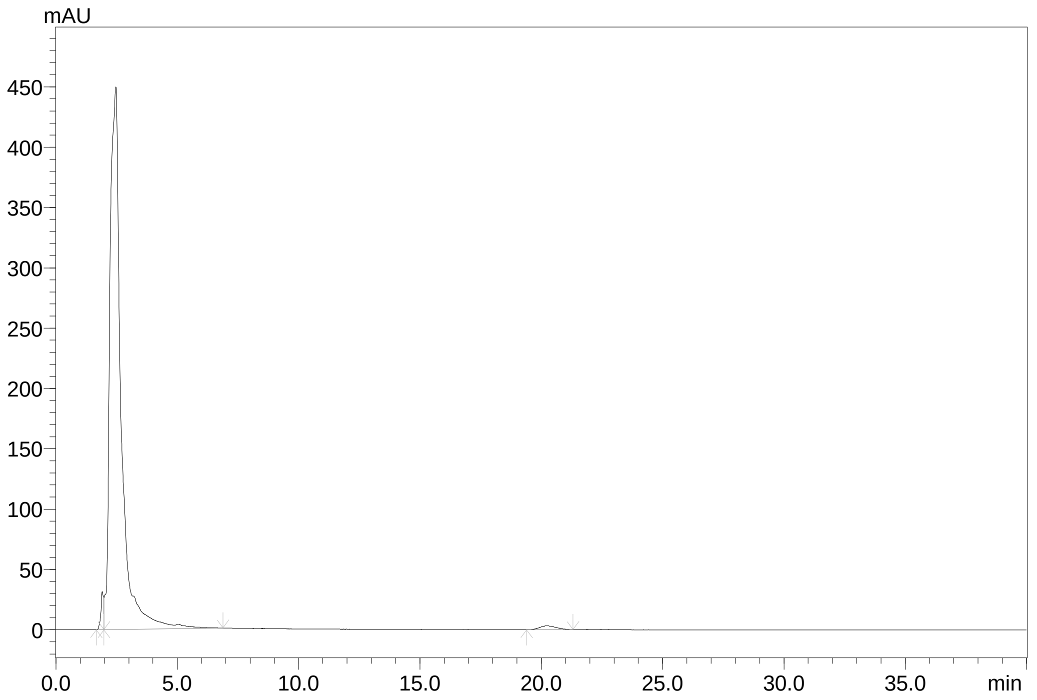

Supplemental Figure S40. HPLC for compound **16.** A Zorbax RX-C18 column; a mobile phase CH_3OH; a flow rate 1.0 ml/min; a retention time 2.44 min; UV detection was carried out at λ 254 nm; a purity was 97.2±0.8%.

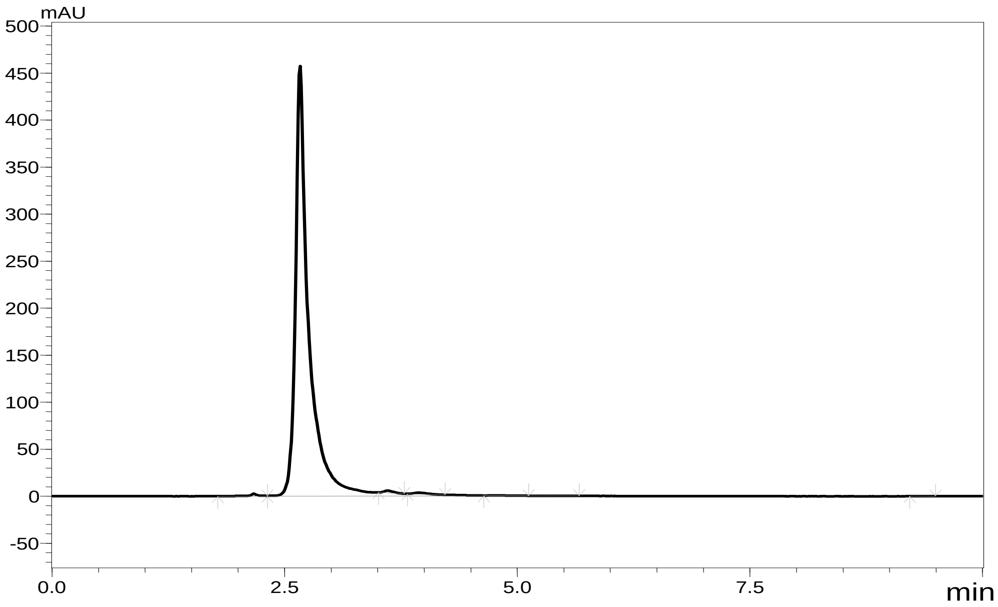

Supplemental Figure S41. HPLC for compound **17.** A Vydac 218 TPC18 column; a mobile phase CH_3OH; a flow rate 1.0 ml/min; a retention time 2.66 min; UV detection was carried out at λ 254 nm; a purity was 98.0±0.8%.

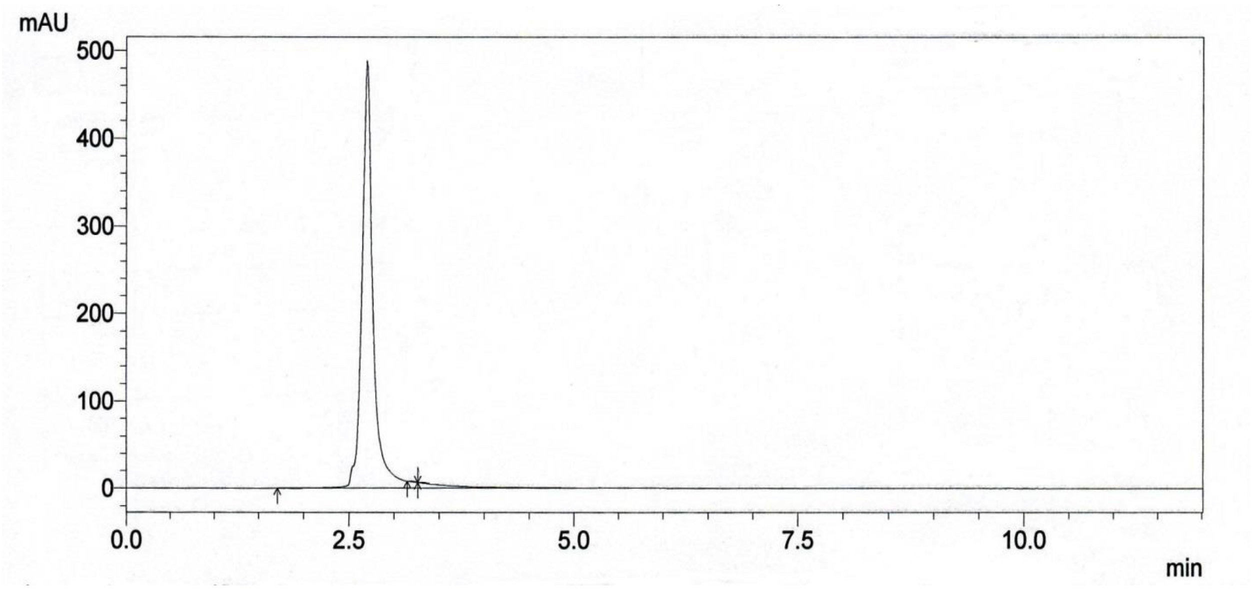

Supplemental Figure S42. HPLC for compound **18.** A Vydac 218 TPC18 column; a mobile phase CH_3OH; a flow rate 1.0 ml/min; a retention time 2.70 min; UV detection was carried out at λ 254 nm; a purity was 98.7±0.8%.

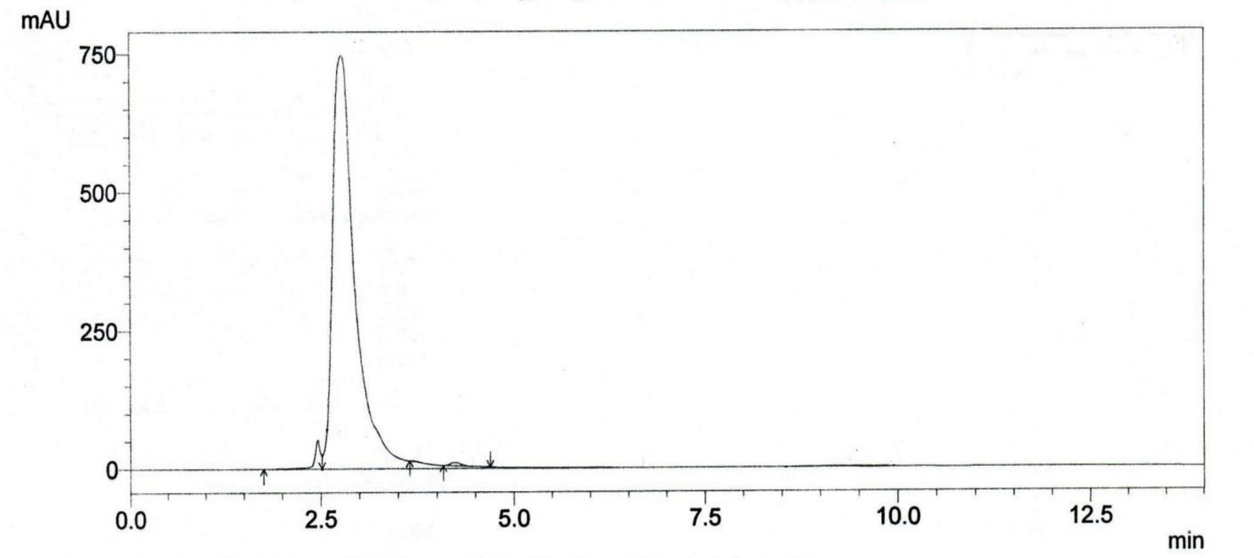

Supplemental Figure S43. HPLC for compound **19.** A Vydac 218 TPC18 column; a mobile phase CH_3OH; a flow rate 1.0 ml/min; a retention time 2.78 min; UV detection was carried out at λ 254 nm; a purity was 95.8±0.8%.

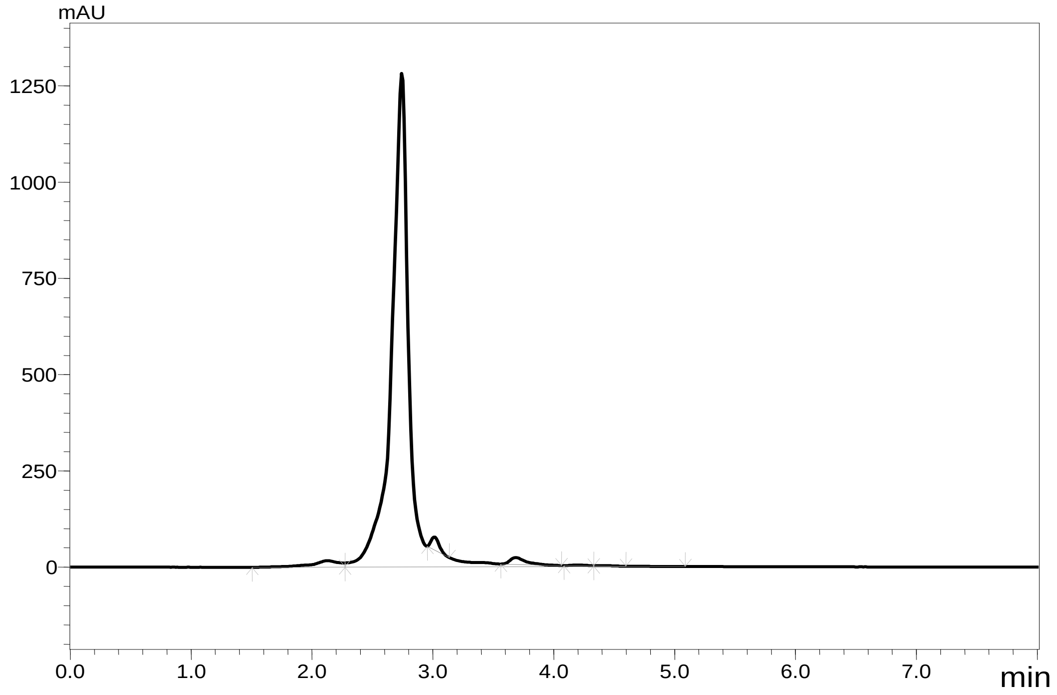

Supplemental Figure S44. HPLC for compound **20.** A Vydac 218 TPC18 column; a mobile phase CH_3OH; a flow rate 1.0 ml/min; a retention time 2.74 min; UV detection was carried out at λ 254 nm; a purity was 96.5±0.8%.

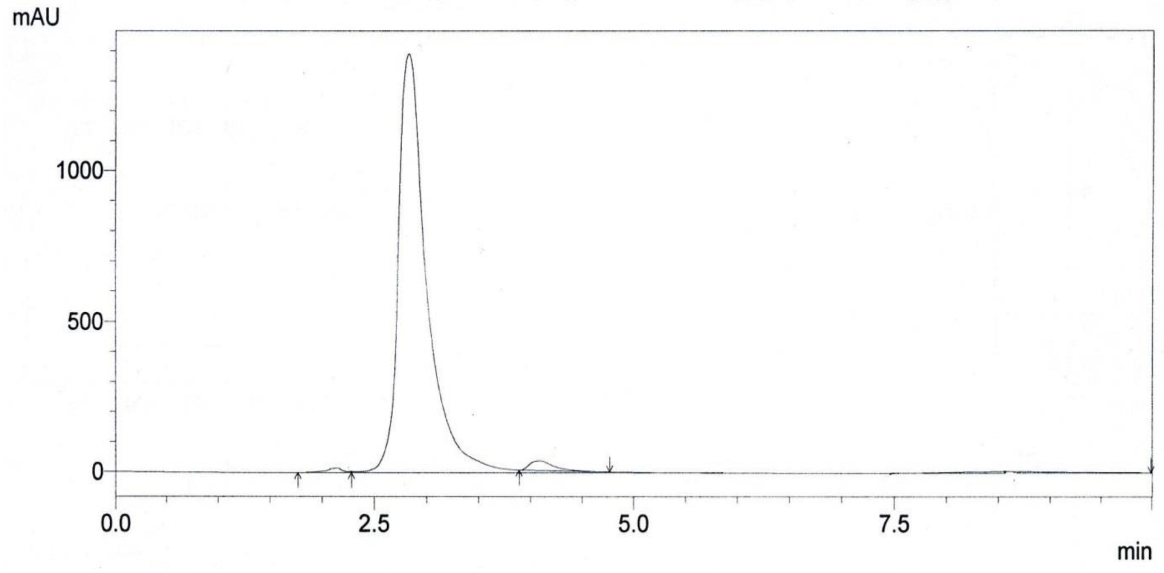

Supplemental Figure S45. HPLC for compound **21**. A Vydac 218 TPC18 column; a mobile phase CH_3OH; a flow rate 1.0 ml/min; a retention time 2.83 min; UV detection was carried out at λ 254 nm; a purity was 95.8±0.8%.
